# Supplementary material for: Ploidy Variation and Its Implications for Reproduction and Population Dynamics in Two Sympatric Hawaiian Coral Species
Source: Genome Biol Evol. 2023 Aug 11;15(8):evad149. doi: 10.1093/gbe/evad149 (PMC10445776; doi:10.1093/gbe/evad149)
Supplement: evad149_Supplementary_Data [file evad149_supplementary_data.zip › Data_S1.pdf]

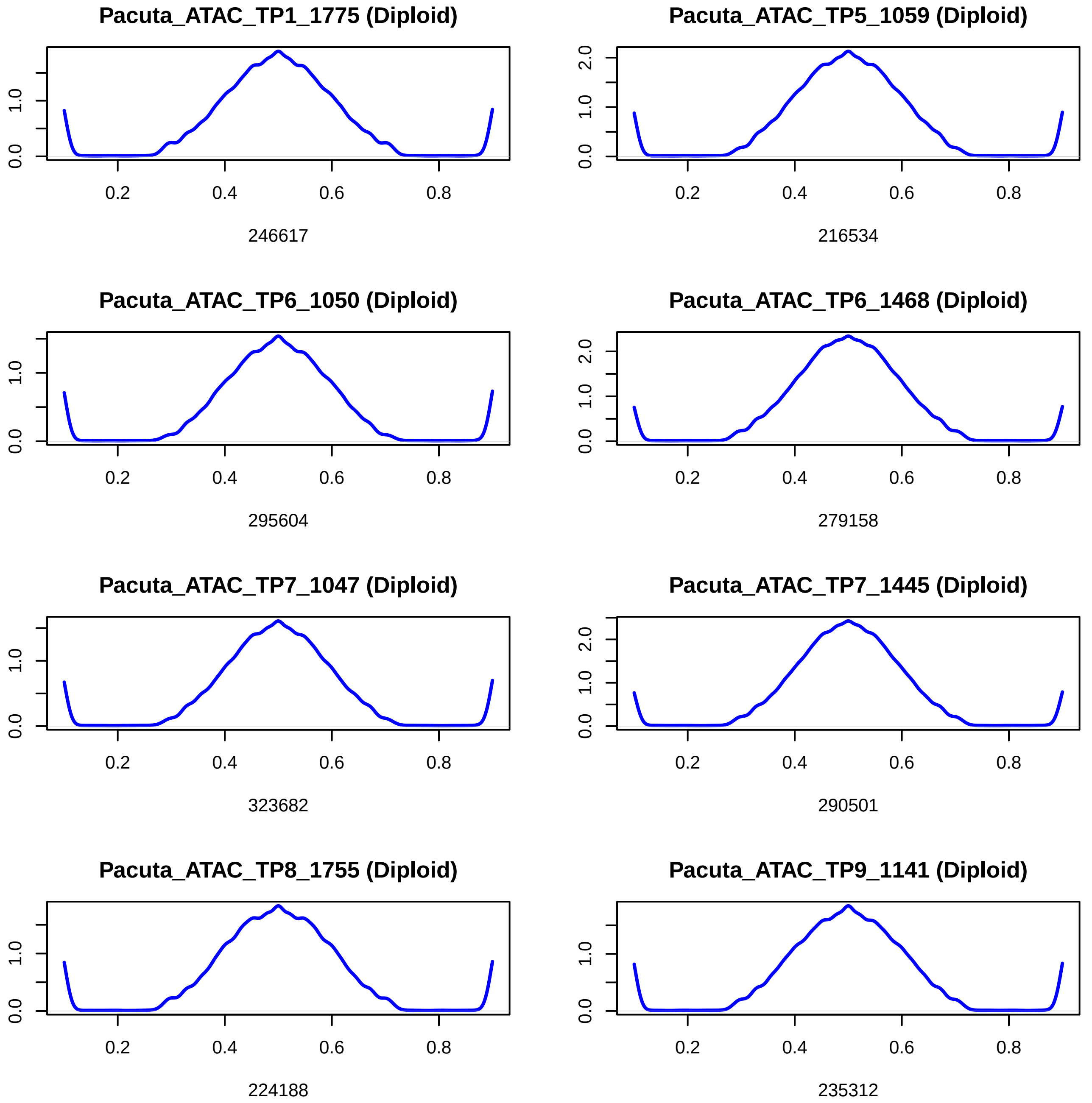
Pacuta_ATAC_TP1_1775 (Diploid)
0.0
1.0
0.2
0.4
0.6
0.8
246617
Pacuta_ATAC_TP5_1059 (Diploid)
0.0
1.0
2.0
0.2
0.4
0.6
0.8
216534
Pacuta_ATAC_TP6_1050 (Diploid)
0.0
1.0
0.2
0.4
0.6
0.8
295604
Pacuta_ATAC_TP6_1468 (Diploid)
0.0
1.0
2.0
0.2
0.4
0.6
0.8
279158
Pacuta_ATAC_TP7_1047 (Diploid)
0.0
1.0
0.2
0.4
0.6
0.8
323682
Pacuta_ATAC_TP7_1445 (Diploid)
0.0
1.0
2.0
0.2
0.4
0.6
0.8
290501
Pacuta_ATAC_TP8_1755 (Diploid)
0.0
1.0
0.2
0.4
0.6
0.8
224188
Pacuta_ATAC_TP9_1141 (Diploid)
0.0
1.0
0.2
0.4
0.6
0.8
235312

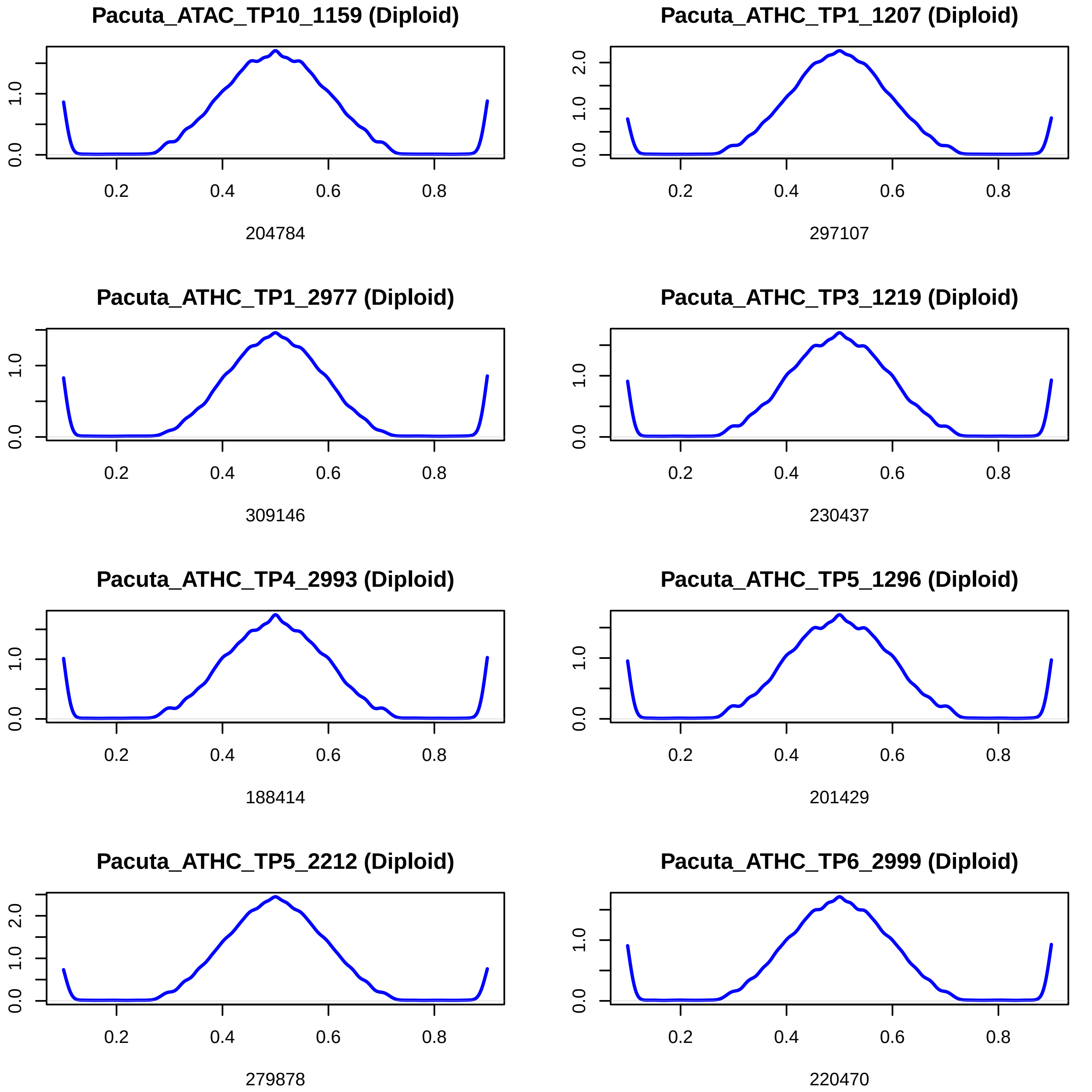
Pacuta_ATAC_TP10_1159 (Diploid)
204784
Pacuta_ATHC_TP1_1207 (Diploid)
297107
Pacuta_ATHC_TP1_2977 (Diploid)
309146
Pacuta_ATHC_TP3_1219 (Diploid)
230437
Pacuta_ATHC_TP4_2993 (Diploid)
188414
Pacuta_ATHC_TP5_1296 (Diploid)
201429
Pacuta_ATHC_TP5_2212 (Diploid)
279878
Pacuta_ATHC_TP6_2999 (Diploid)
220470
0.0
1.0
2.0
0.2
0.4
0.6
0.8

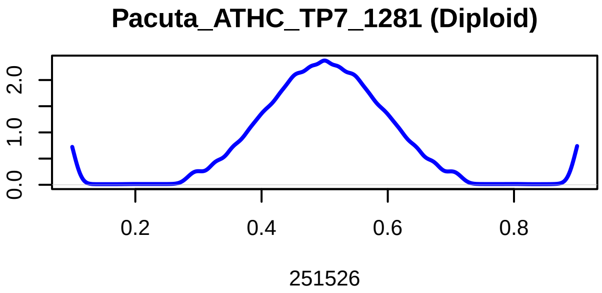
Pacuta_ATHC_TP7_1281 (Diploid)
0.0
1.0
2.0
0.2
0.4
0.6
0.8
251526

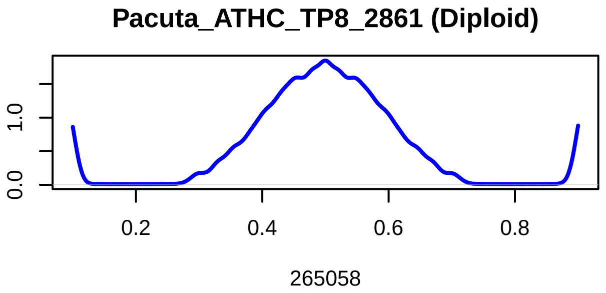
Pacuta_ATHC_TP8_2861 (Diploid)
0.0
1.0
0.2
0.4
0.6
0.8
265058

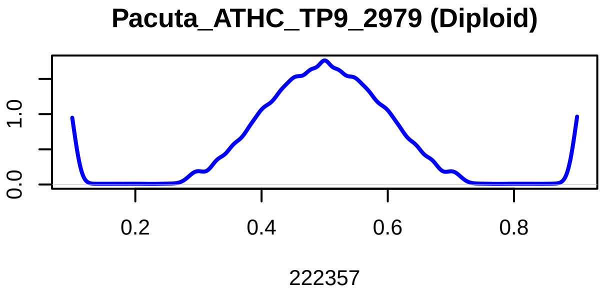
Pacuta_ATHC_TP9_2979 (Diploid)
0.0
1.0
0.2
0.4
0.6
0.8
222357

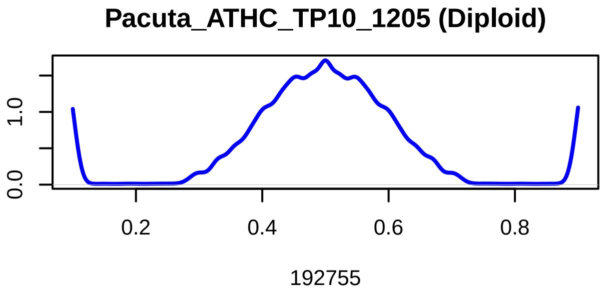
Pacuta_ATHC_TP10_1205 (Diploid)
0.0
1.0
0.2
0.4
0.6
0.8
192755

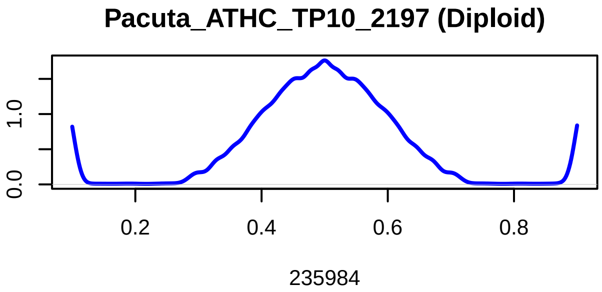
Pacuta_ATHC_TP10_2197 (Diploid)
0.0
1.0
0.2
0.4
0.6
0.8
235984

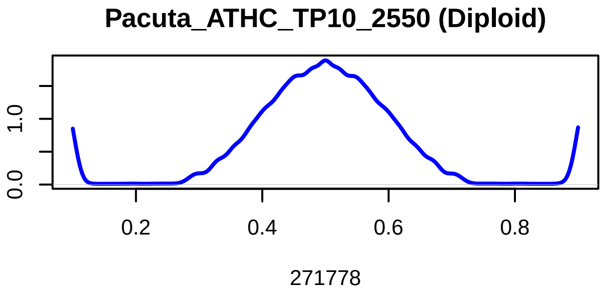
Pacuta_ATHC_TP10_2550 (Diploid)
0.0
1.0
0.2
0.4
0.6
0.8
271778

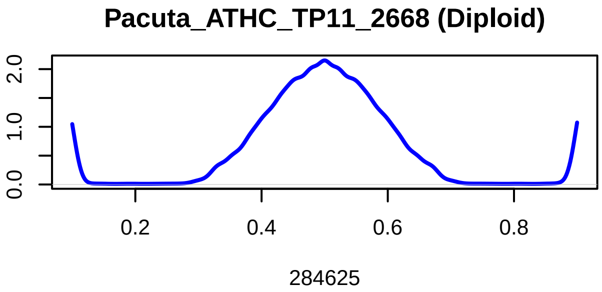
Pacuta_ATHC_TP11_2668 (Diploid)
0.0
1.0
2.0
0.2
0.4
0.6
0.8
284625

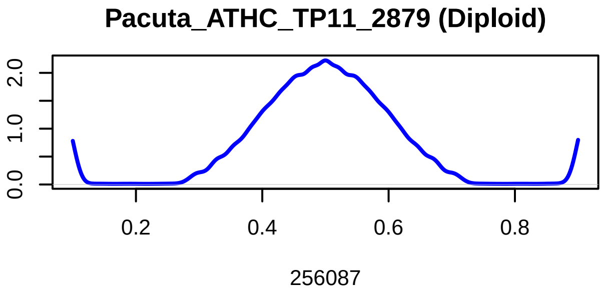
Pacuta_ATHC_TP11_2879 (Diploid)
0.0
1.0
2.0
0.2
0.4
0.6
0.8
256087

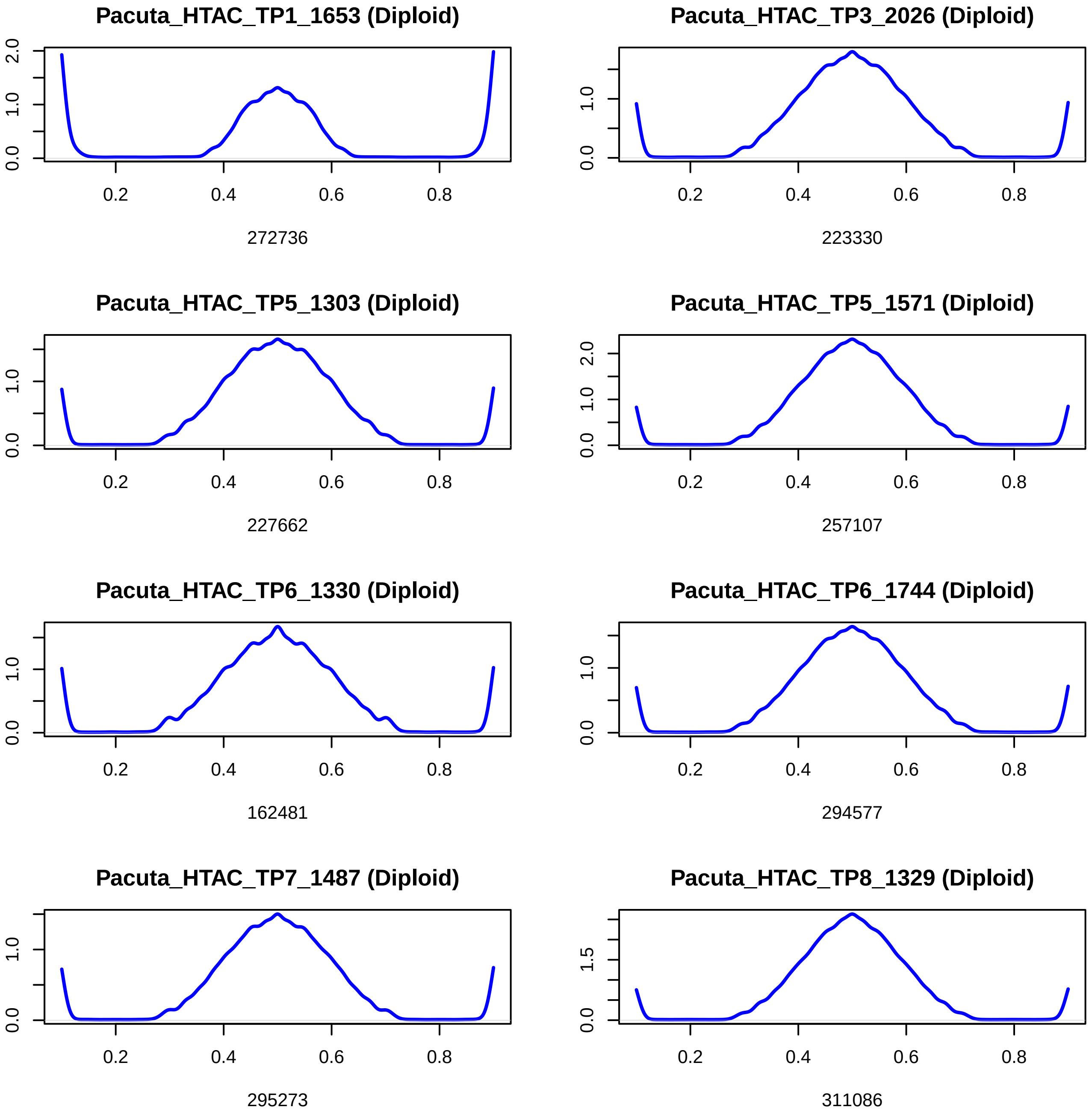
Pacuta_HTAC_TP1_1653 (Diploid)
0.0
1.0
2.0
0.2
0.4
0.6
0.8
272736
Pacuta_HTAC_TP3_2026 (Diploid)
0.0
1.0
0.2
0.4
0.6
0.8
223330
Pacuta_HTAC_TP5_1303 (Diploid)
0.0
1.0
0.2
0.4
0.6
0.8
227662
Pacuta_HTAC_TP5_1571 (Diploid)
0.0
1.0
2.0
0.2
0.4
0.6
0.8
257107
Pacuta_HTAC_TP6_1330 (Diploid)
0.0
1.0
0.2
0.4
0.6
0.8
162481
Pacuta_HTAC_TP6_1744 (Diploid)
0.0
1.0
0.2
0.4
0.6
0.8
294577
Pacuta_HTAC_TP7_1487 (Diploid)
0.0
1.0
0.2
0.4
0.6
0.8
295273
Pacuta_HTAC_TP8_1329 (Diploid)
0.0
1.5
0.2
0.4
0.6
0.8
311086

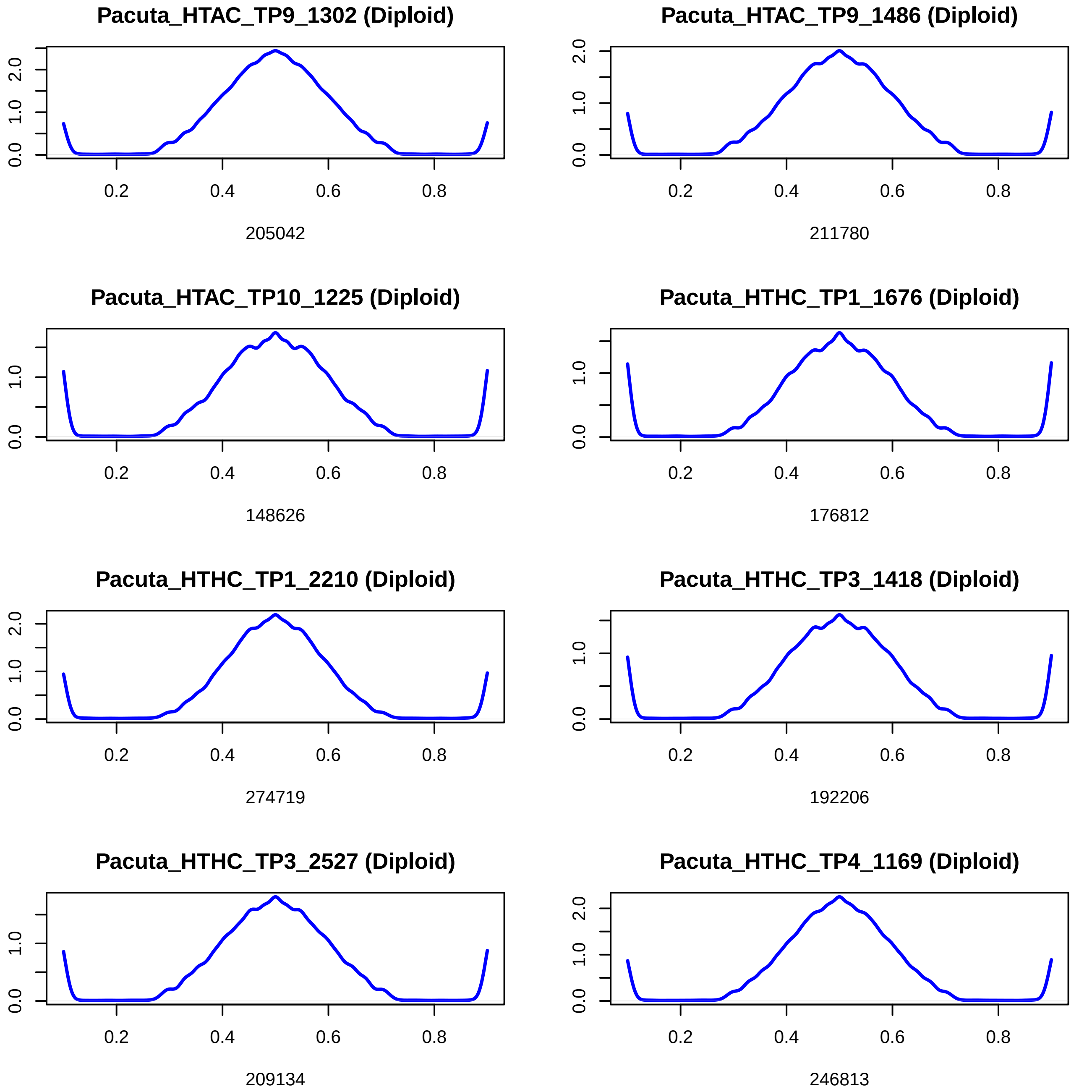
Pacuta_HTAC_TP9_1302 (Diploid)
205042
Pacuta_HTAC_TP9_1486 (Diploid)
211780
Pacuta_HTAC_TP10_1225 (Diploid)
148626
Pacuta_HTHC_TP1_1676 (Diploid)
176812
Pacuta_HTHC_TP1_2210 (Diploid)
274719
Pacuta_HTHC_TP3_1418 (Diploid)
192206
Pacuta_HTHC_TP3_2527 (Diploid)
209134
Pacuta_HTHC_TP4_1169 (Diploid)
246813
0.0
1.0
2.0
0.2
0.4
0.6
0.8

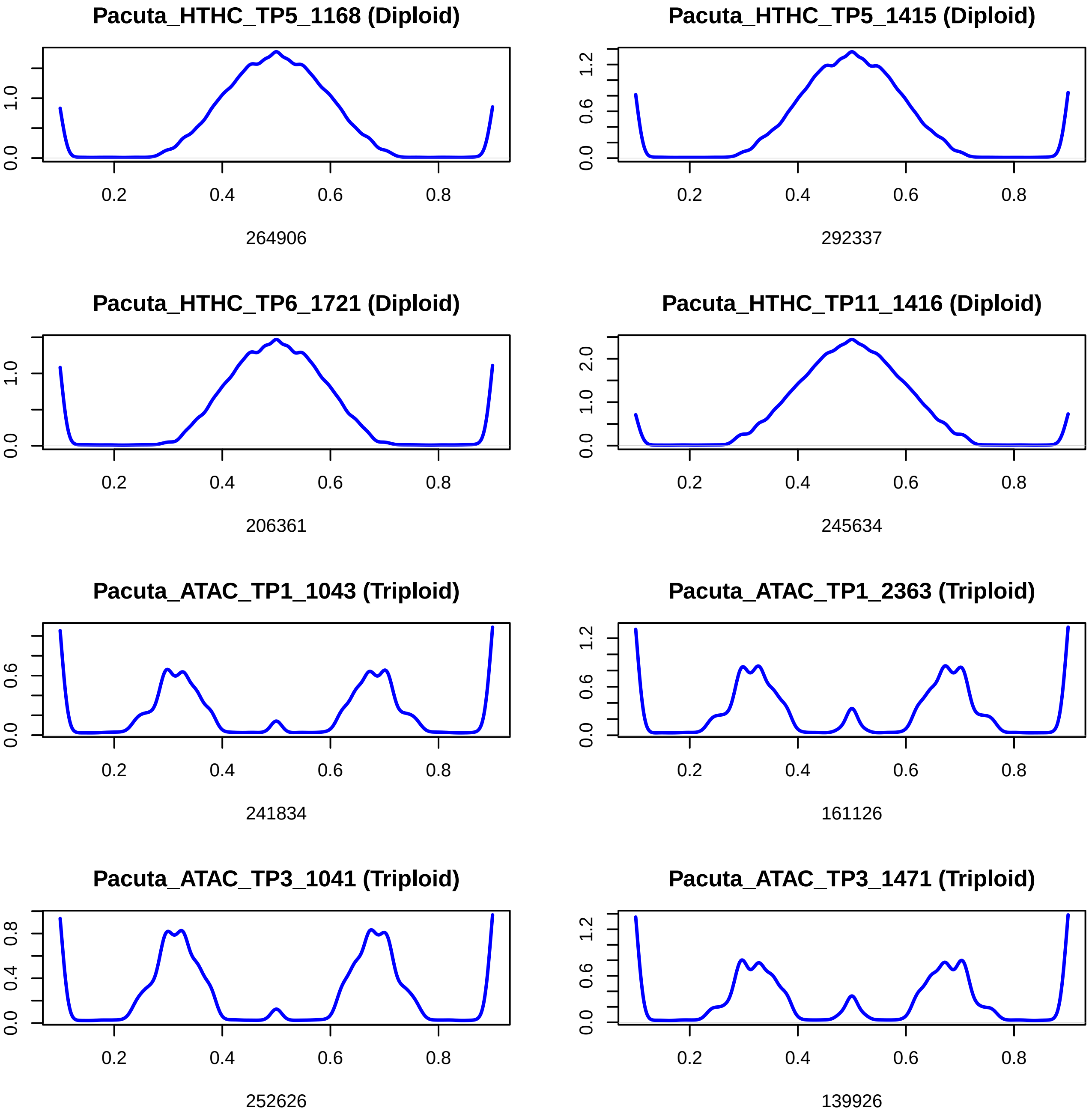
Pacuta_HTHC_TP5_1168 (Diploid)
0.0
1.0
0.2
0.4
0.6
0.8
264906
Pacuta_HTHC_TP5_1415 (Diploid)
0.0
0.6
1.2
0.2
0.4
0.6
0.8
292337
Pacuta_HTHC_TP6_1721 (Diploid)
0.0
1.0
0.2
0.4
0.6
0.8
206361
Pacuta_HTHC_TP11_1416 (Diploid)
0.0
1.0
2.0
0.2
0.4
0.6
0.8
245634
Pacuta_ATAC_TP1_1043 (Triploid)
0.0
0.6
0.2
0.4
0.6
0.8
241834
Pacuta_ATAC_TP1_2363 (Triploid)
0.0
0.6
1.2
0.2
0.4
0.6
0.8
161126
Pacuta_ATAC_TP3_1041 (Triploid)
0.0
0.4
0.8
0.2
0.4
0.6
0.8
252626
Pacuta_ATAC_TP3_1471 (Triploid)
0.0
0.6
1.2
0.2
0.4
0.6
0.8
139926

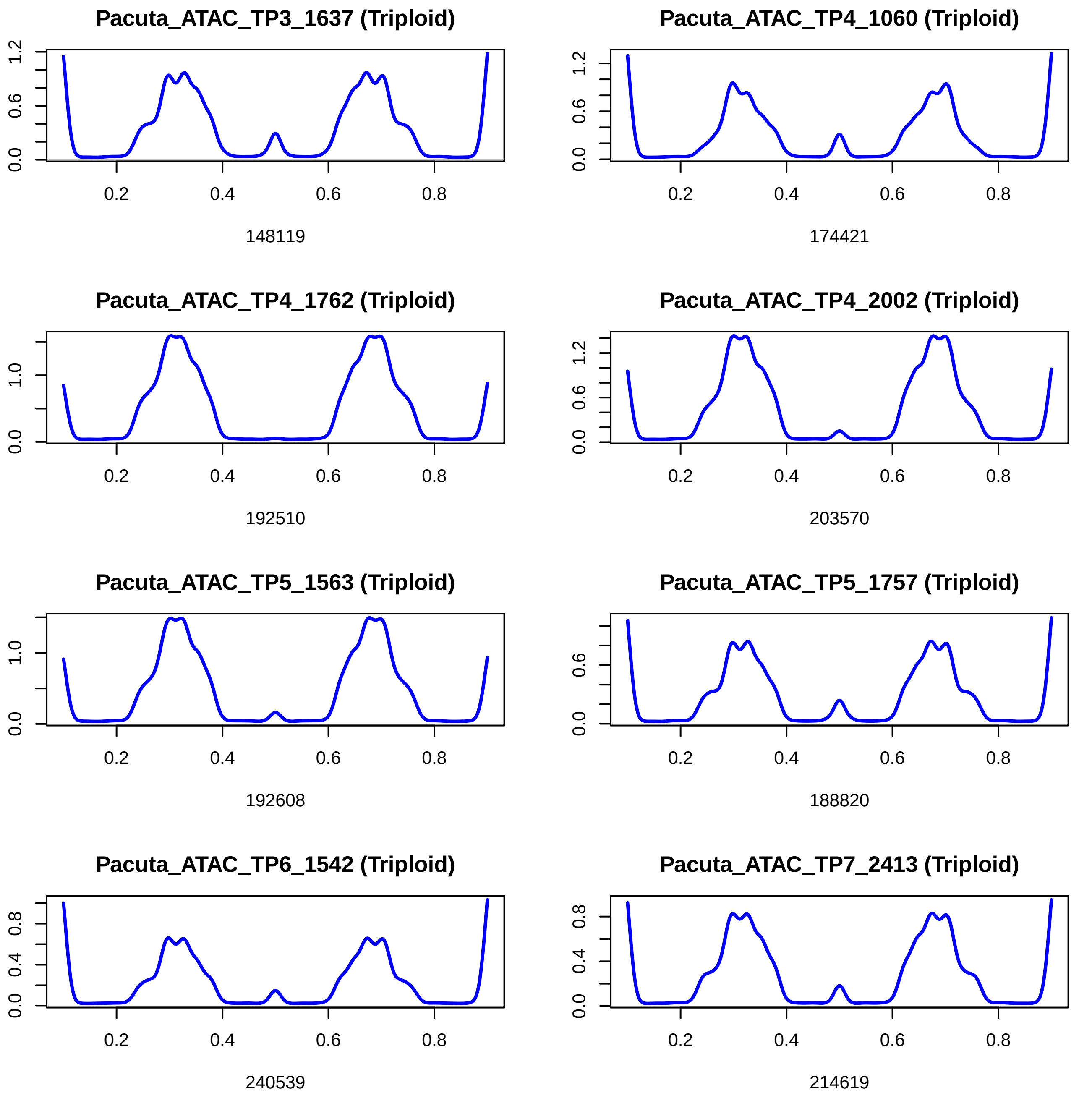

Pacuta_ATAC_TP3_1637 (Triploid)
0.0
0.6
1.2
0.2
0.4
0.6
0.8
148119
Pacuta_ATAC_TP4_1060 (Triploid)
0.0
0.6
1.2
0.2
0.4
0.6
0.8
174421
Pacuta_ATAC_TP4_1762 (Triploid)
0.0
1.0
0.2
0.4
0.6
0.8
192510
Pacuta_ATAC_TP4_2002 (Triploid)
0.0
0.6
1.2
0.2
0.4
0.6
0.8
203570
Pacuta_ATAC_TP5_1563 (Triploid)
0.0
1.0
0.2
0.4
0.6
0.8
192608
Pacuta_ATAC_TP5_1757 (Triploid)
0.0
0.6
0.2
0.4
0.6
0.8
188820
Pacuta_ATAC_TP6_1542 (Triploid)
0.0
0.4
0.8
0.2
0.4
0.6
0.8
240539
Pacuta_ATAC_TP7_2413 (Triploid)
0.0
0.4
0.8
0.2
0.4
0.6
0.8
214619

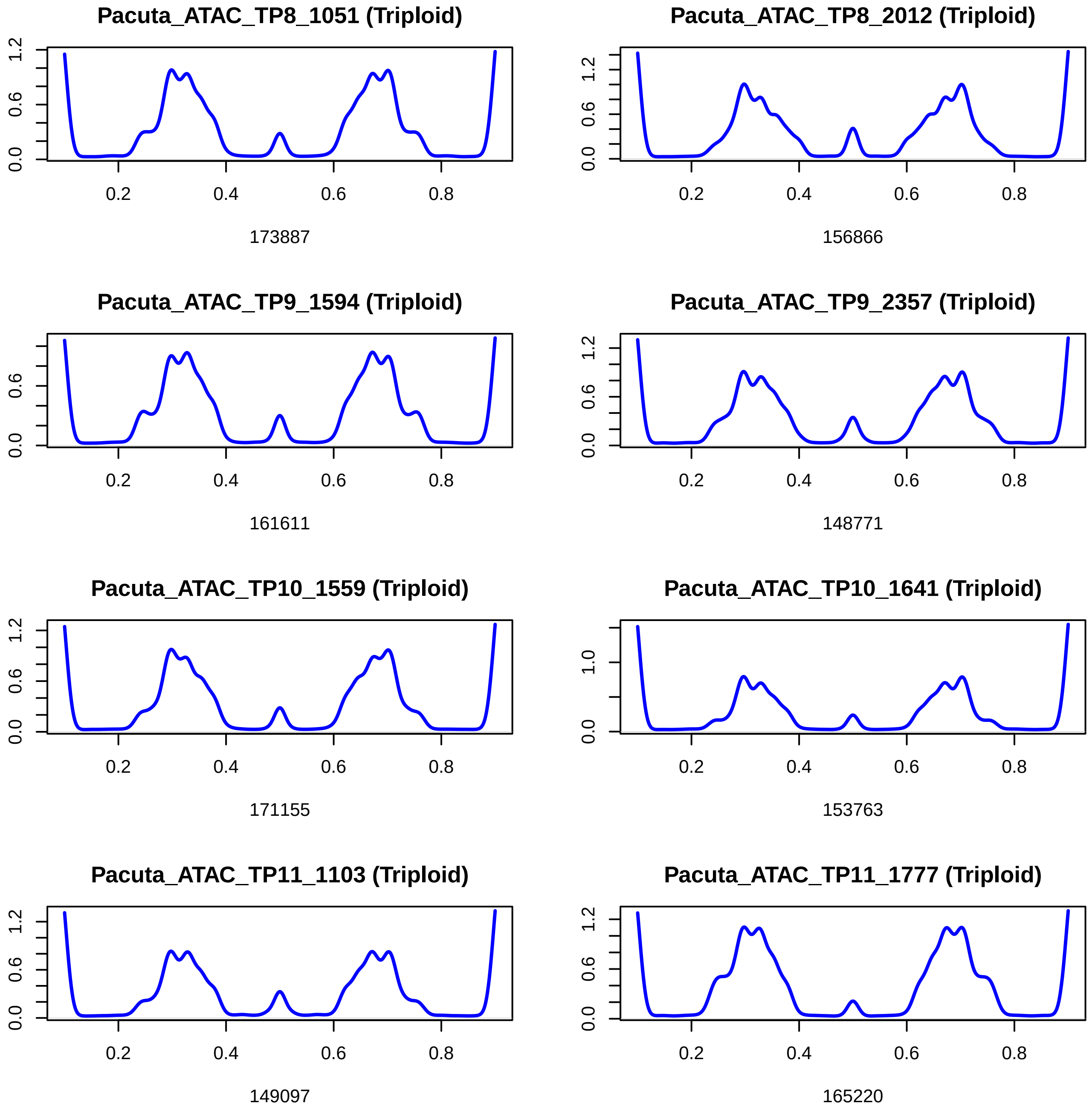

Pacuta_ATAC_TP8_1051 (Triploid)
0.0
0.6
1.2
0.2
0.4
0.6
0.8
173887
Pacuta_ATAC_TP8_2012 (Triploid)
0.0
0.6
1.2
0.2
0.4
0.6
0.8
156866
Pacuta_ATAC_TP9_1594 (Triploid)
0.0
0.6
0.2
0.4
0.6
0.8
161611
Pacuta_ATAC_TP9_2357 (Triploid)
0.0
0.6
1.2
0.2
0.4
0.6
0.8
148771
Pacuta_ATAC_TP10_1559 (Triploid)
0.0
0.6
1.2
0.2
0.4
0.6
0.8
171155
Pacuta_ATAC_TP10_1641 (Triploid)
0.0
1.0
0.2
0.4
0.6
0.8
153763
Pacuta_ATAC_TP11_1103 (Triploid)
0.0
0.6
1.2
0.2
0.4
0.6
0.8
149097
Pacuta_ATAC_TP11_1777 (Triploid)
0.0
0.6
1.2
0.2
0.4
0.6
0.8
165220

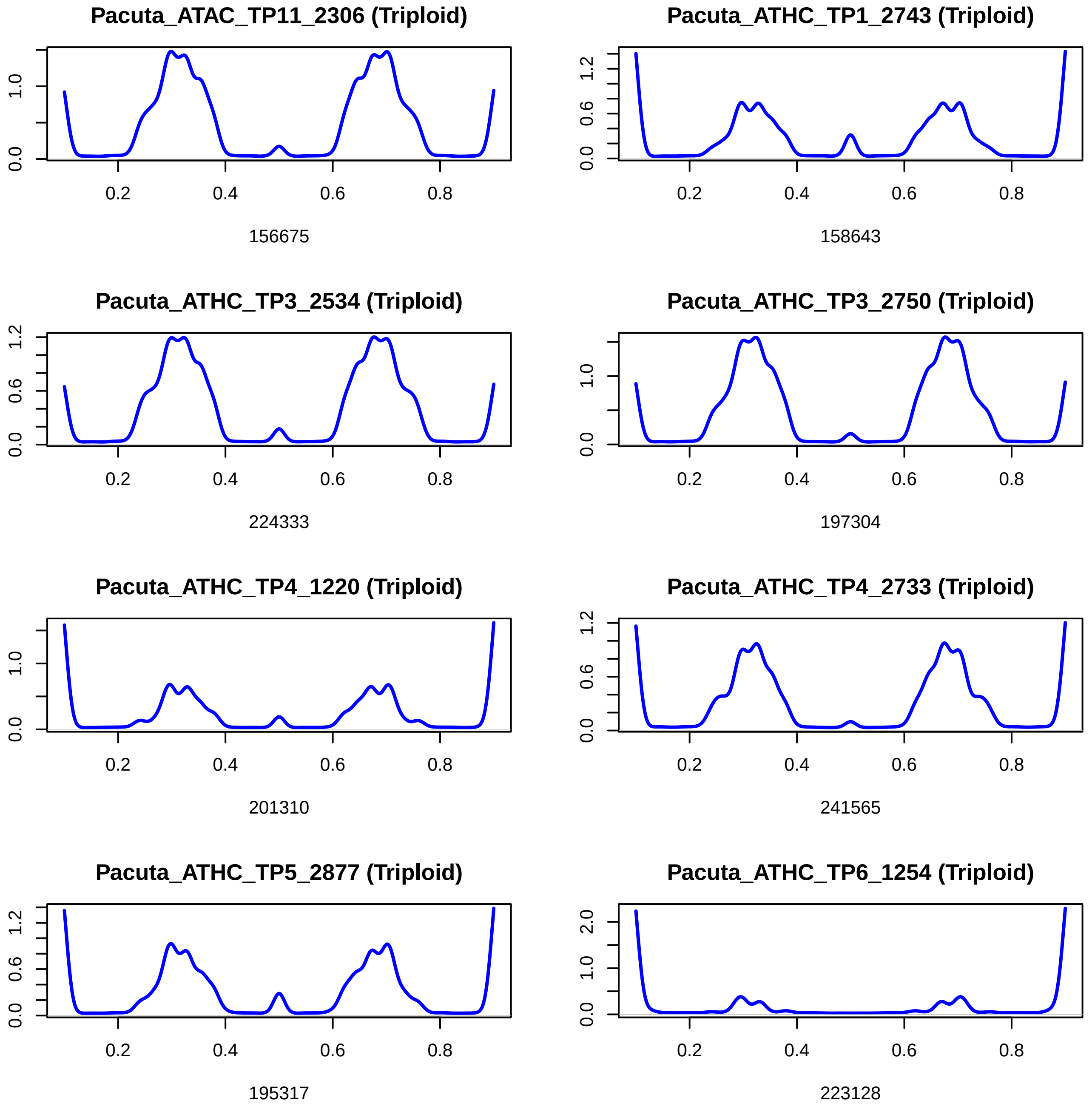
Pacuta_ATAC_TP11_2306 (Triploid)
0.0
1.0
0.2
0.4
0.6
0.8
156675
Pacuta_ATHC_TP1_2743 (Triploid)
0.0
0.6
1.2
0.2
0.4
0.6
0.8
158643
Pacuta_ATHC_TP3_2534 (Triploid)
0.0
0.6
1.2
0.2
0.4
0.6
0.8
224333
Pacuta_ATHC_TP3_2750 (Triploid)
0.0
1.0
0.2
0.4
0.6
0.8
197304
Pacuta_ATHC_TP4_1220 (Triploid)
0.0
1.0
0.2
0.4
0.6
0.8
201310
Pacuta_ATHC_TP4_2733 (Triploid)
0.0
0.6
1.2
0.2
0.4
0.6
0.8
241565
Pacuta_ATHC_TP5_2877 (Triploid)
0.0
0.6
1.2
0.2
0.4
0.6
0.8
195317
Pacuta_ATHC_TP6_1254 (Triploid)
0.0
1.0
2.0
0.2
0.4
0.6
0.8
223128

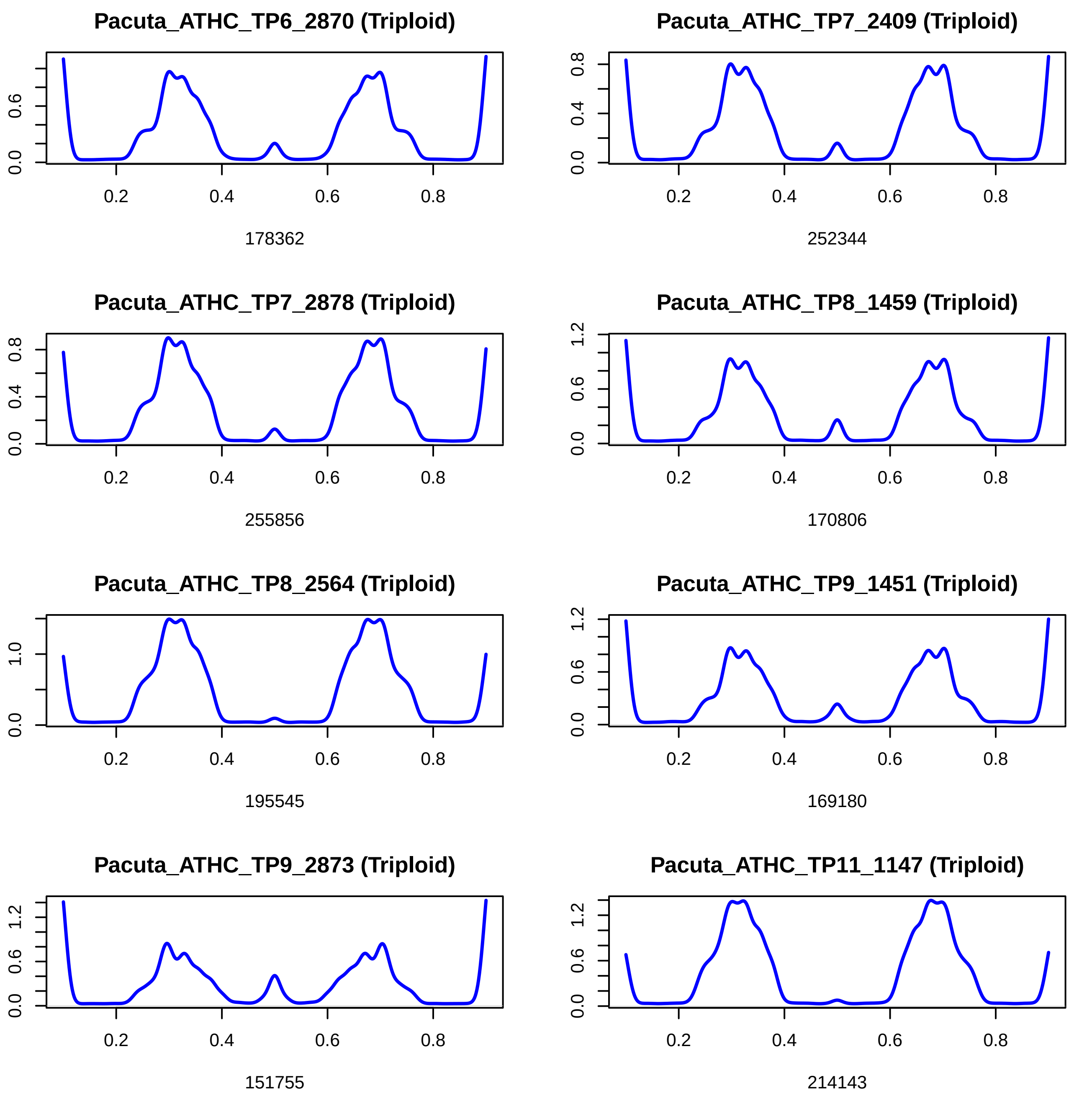
Pacuta_ATHC_TP6_2870 (Triploid)
0.0
0.6
0.2
0.4
0.6
0.8
178362
Pacuta_ATHC_TP7_2409 (Triploid)
0.0
0.4
0.8
0.2
0.4
0.6
0.8
252344
Pacuta_ATHC_TP7_2878 (Triploid)
0.0
0.4
0.8
0.2
0.4
0.6
0.8
255856
Pacuta_ATHC_TP8_1459 (Triploid)
0.0
0.6
1.2
0.2
0.4
0.6
0.8
170806
Pacuta_ATHC_TP8_2564 (Triploid)
0.0
1.0
0.2
0.4
0.6
0.8
195545
Pacuta_ATHC_TP9_1451 (Triploid)
0.0
0.6
1.2
0.2
0.4
0.6
0.8
169180
Pacuta_ATHC_TP9_2873 (Triploid)
0.0
0.6
1.2
0.2
0.4
0.6
0.8
151755
Pacuta_ATHC_TP11_1147 (Triploid)
0.0
0.6
1.2
0.2
0.4
0.6
0.8
214143

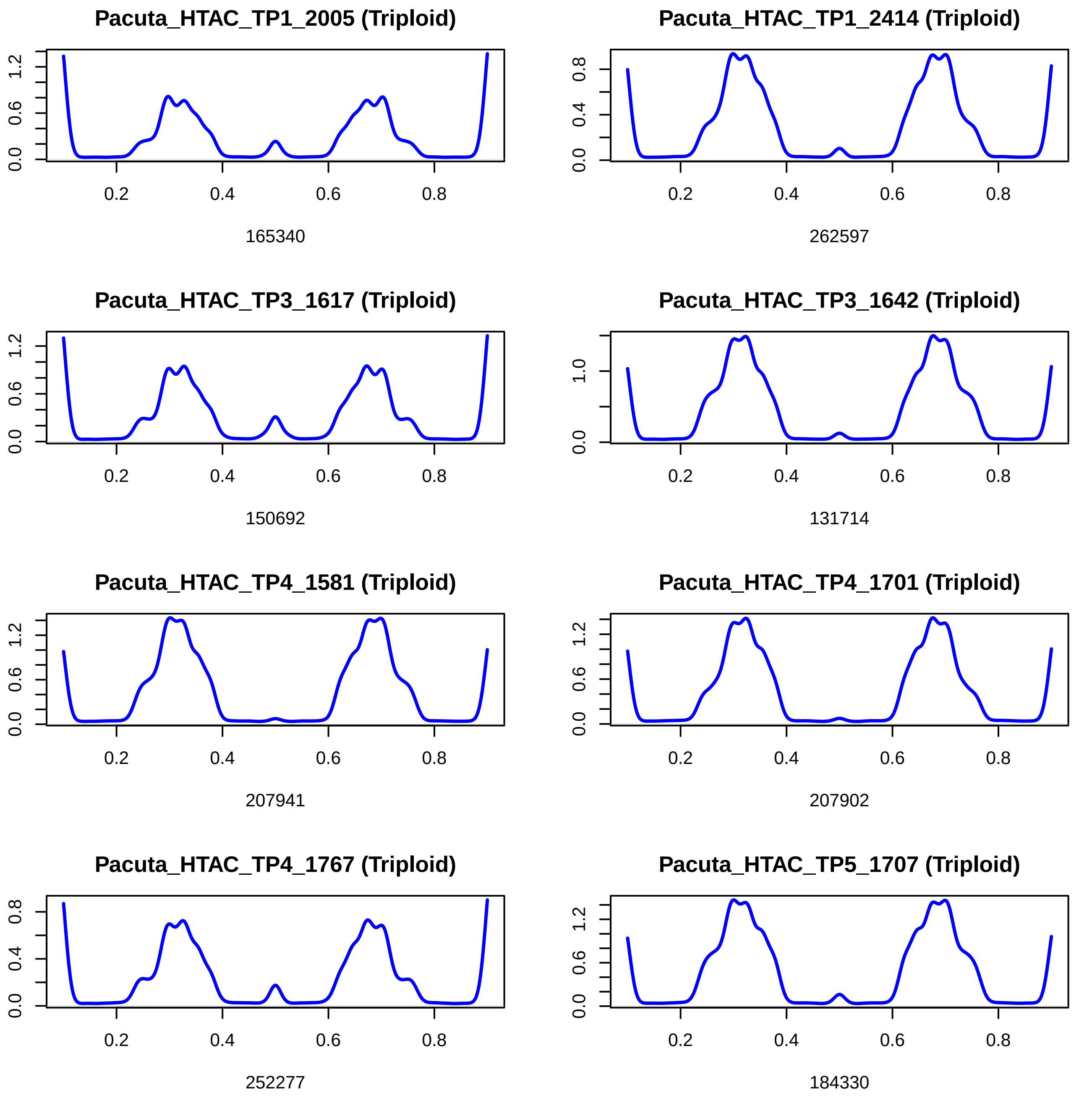
Pacuta_HTAC_TP1_2005 (Triploid)
165340
Pacuta_HTAC_TP1_2414 (Triploid)
262597
Pacuta_HTAC_TP3_1617 (Triploid)
150692
Pacuta_HTAC_TP3_1642 (Triploid)
131714
Pacuta_HTAC_TP4_1581 (Triploid)
207941
Pacuta_HTAC_TP4_1701 (Triploid)
207902
Pacuta_HTAC_TP4_1767 (Triploid)
252277
Pacuta_HTAC_TP5_1707 (Triploid)
184330
0.0
0.4
0.6
0.8
1.0
1.2
0.2
0.4
0.6
0.8

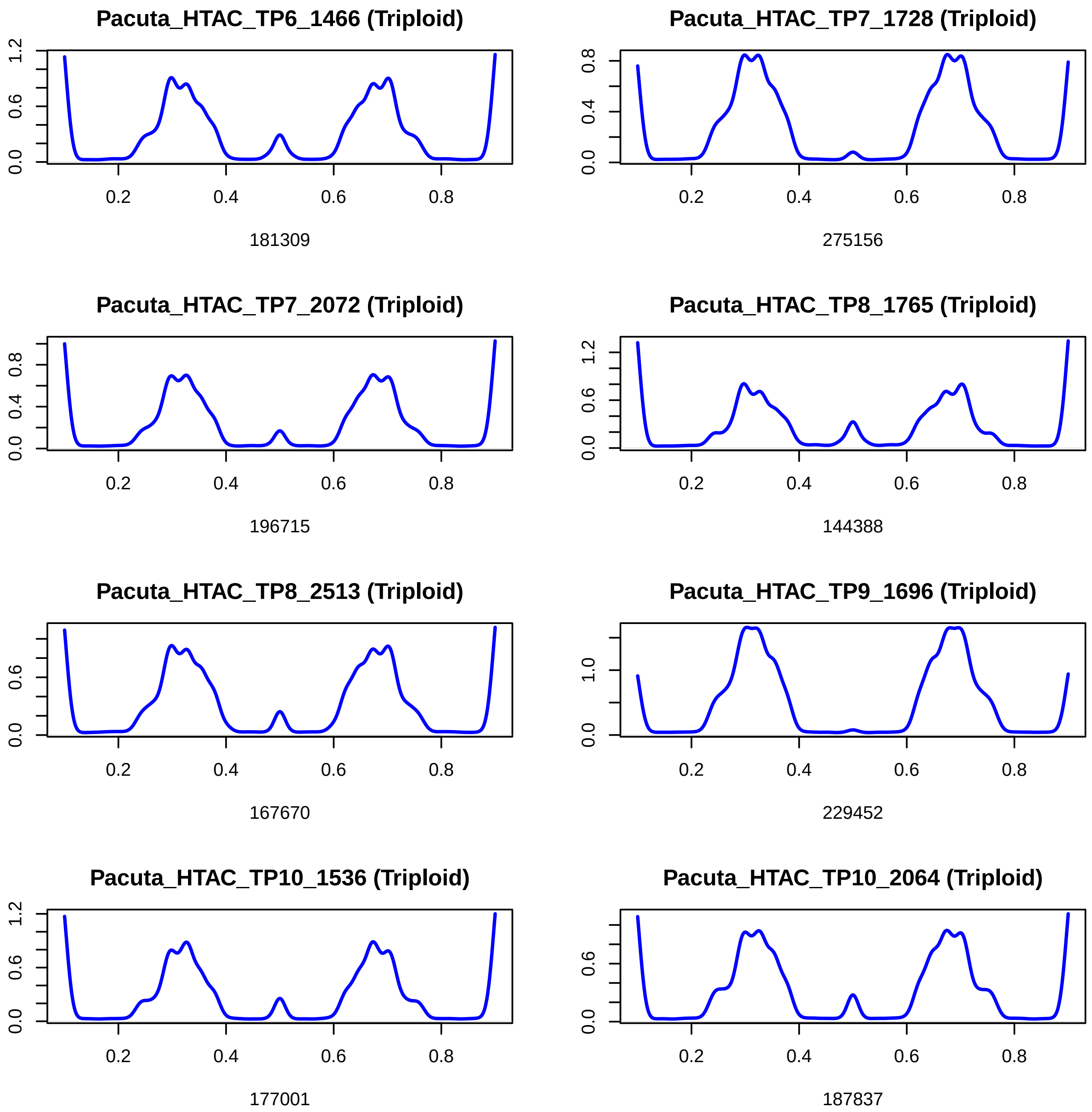
Pacuta_HTAC_TP6_1466 (Triploid)
0.0
0.6
1.2
0.2
0.4
0.6
0.8
181309
Pacuta_HTAC_TP7_1728 (Triploid)
0.0
0.4
0.8
0.2
0.4
0.6
0.8
275156
Pacuta_HTAC_TP7_2072 (Triploid)
0.0
0.4
0.8
0.2
0.4
0.6
0.8
196715
Pacuta_HTAC_TP8_1765 (Triploid)
0.0
0.6
1.2
0.2
0.4
0.6
0.8
144388
Pacuta_HTAC_TP8_2513 (Triploid)
0.0
0.6
0.2
0.4
0.6
0.8
167670
Pacuta_HTAC_TP9_1696 (Triploid)
0.0
1.0
0.2
0.4
0.6
0.8
229452
Pacuta_HTAC_TP10_1536 (Triploid)
0.0
0.6
1.2
0.2
0.4
0.6
0.8
177001
Pacuta_HTAC_TP10_2064 (Triploid)
0.0
0.6
0.2
0.4
0.6
0.8
187837

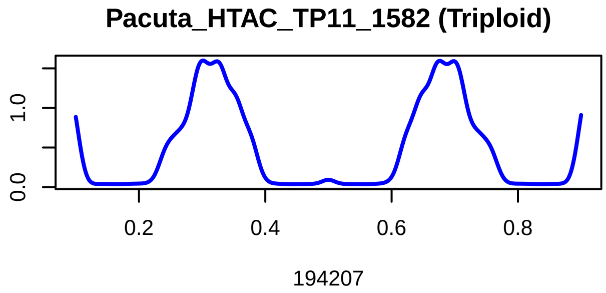
Pacuta_HTAC_TP11_1582 (Triploid)
0.0
1.0
0.2
0.4
0.6
0.8
194207

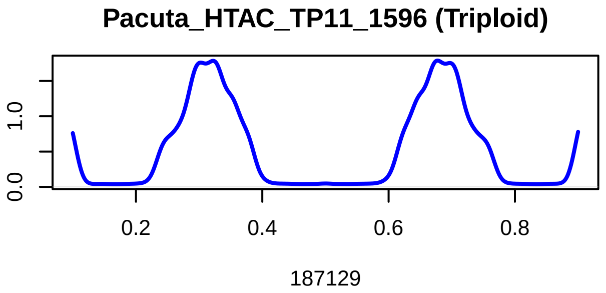
Pacuta_HTAC_TP11_1596 (Triploid)
0.0
1.0
0.2
0.4
0.6
0.8
187129

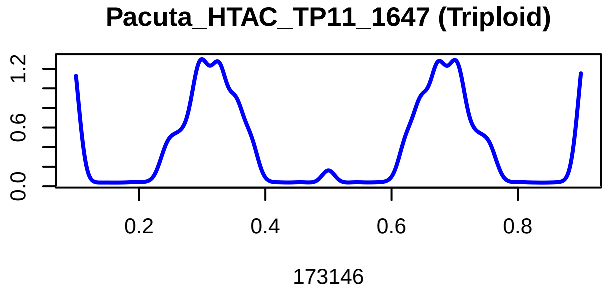
Pacuta_HTAC_TP11_1647 (Triploid)
0.0
0.6
1.2
0.2
0.4
0.6
0.8
173146

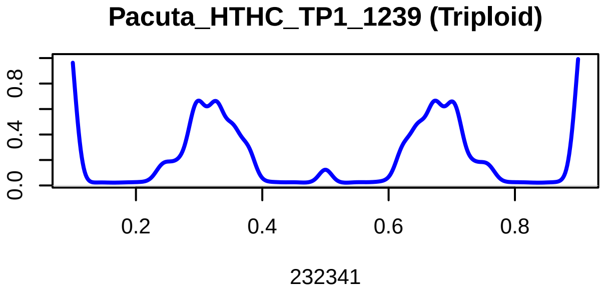
Pacuta_HTHC_TP1_1239 (Triploid)
0.0
0.4
0.8
0.2
0.4
0.6
0.8
232341

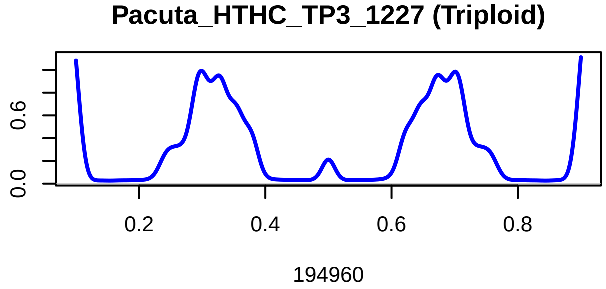
Pacuta_HTHC_TP3_1227 (Triploid)
0.0
0.6
0.2
0.4
0.6
0.8
194960

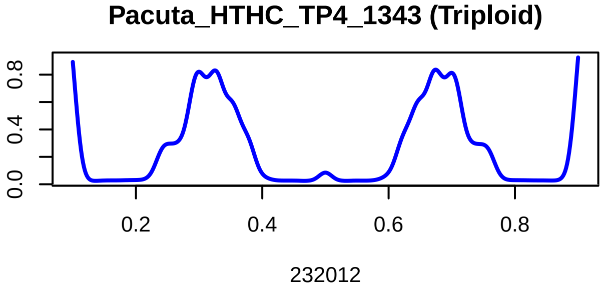
Pacuta_HTHC_TP4_1343 (Triploid)
0.0
0.4
0.8
0.2
0.4
0.6
0.8
232012

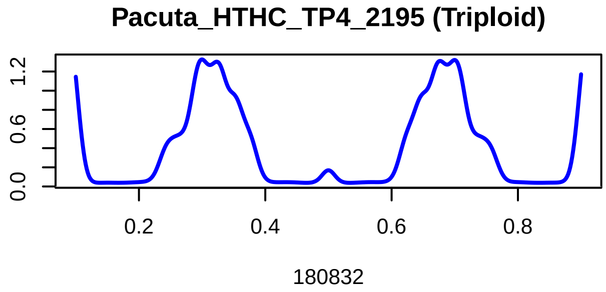
Pacuta_HTHC_TP4_2195 (Triploid)
0.0
0.6
1.2
0.2
0.4
0.6
0.8
180832

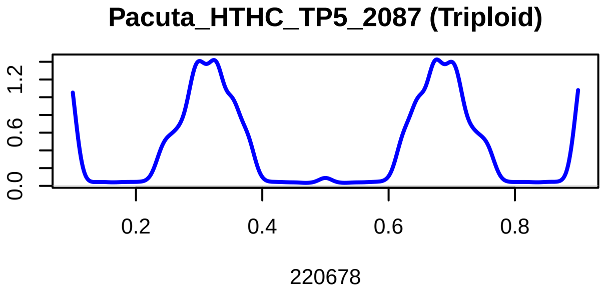
Pacuta_HTHC_TP5_2087 (Triploid)
0.0
0.6
1.2
0.2
0.4
0.6
0.8
220678

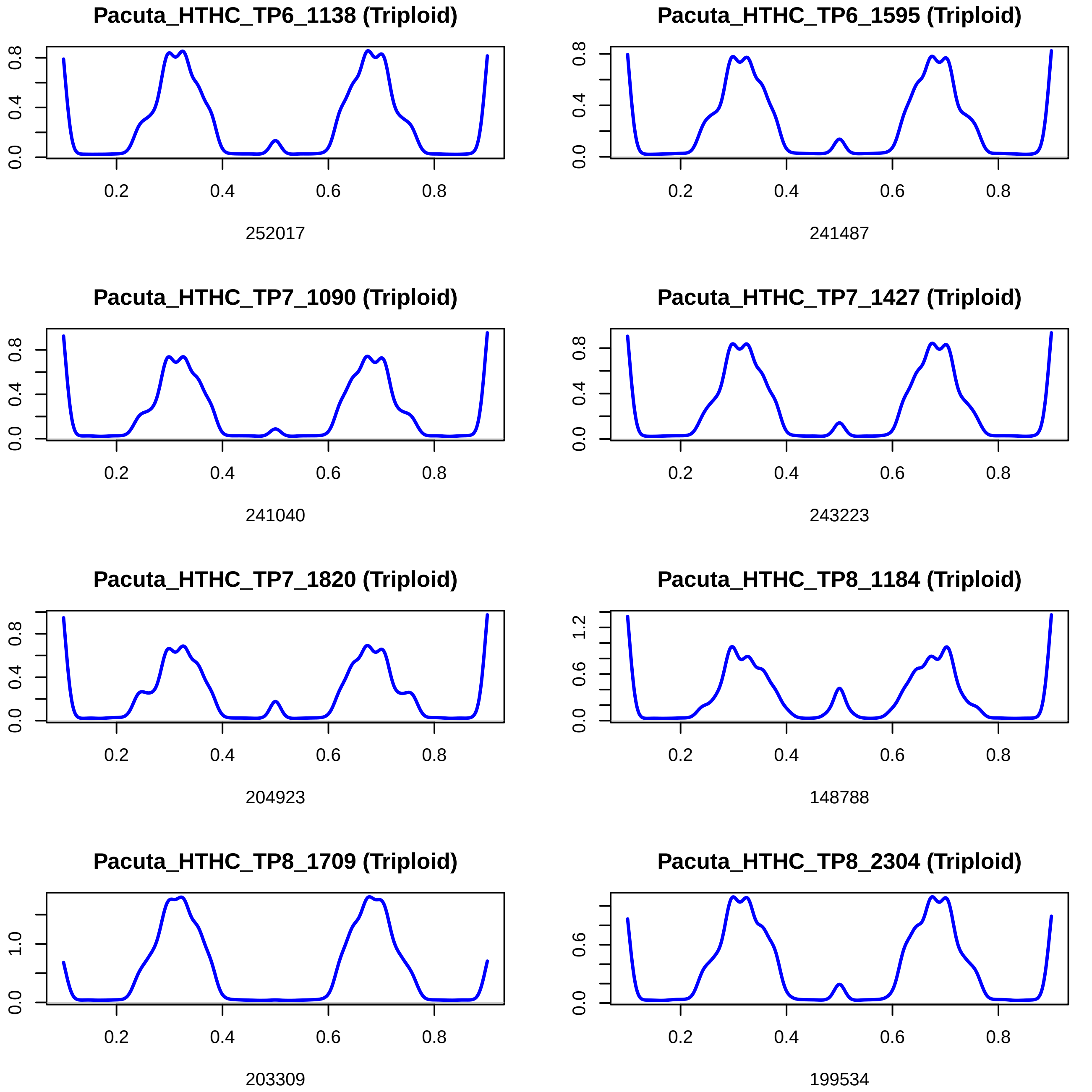
Pacuta_HTHC_TP6_1138 (Triploid)
252017
Pacuta_HTHC_TP6_1595 (Triploid)
241487
Pacuta_HTHC_TP7_1090 (Triploid)
241040
Pacuta_HTHC_TP7_1427 (Triploid)
243223
Pacuta_HTHC_TP7_1820 (Triploid)
204923
Pacuta_HTHC_TP8_1184 (Triploid)
148788
Pacuta_HTHC_TP8_1709 (Triploid)
203309
Pacuta_HTHC_TP8_2304 (Triploid)
199534
0.2
0.4
0.6
0.8
0.0
1.0
1.2

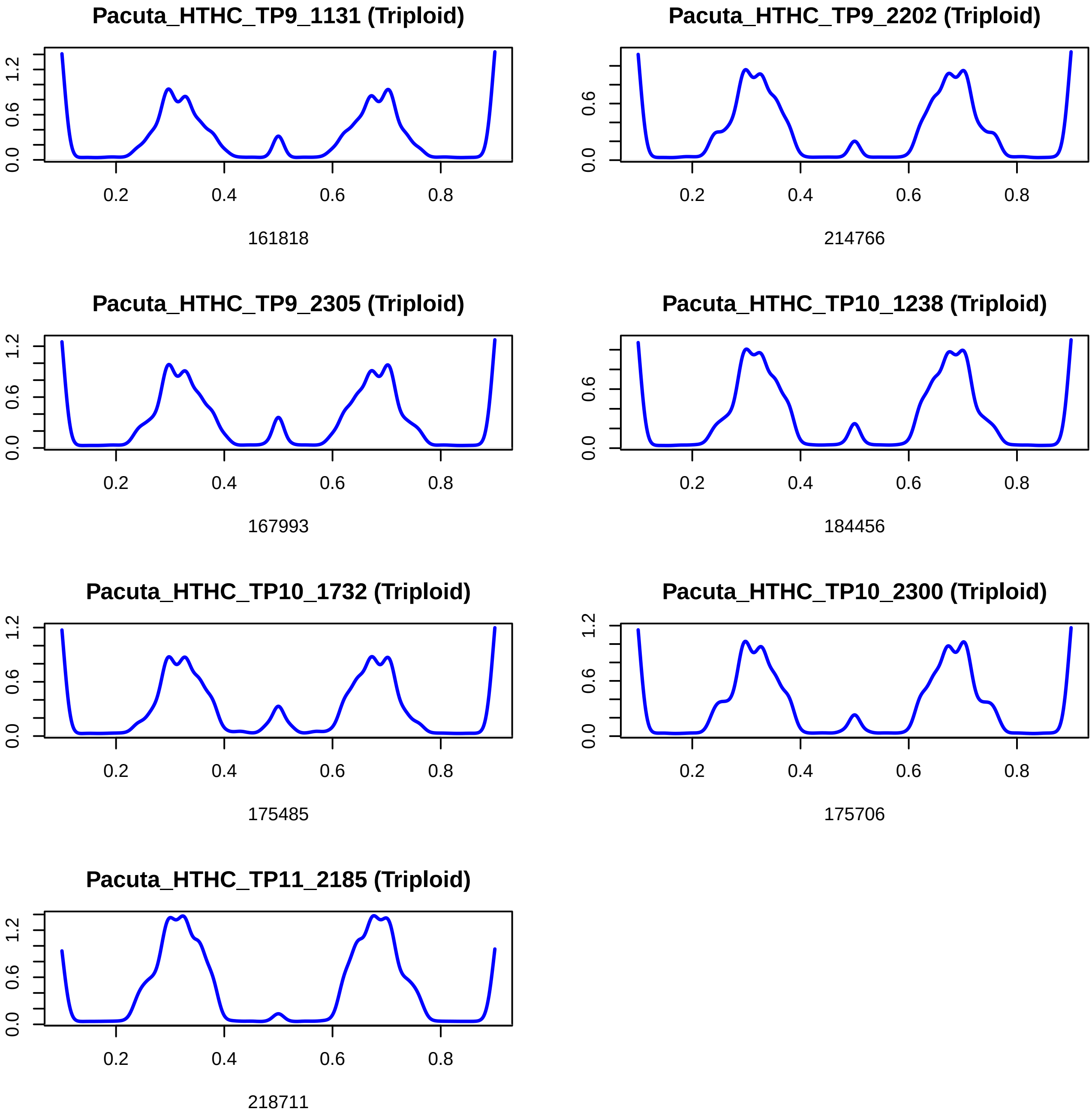
Pacuta_HTHC_TP9_1131 (Triploid)
0.0
0.6
1.2
0.2
0.4
0.6
0.8
161818
Pacuta_HTHC_TP9_2202 (Triploid)
0.0
0.6
0.2
0.4
0.6
0.8
214766
Pacuta_HTHC_TP9_2305 (Triploid)
0.0
0.6
1.2
0.2
0.4
0.6
0.8
167993
Pacuta_HTHC_TP10_1238 (Triploid)
0.0
0.6
0.2
0.4
0.6
0.8
184456
Pacuta_HTHC_TP10_1732 (Triploid)
0.0
0.6
1.2
0.2
0.4
0.6
0.8
175485
Pacuta_HTHC_TP10_2300 (Triploid)
0.0
0.6
1.2
0.2
0.4
0.6
0.8
175706
Pacuta_HTHC_TP11_2185 (Triploid)
0.0
0.6
1.2
0.2
0.4
0.6
0.8
218711
