# Supplementary material for: Ploidy Variation and Its Implications for Reproduction and Population Dynamics in Two Sympatric Hawaiian Coral Species
Source: Genome Biol Evol. 2023 Aug 11;15(8):evad149. doi: 10.1093/gbe/evad149 (PMC10445776; doi:10.1093/gbe/evad149)
Supplement: evad149_Supplementary_Data [file evad149_supplementary_data.zip › Data_S2.pdf]

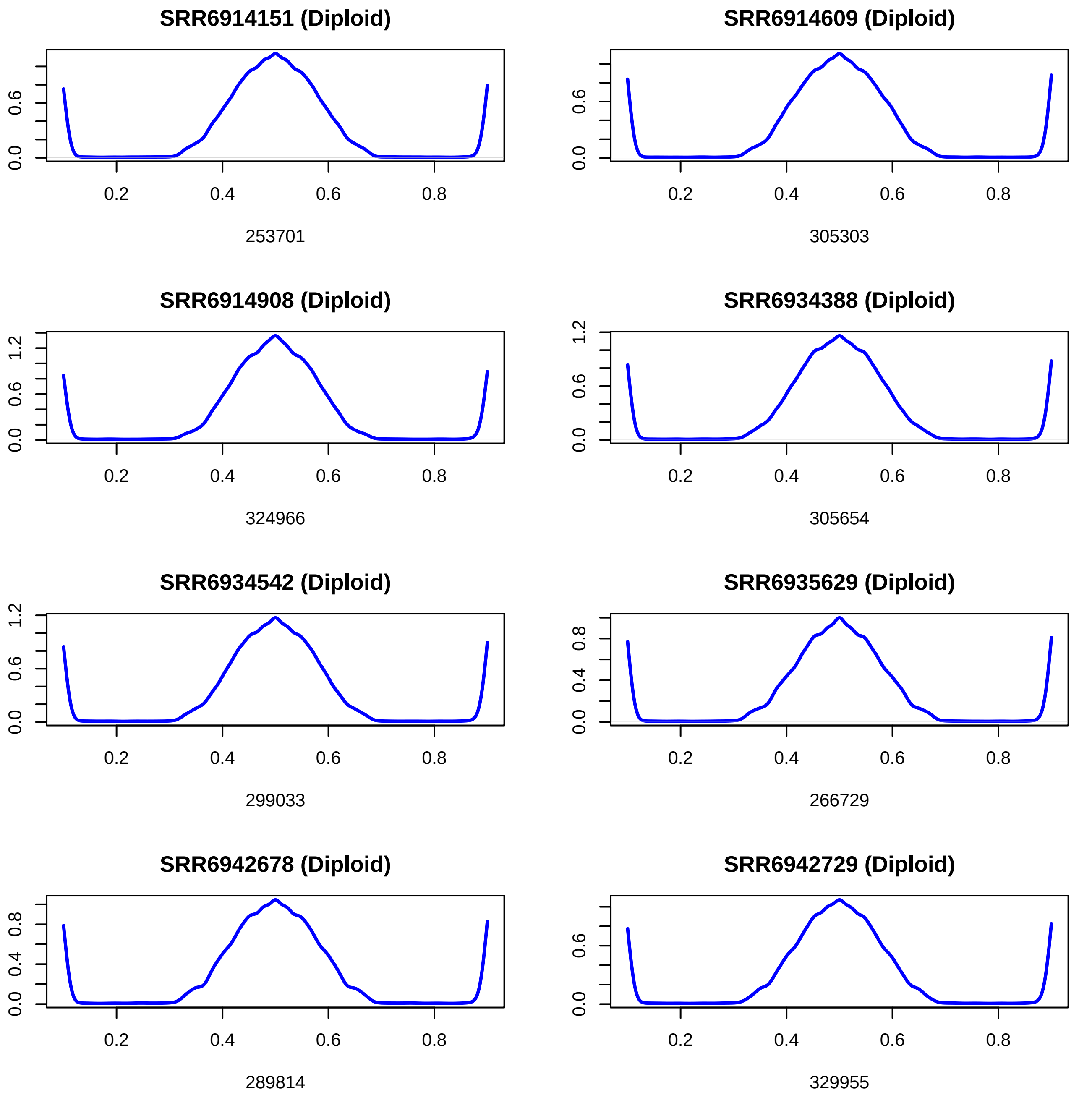
SRR6914151 (Diploid)
0.0
0.6
0.2
0.4
0.6
0.8
253701
SRR6914609 (Diploid)
0.0
0.6
0.2
0.4
0.6
0.8
305303
SRR6914908 (Diploid)
0.0
0.6
1.2
0.2
0.4
0.6
0.8
324966
SRR6934388 (Diploid)
0.0
0.6
1.2
0.2
0.4
0.6
0.8
305654
SRR6934542 (Diploid)
0.0
0.6
1.2
0.2
0.4
0.6
0.8
299033
SRR6935629 (Diploid)
0.0
0.4
0.8
0.2
0.4
0.6
0.8
266729
SRR6942678 (Diploid)
0.0
0.4
0.8
0.2
0.4
0.6
0.8
289814
SRR6942729 (Diploid)
0.0
0.6
0.2
0.4
0.6
0.8
329955

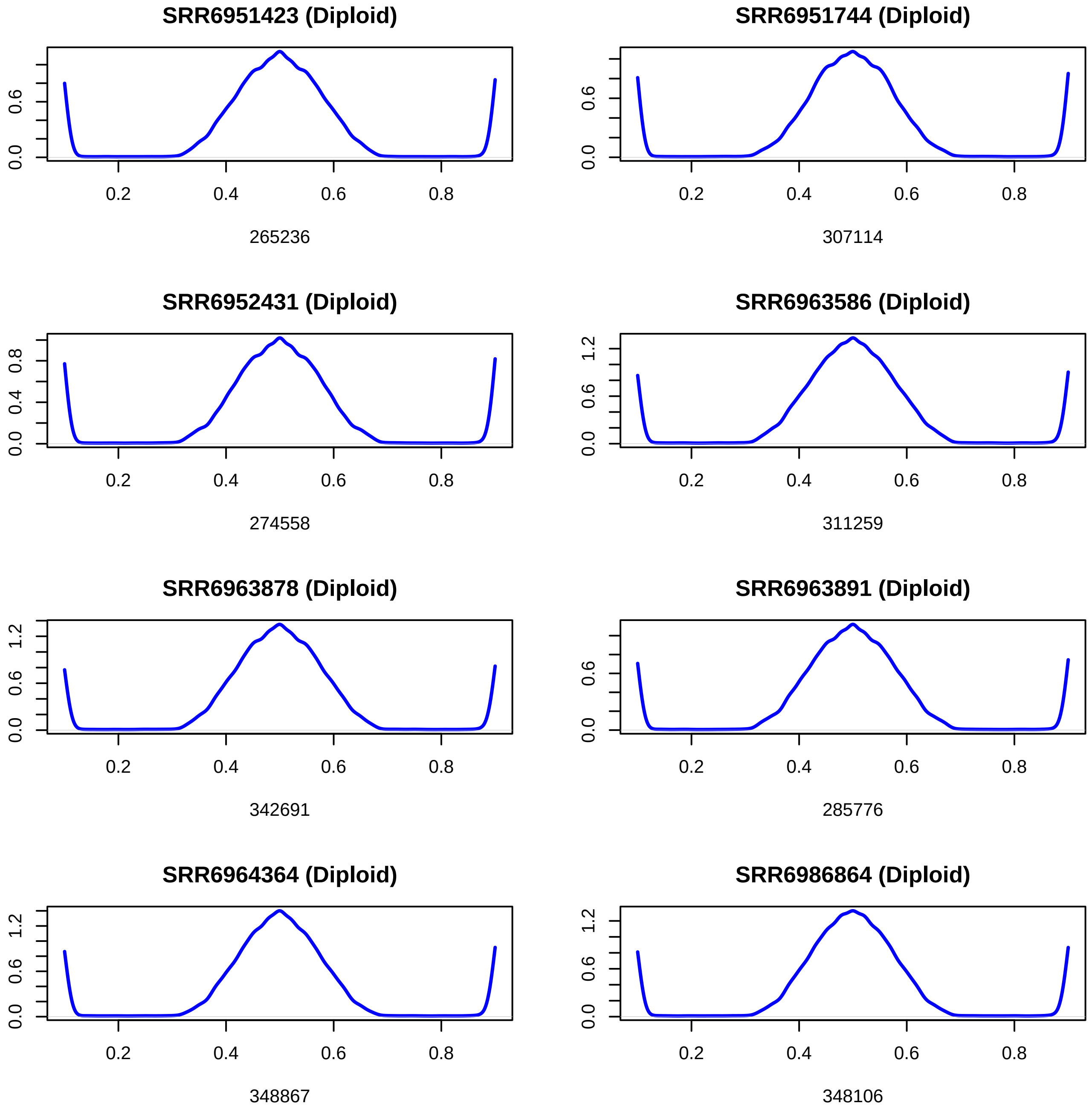
SRR6951423 (Diploid)
0.0
0.6
0.2
0.4
0.6
0.8
265236
SRR6951744 (Diploid)
0.0
0.6
0.2
0.4
0.6
0.8
307114
SRR6952431 (Diploid)
0.0
0.4
0.8
0.2
0.4
0.6
0.8
274558
SRR6963586 (Diploid)
0.0
0.6
1.2
0.2
0.4
0.6
0.8
311259
SRR6963878 (Diploid)
0.0
0.6
1.2
0.2
0.4
0.6
0.8
342691
SRR6963891 (Diploid)
0.0
0.6
0.2
0.4
0.6
0.8
285776
SRR6964364 (Diploid)
0.0
0.6
1.2
0.2
0.4
0.6
0.8
348867
SRR6986864 (Diploid)
0.0
0.6
1.2
0.2
0.4
0.6
0.8
348106

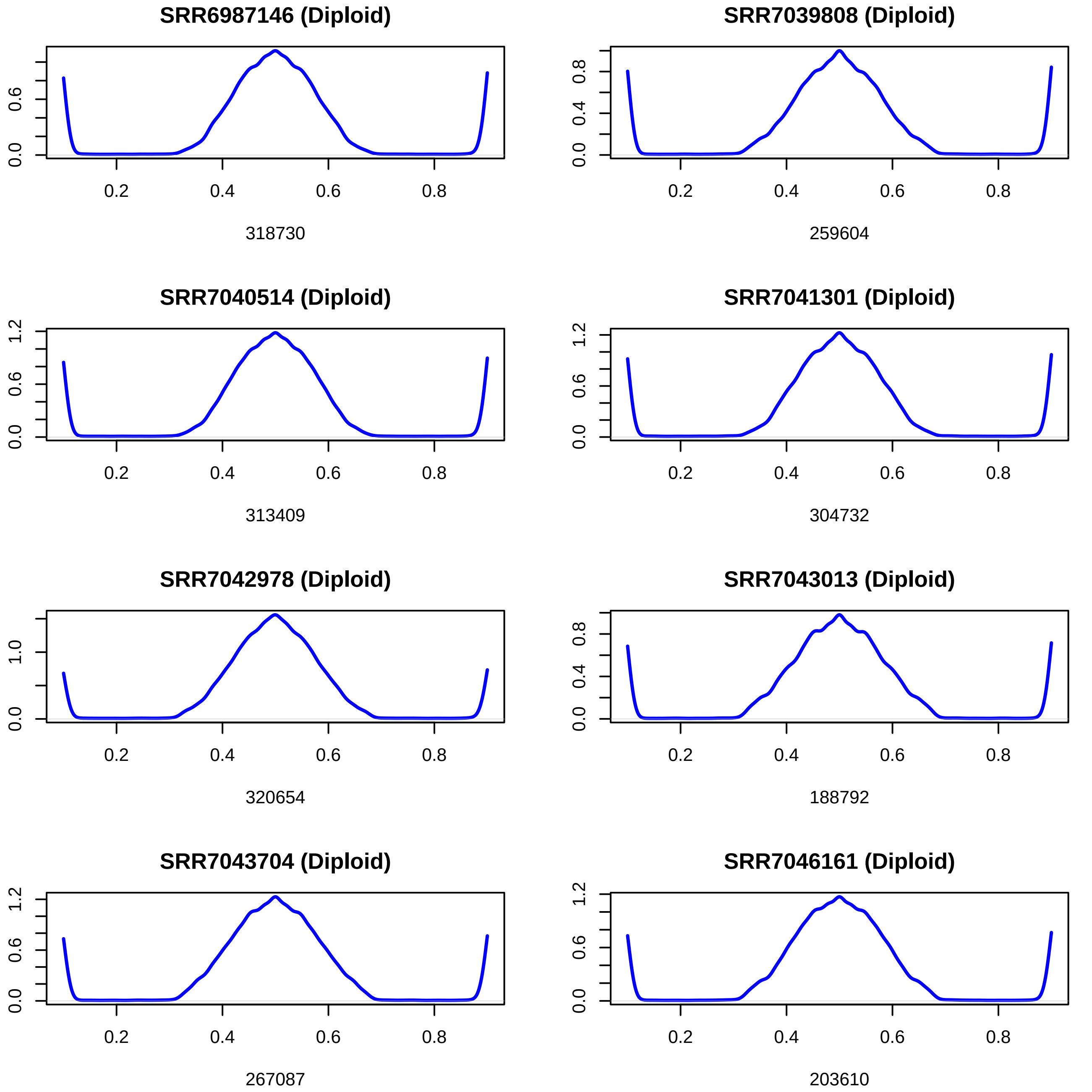
SRR6987146 (Diploid)
0.0
0.6
0.2
0.4
0.6
0.8
318730
SRR7039808 (Diploid)
0.0
0.4
0.8
0.2
0.4
0.6
0.8
259604
SRR7040514 (Diploid)
0.0
0.6
1.2
0.2
0.4
0.6
0.8
313409
SRR7041301 (Diploid)
0.0
0.6
1.2
0.2
0.4
0.6
0.8
304732
SRR7042978 (Diploid)
0.0
1.0
0.2
0.4
0.6
0.8
320654
SRR7043013 (Diploid)
0.0
0.4
0.8
0.2
0.4
0.6
0.8
188792
SRR7043704 (Diploid)
0.0
0.6
1.2
0.2
0.4
0.6
0.8
267087
SRR7046161 (Diploid)
0.0
0.6
1.2
0.2
0.4
0.6
0.8
203610

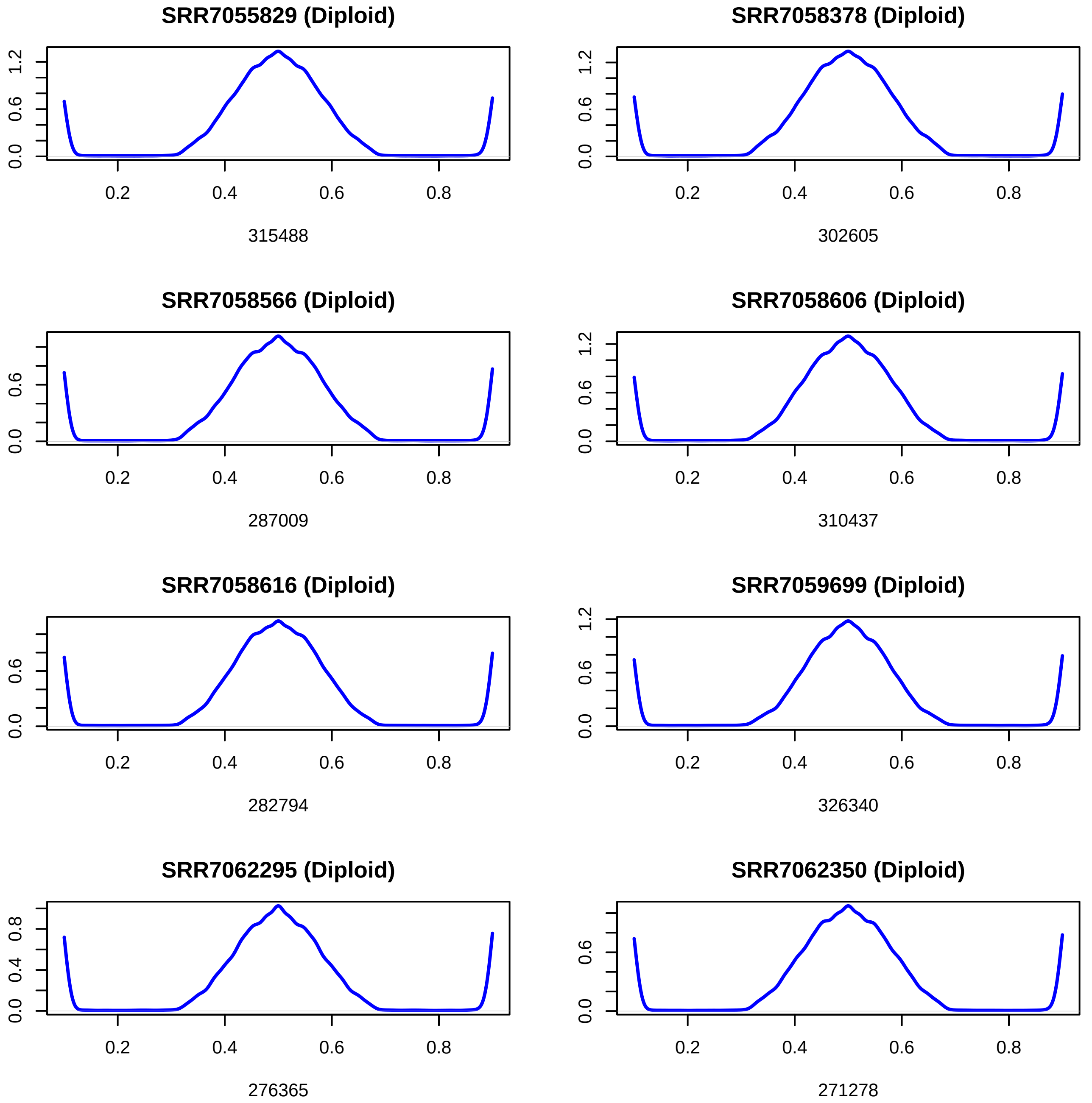
SRR7055829 (Diploid)
315488
SRR7058378 (Diploid)
302605
SRR7058566 (Diploid)
287009
SRR7058606 (Diploid)
310437
SRR7058616 (Diploid)
282794
SRR7059699 (Diploid)
326340
SRR7062295 (Diploid)
276365
SRR7062350 (Diploid)
271278
0.0
0.4
0.6
0.8
1.2
0.2
0.4
0.6
0.8
