# Supplementary material for: Ploidy Variation and Its Implications for Reproduction and Population Dynamics in Two Sympatric Hawaiian Coral Species
Source: Genome Biol Evol. 2023 Aug 11;15(8):evad149. doi: 10.1093/gbe/evad149 (PMC10445776; doi:10.1093/gbe/evad149)
Supplement: evad149_Supplementary_Data [file evad149_supplementary_data.zip › Data_S3.pdf]

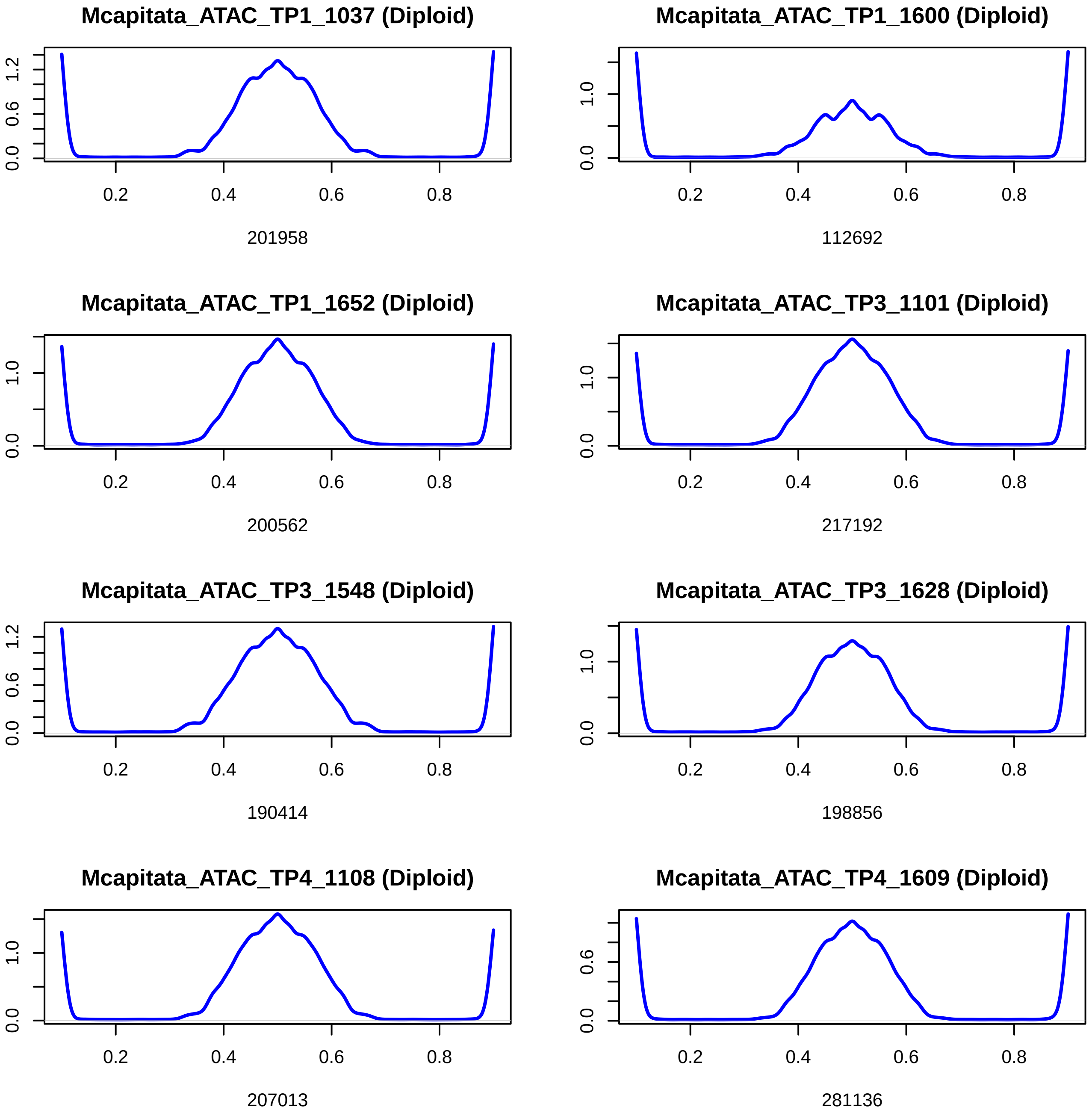
Mcapitata_ATAC_TP1_1037 (Diploid)
201958
Mcapitata_ATAC_TP1_1600 (Diploid)
112692
Mcapitata_ATAC_TP1_1652 (Diploid)
200562
Mcapitata_ATAC_TP3_1101 (Diploid)
217192
Mcapitata_ATAC_TP3_1548 (Diploid)
190414
Mcapitata_ATAC_TP3_1628 (Diploid)
198856
Mcapitata_ATAC_TP4_1108 (Diploid)
207013
Mcapitata_ATAC_TP4_1609 (Diploid)
281136
0.2
0.4
0.6
0.8
0.0
0.6
1.2
1.0

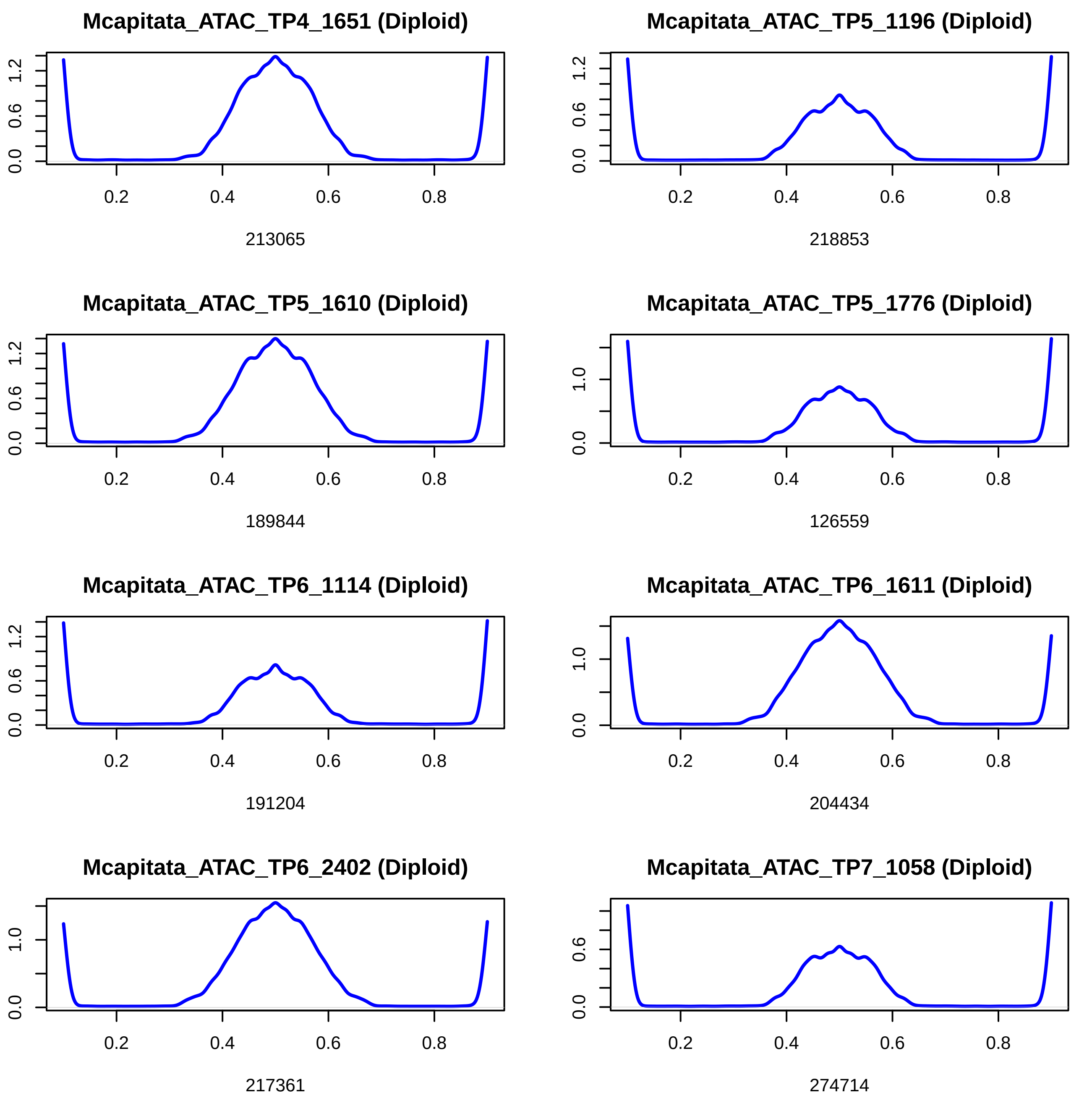
Mcapitata_ATAC_TP4_1651 (Diploid)
0.0
0.6
1.2
0.2
0.4
0.6
0.8
213065
Mcapitata_ATAC_TP5_1196 (Diploid)
0.0
0.6
1.2
0.2
0.4
0.6
0.8
218853
Mcapitata_ATAC_TP5_1610 (Diploid)
0.0
0.6
1.2
0.2
0.4
0.6
0.8
189844
Mcapitata_ATAC_TP5_1776 (Diploid)
0.0
1.0
0.2
0.4
0.6
0.8
126559
Mcapitata_ATAC_TP6_1114 (Diploid)
0.0
0.6
1.2
0.2
0.4
0.6
0.8
191204
Mcapitata_ATAC_TP6_1611 (Diploid)
0.0
1.0
0.2
0.4
0.6
0.8
204434
Mcapitata_ATAC_TP6_2402 (Diploid)
0.0
1.0
0.2
0.4
0.6
0.8
217361
Mcapitata_ATAC_TP7_1058 (Diploid)
0.0
0.6
0.2
0.4
0.6
0.8
274714

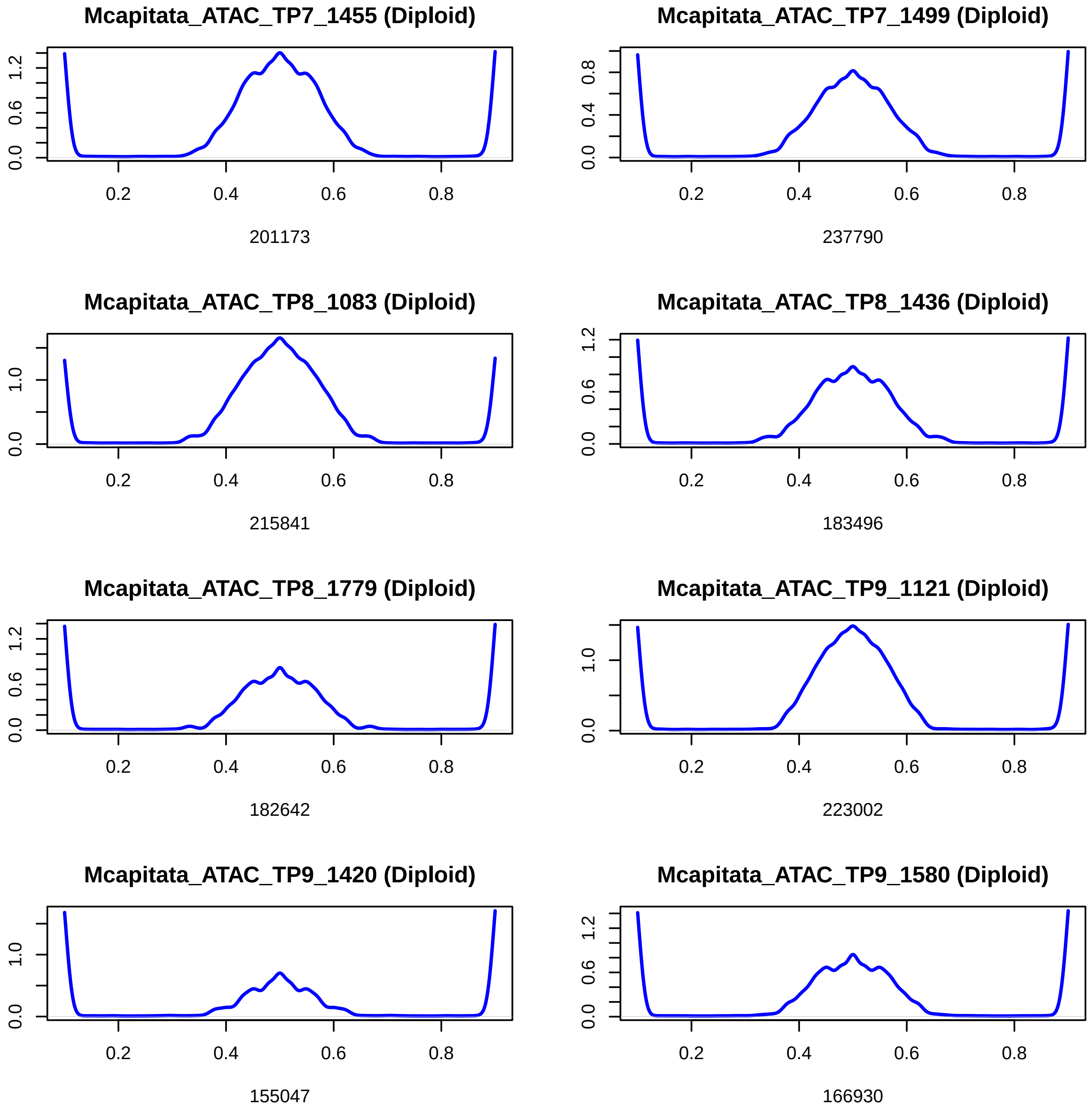
Mcapitata_ATAC_TP7_1455 (Diploid)
0.0
0.6
1.2
0.2
0.4
0.6
0.8
201173
Mcapitata_ATAC_TP7_1499 (Diploid)
0.0
0.4
0.8
0.2
0.4
0.6
0.8
237790
Mcapitata_ATAC_TP8_1083 (Diploid)
0.0
1.0
0.2
0.4
0.6
0.8
215841
Mcapitata_ATAC_TP8_1436 (Diploid)
0.0
0.6
1.2
0.2
0.4
0.6
0.8
183496
Mcapitata_ATAC_TP8_1779 (Diploid)
0.0
0.6
1.2
0.2
0.4
0.6
0.8
182642
Mcapitata_ATAC_TP9_1121 (Diploid)
0.0
1.0
0.2
0.4
0.6
0.8
223002
Mcapitata_ATAC_TP9_1420 (Diploid)
0.0
1.0
0.2
0.4
0.6
0.8
155047
Mcapitata_ATAC_TP9_1580 (Diploid)
0.0
0.6
1.2
0.2
0.4
0.6
0.8
166930

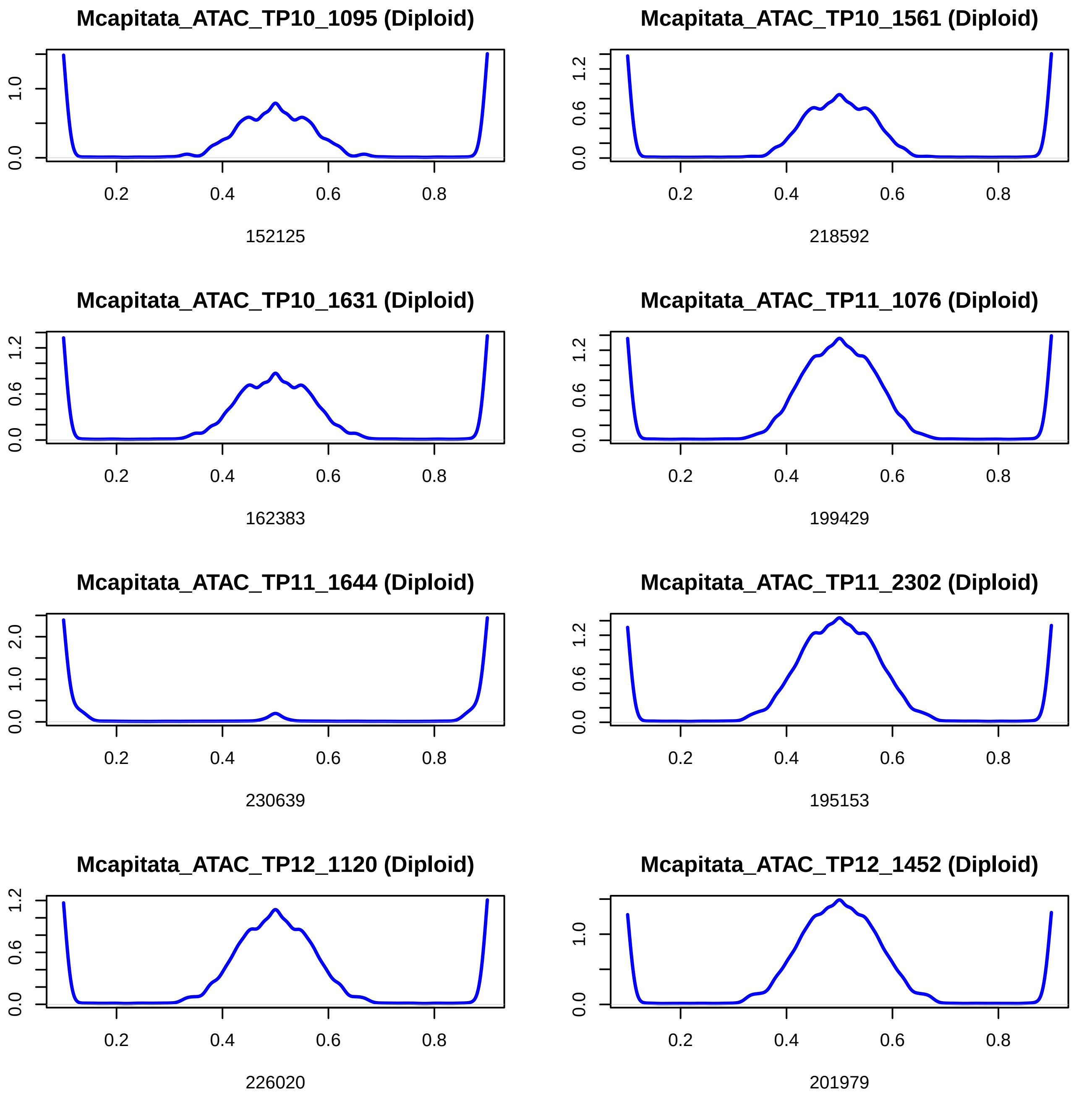
Mcapitata_ATAC_TP10_1095 (Diploid)
152125
Mcapitata_ATAC_TP10_1561 (Diploid)
218592
Mcapitata_ATAC_TP10_1631 (Diploid)
162383
Mcapitata_ATAC_TP11_1076 (Diploid)
199429
Mcapitata_ATAC_TP11_1644 (Diploid)
230639
Mcapitata_ATAC_TP11_2302 (Diploid)
195153
Mcapitata_ATAC_TP12_1120 (Diploid)
226020
Mcapitata_ATAC_TP12_1452 (Diploid)
201979

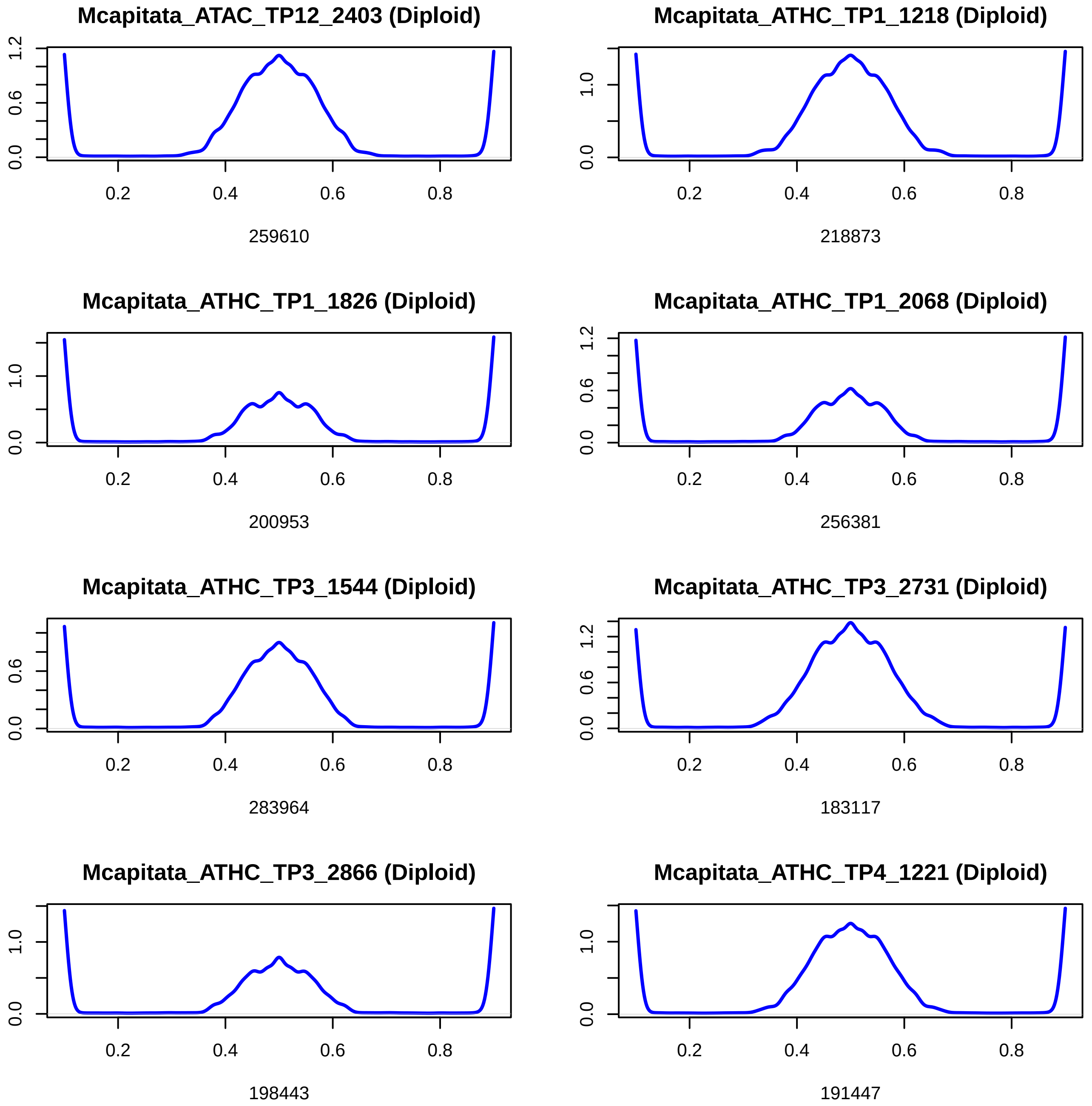
Mcapitata_ATAC_TP12_2403 (Diploid)
0.0
0.6
1.2
0.2
0.4
0.6
0.8
259610
Mcapitata_ATHC_TP1_1218 (Diploid)
0.0
1.0
0.2
0.4
0.6
0.8
218873
Mcapitata_ATHC_TP1_1826 (Diploid)
0.0
1.0
0.2
0.4
0.6
0.8
200953
Mcapitata_ATHC_TP1_2068 (Diploid)
0.0
0.6
1.2
0.2
0.4
0.6
0.8
256381
Mcapitata_ATHC_TP3_1544 (Diploid)
0.0
0.6
0.2
0.4
0.6
0.8
283964
Mcapitata_ATHC_TP3_2731 (Diploid)
0.0
0.6
1.2
0.2
0.4
0.6
0.8
183117
Mcapitata_ATHC_TP3_2866 (Diploid)
0.0
1.0
0.2
0.4
0.6
0.8
198443
Mcapitata_ATHC_TP4_1221 (Diploid)
0.0
1.0
0.2
0.4
0.6
0.8
191447

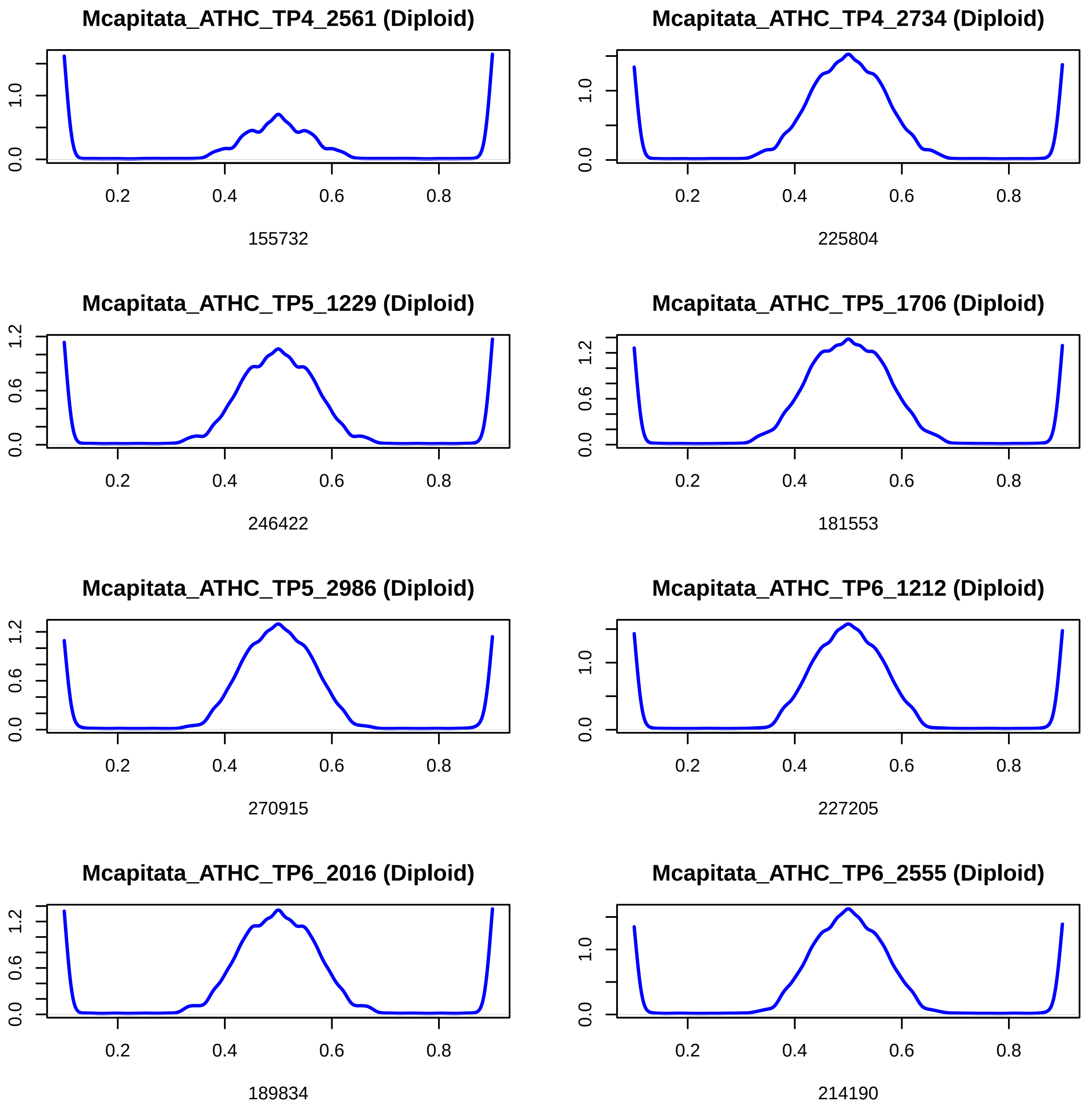
Mcapitata_ATHC_TP4_2561 (Diploid)
0.0
1.0
0.2
0.4
0.6
0.8
155732
Mcapitata_ATHC_TP4_2734 (Diploid)
0.0
1.0
0.2
0.4
0.6
0.8
225804
Mcapitata_ATHC_TP5_1229 (Diploid)
0.0
0.6
1.2
0.2
0.4
0.6
0.8
246422
Mcapitata_ATHC_TP5_1706 (Diploid)
0.0
0.6
1.2
0.2
0.4
0.6
0.8
181553
Mcapitata_ATHC_TP5_2986 (Diploid)
0.0
0.6
1.2
0.2
0.4
0.6
0.8
270915
Mcapitata_ATHC_TP6_1212 (Diploid)
0.0
1.0
0.2
0.4
0.6
0.8
227205
Mcapitata_ATHC_TP6_2016 (Diploid)
0.0
0.6
1.2
0.2
0.4
0.6
0.8
189834
Mcapitata_ATHC_TP6_2555 (Diploid)
0.0
1.0
0.2
0.4
0.6
0.8
214190

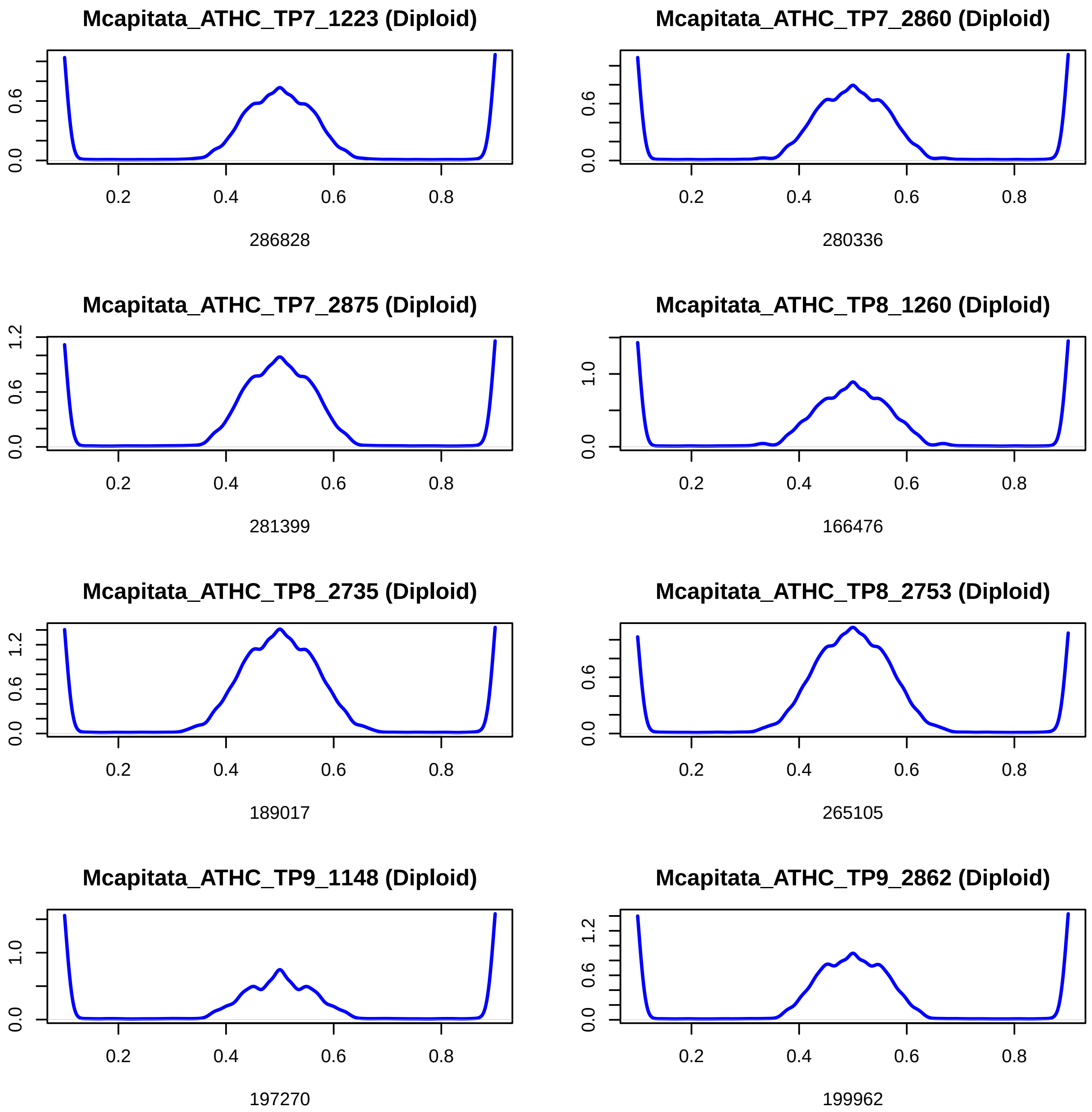
Mcapitata_ATHC_TP7_1223 (Diploid)
0.0
0.6
0.2
0.4
0.6
0.8
286828
Mcapitata_ATHC_TP7_2860 (Diploid)
0.0
0.6
0.2
0.4
0.6
0.8
280336
Mcapitata_ATHC_TP7_2875 (Diploid)
0.0
0.6
1.2
0.2
0.4
0.6
0.8
281399
Mcapitata_ATHC_TP8_1260 (Diploid)
0.0
1.0
0.2
0.4
0.6
0.8
166476
Mcapitata_ATHC_TP8_2735 (Diploid)
0.0
0.6
1.2
0.2
0.4
0.6
0.8
189017
Mcapitata_ATHC_TP8_2753 (Diploid)
0.0
0.6
0.2
0.4
0.6
0.8
265105
Mcapitata_ATHC_TP9_1148 (Diploid)
0.0
1.0
0.2
0.4
0.6
0.8
197270
Mcapitata_ATHC_TP9_2862 (Diploid)
0.0
0.6
1.2
0.2
0.4
0.6
0.8
199962

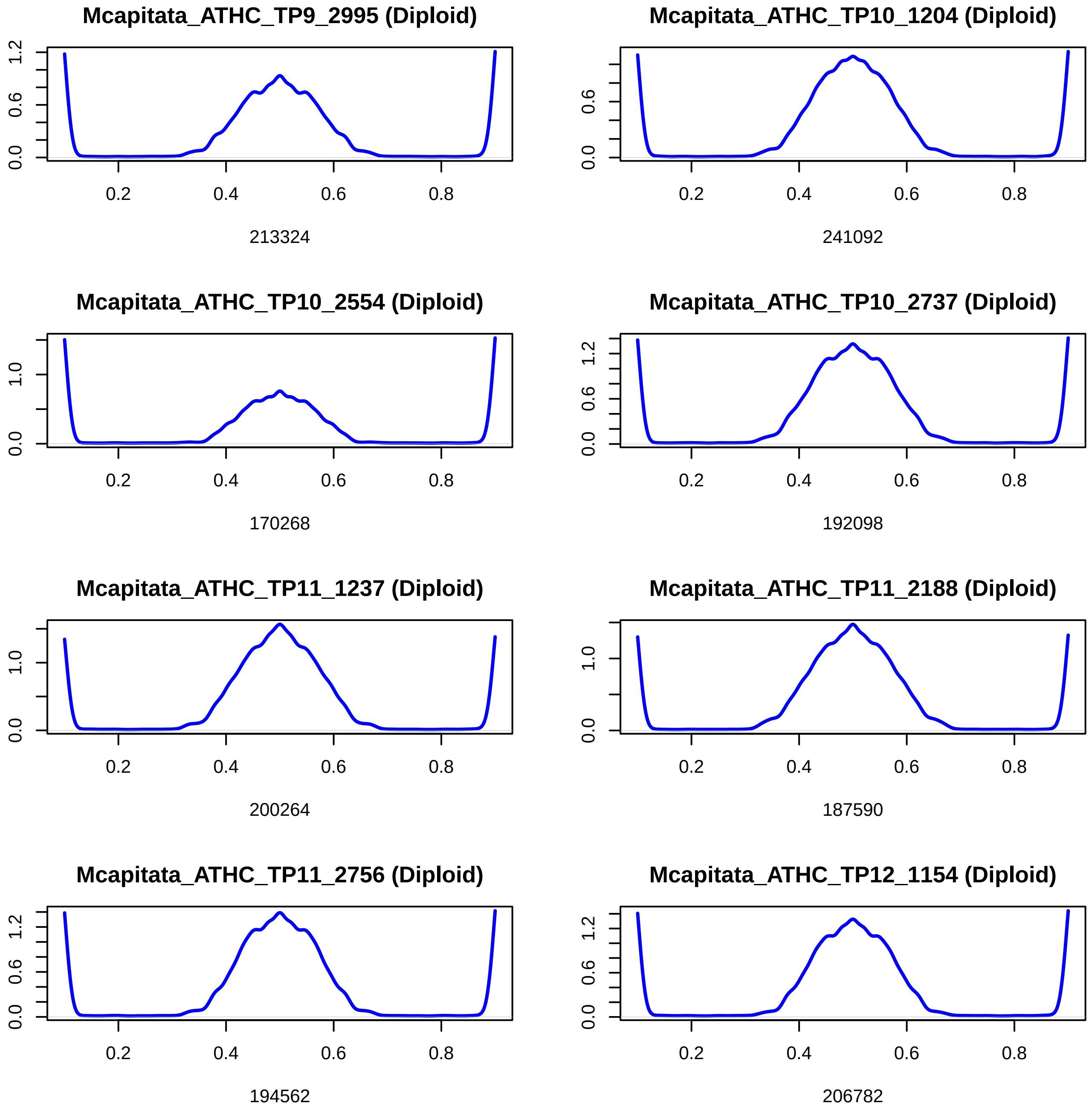

Mcapitata_ATHC_TP9_2995 (Diploid)
213324
Mcapitata_ATHC_TP10_1204 (Diploid)
241092
Mcapitata_ATHC_TP10_2554 (Diploid)
170268
Mcapitata_ATHC_TP10_2737 (Diploid)
192098
Mcapitata_ATHC_TP11_1237 (Diploid)
200264
Mcapitata_ATHC_TP11_2188 (Diploid)
187590
Mcapitata_ATHC_TP11_2756 (Diploid)
194562
Mcapitata_ATHC_TP12_1154 (Diploid)
206782
0.2
0.4
0.6
0.8
0.0
0.6
1.0
1.2

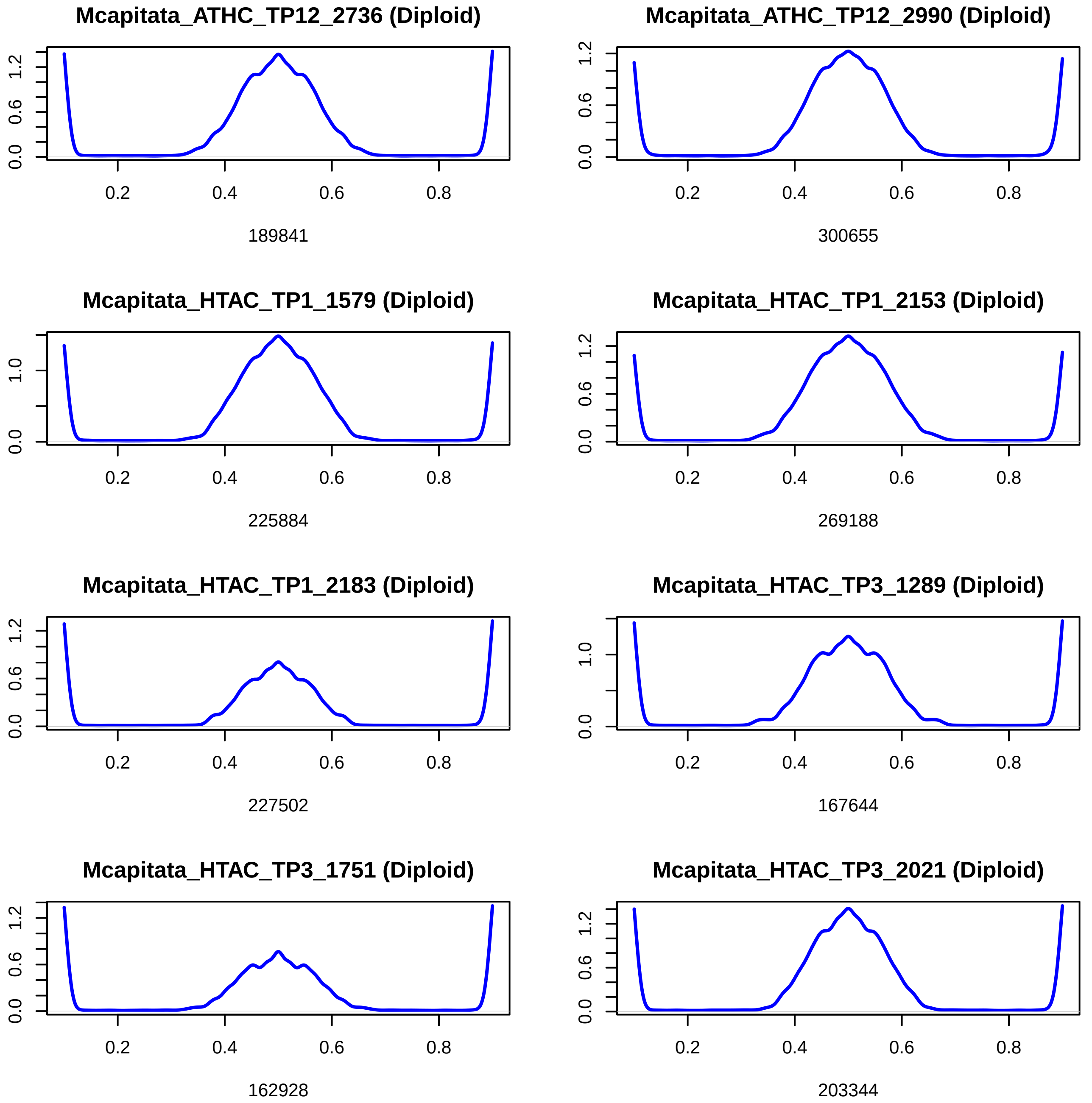

Mcapitata_ATHC_TP12_2736 (Diploid)
0.0
0.6
1.2
0.2
0.4
0.6
0.8
189841
Mcapitata_ATHC_TP12_2990 (Diploid)
0.0
0.6
1.2
0.2
0.4
0.6
0.8
300655
Mcapitata_HTAC_TP1_1579 (Diploid)
0.0
1.0
0.2
0.4
0.6
0.8
225884
Mcapitata_HTAC_TP1_2153 (Diploid)
0.0
0.6
1.2
0.2
0.4
0.6
0.8
269188
Mcapitata_HTAC_TP1_2183 (Diploid)
0.0
0.6
1.2
0.2
0.4
0.6
0.8
227502
Mcapitata_HTAC_TP3_1289 (Diploid)
0.0
1.0
0.2
0.4
0.6
0.8
167644
Mcapitata_HTAC_TP3_1751 (Diploid)
0.0
0.6
1.2
0.2
0.4
0.6
0.8
162928
Mcapitata_HTAC_TP3_2021 (Diploid)
0.0
0.6
1.2
0.2
0.4
0.6
0.8
203344

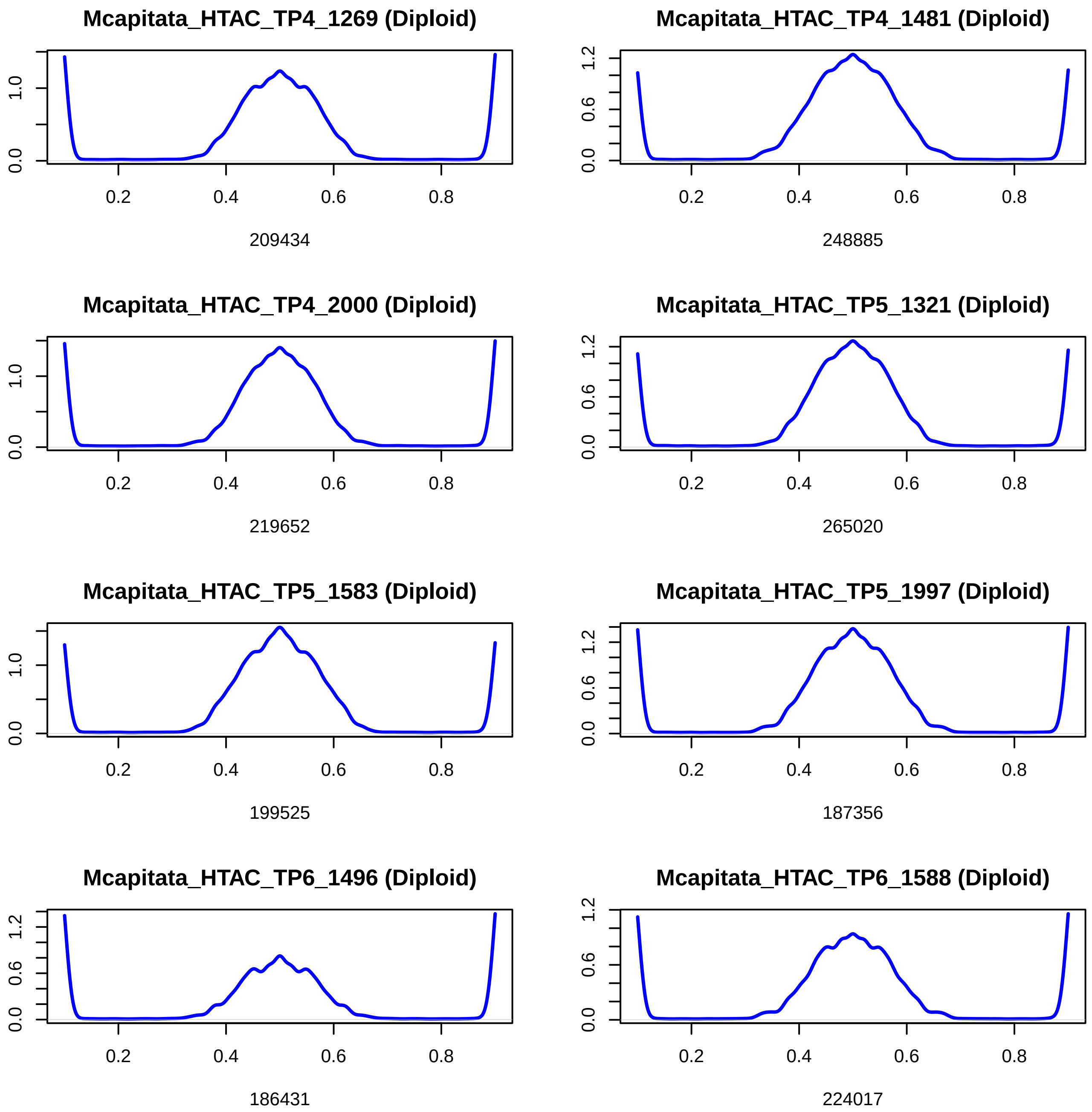
Mcapitata_HTAC_TP4_1269 (Diploid)
209434
Mcapitata_HTAC_TP4_1481 (Diploid)
248885
Mcapitata_HTAC_TP4_2000 (Diploid)
219652
Mcapitata_HTAC_TP5_1321 (Diploid)
265020
Mcapitata_HTAC_TP5_1583 (Diploid)
199525
Mcapitata_HTAC_TP5_1997 (Diploid)
187356
Mcapitata_HTAC_TP6_1496 (Diploid)
186431
Mcapitata_HTAC_TP6_1588 (Diploid)
224017
0.2
0.4
0.6
0.8
0.0
0.6
1.0
1.2

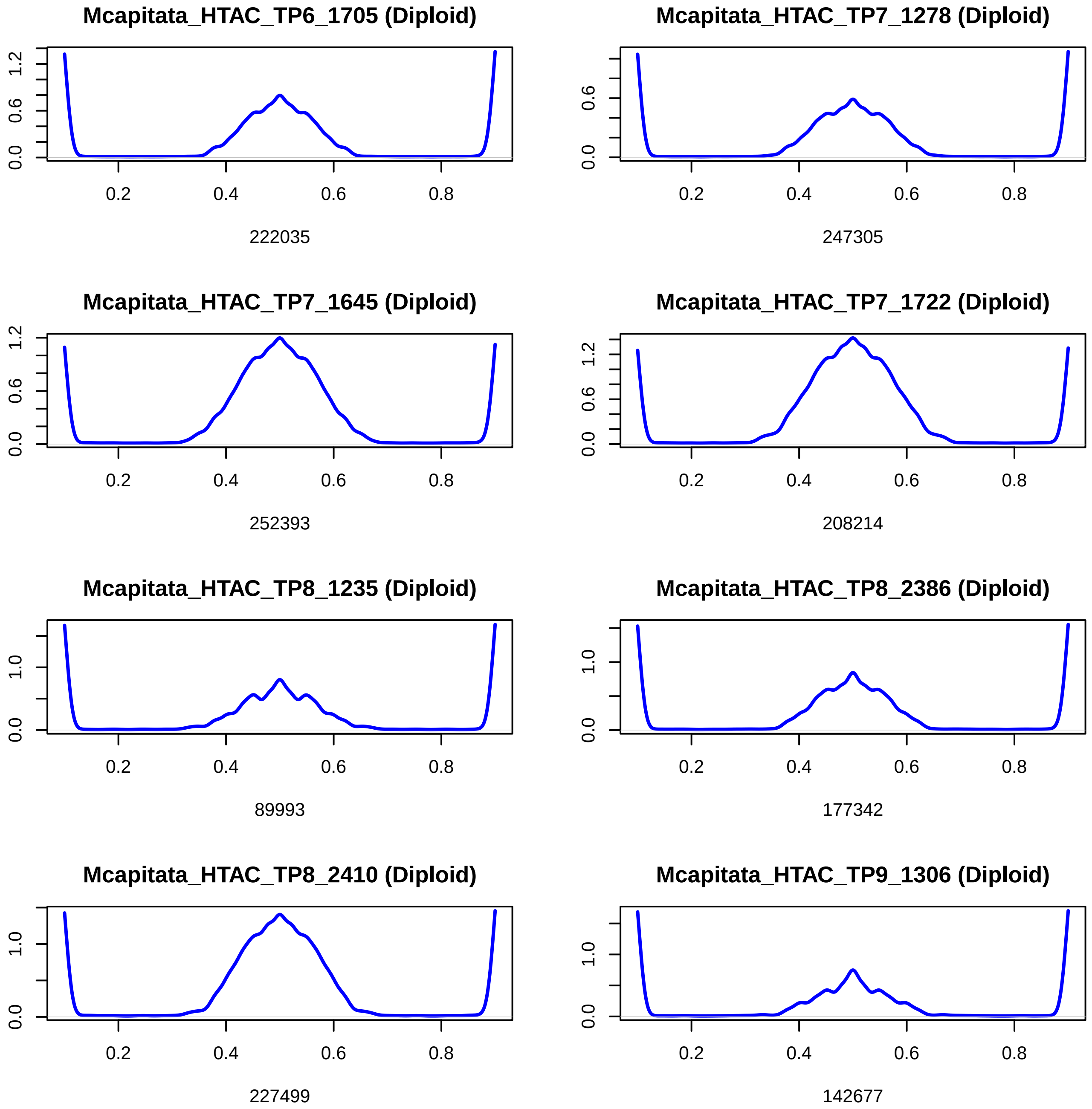
Mcapitata_HTAC_TP6_1705 (Diploid)
222035
Mcapitata_HTAC_TP7_1278 (Diploid)
247305
Mcapitata_HTAC_TP7_1645 (Diploid)
252393
Mcapitata_HTAC_TP7_1722 (Diploid)
208214
Mcapitata_HTAC_TP8_1235 (Diploid)
89993
Mcapitata_HTAC_TP8_2386 (Diploid)
177342
Mcapitata_HTAC_TP8_2410 (Diploid)
227499
Mcapitata_HTAC_TP9_1306 (Diploid)
142677

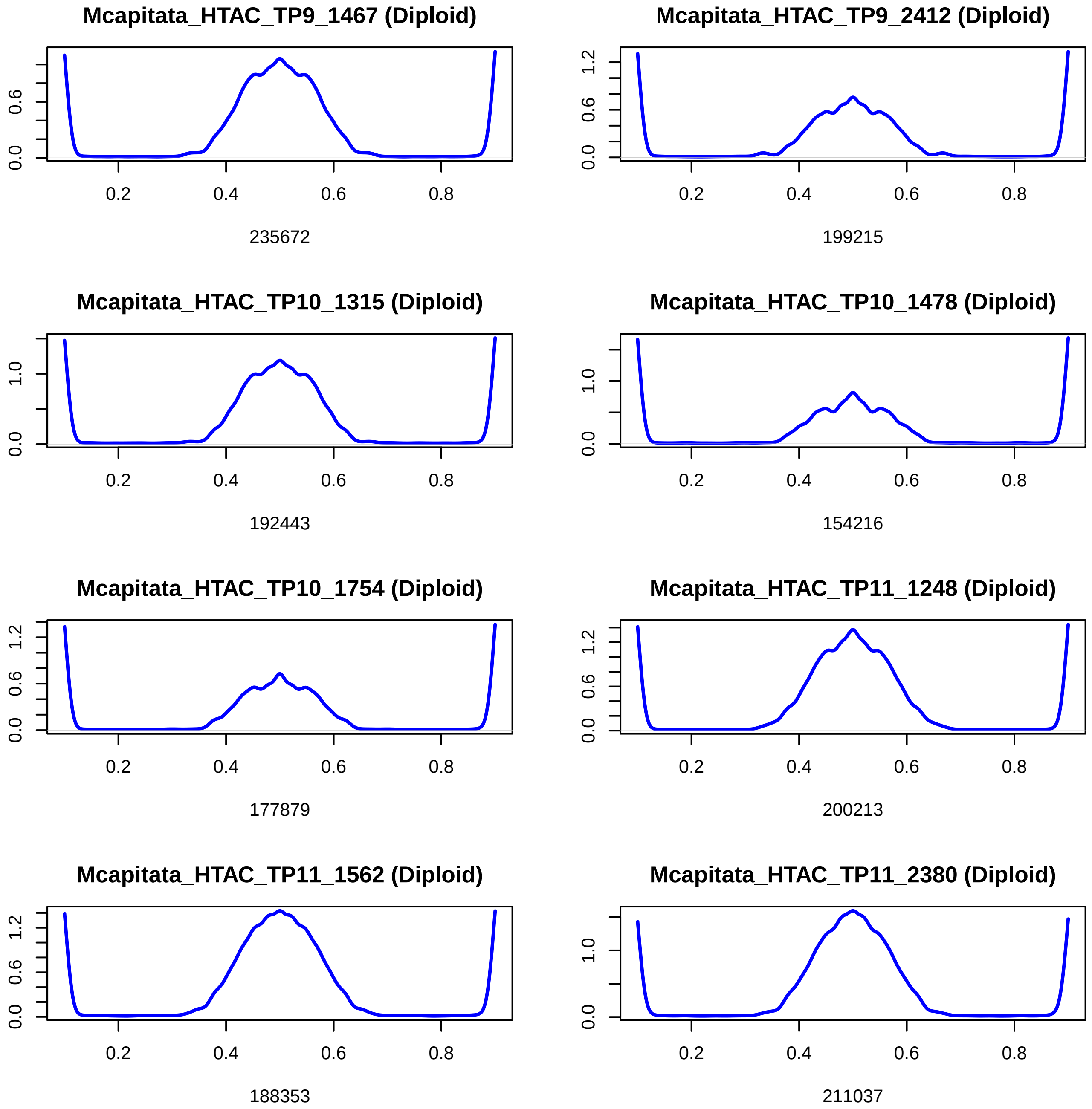
Mcapitata_HTAC_TP9_1467 (Diploid)
235672
Mcapitata_HTAC_TP9_2412 (Diploid)
199215
Mcapitata_HTAC_TP10_1315 (Diploid)
192443
Mcapitata_HTAC_TP10_1478 (Diploid)
154216
Mcapitata_HTAC_TP10_1754 (Diploid)
177879
Mcapitata_HTAC_TP11_1248 (Diploid)
200213
Mcapitata_HTAC_TP11_1562 (Diploid)
188353
Mcapitata_HTAC_TP11_2380 (Diploid)
211037
0.0
0.6
1.2
1.0
0.2
0.4
0.6
0.8

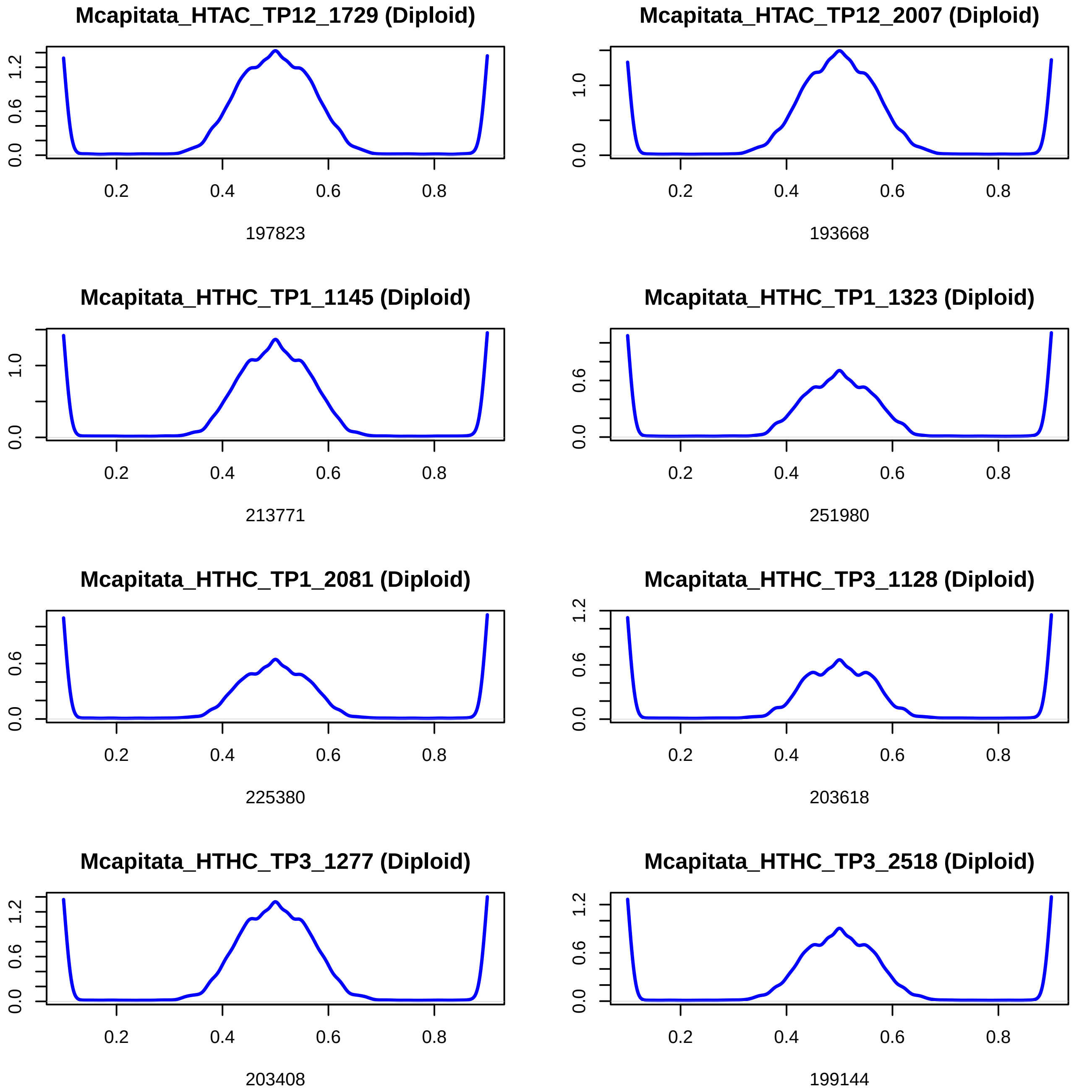
Mcapitata_HTAC_TP12_1729 (Diploid)
0.0
0.6
1.2
0.2
0.4
0.6
0.8
197823
Mcapitata_HTAC_TP12_2007 (Diploid)
0.0
1.0
0.2
0.4
0.6
0.8
193668
Mcapitata_HTHC_TP1_1145 (Diploid)
0.0
1.0
0.2
0.4
0.6
0.8
213771
Mcapitata_HTHC_TP1_1323 (Diploid)
0.0
0.6
0.2
0.4
0.6
0.8
251980
Mcapitata_HTHC_TP1_2081 (Diploid)
0.0
0.6
0.2
0.4
0.6
0.8
225380
Mcapitata_HTHC_TP3_1128 (Diploid)
0.0
0.6
1.2
0.2
0.4
0.6
0.8
203618
Mcapitata_HTHC_TP3_1277 (Diploid)
0.0
0.6
1.2
0.2
0.4
0.6
0.8
203408
Mcapitata_HTHC_TP3_2518 (Diploid)
0.0
0.6
1.2
0.2
0.4
0.6
0.8
199144

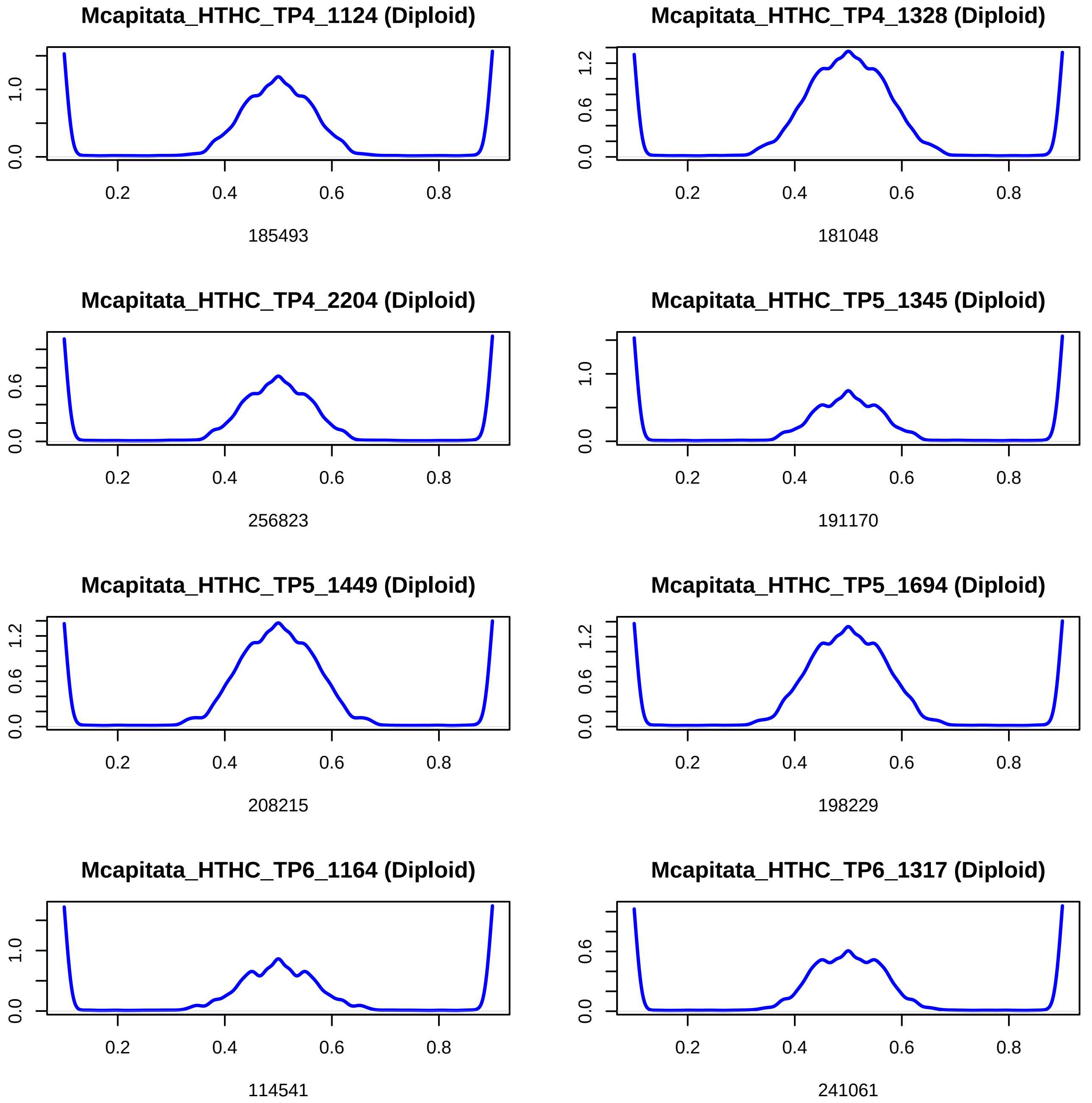

Mcapitata_HTHC_TP4_1124 (Diploid)
185493
Mcapitata_HTHC_TP4_1328 (Diploid)
181048
Mcapitata_HTHC_TP4_2204 (Diploid)
256823
Mcapitata_HTHC_TP5_1345 (Diploid)
191170
Mcapitata_HTHC_TP5_1449 (Diploid)
208215
Mcapitata_HTHC_TP5_1694 (Diploid)
198229
Mcapitata_HTHC_TP6_1164 (Diploid)
114541
Mcapitata_HTHC_TP6_1317 (Diploid)
241061
0.2
0.4
0.6
0.8
0.0
0.6
1.0
1.2

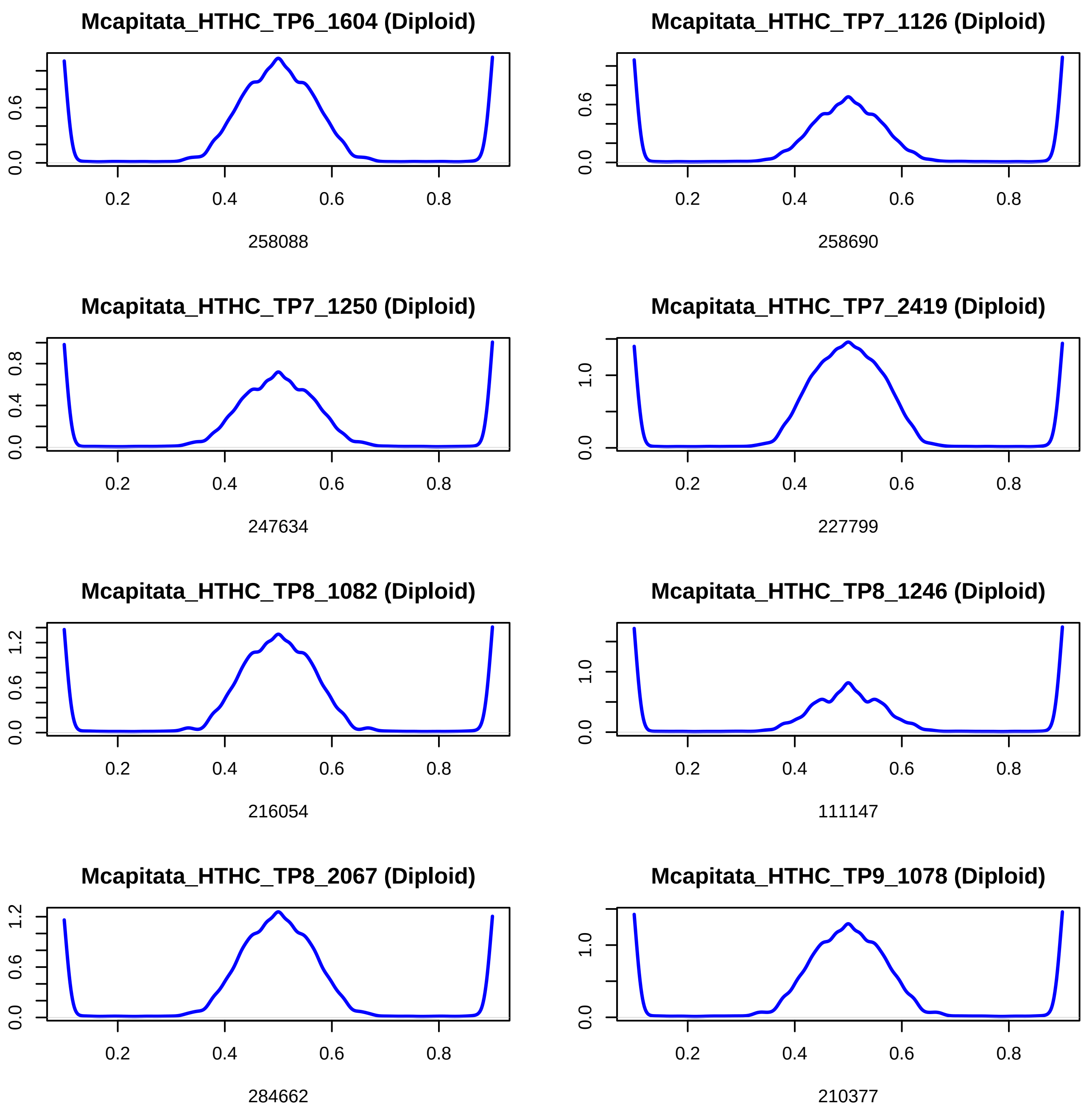
Mcapitata_HTHC_TP6_1604 (Diploid)
258088
Mcapitata_HTHC_TP7_1126 (Diploid)
258690
Mcapitata_HTHC_TP7_1250 (Diploid)
247634
Mcapitata_HTHC_TP7_2419 (Diploid)
227799
Mcapitata_HTHC_TP8_1082 (Diploid)
216054
Mcapitata_HTHC_TP8_1246 (Diploid)
111147
Mcapitata_HTHC_TP8_2067 (Diploid)
284662
Mcapitata_HTHC_TP9_1078 (Diploid)
210377
0.2
0.4
0.6
0.8
0.0
0.4
0.6
0.8
1.0
1.2

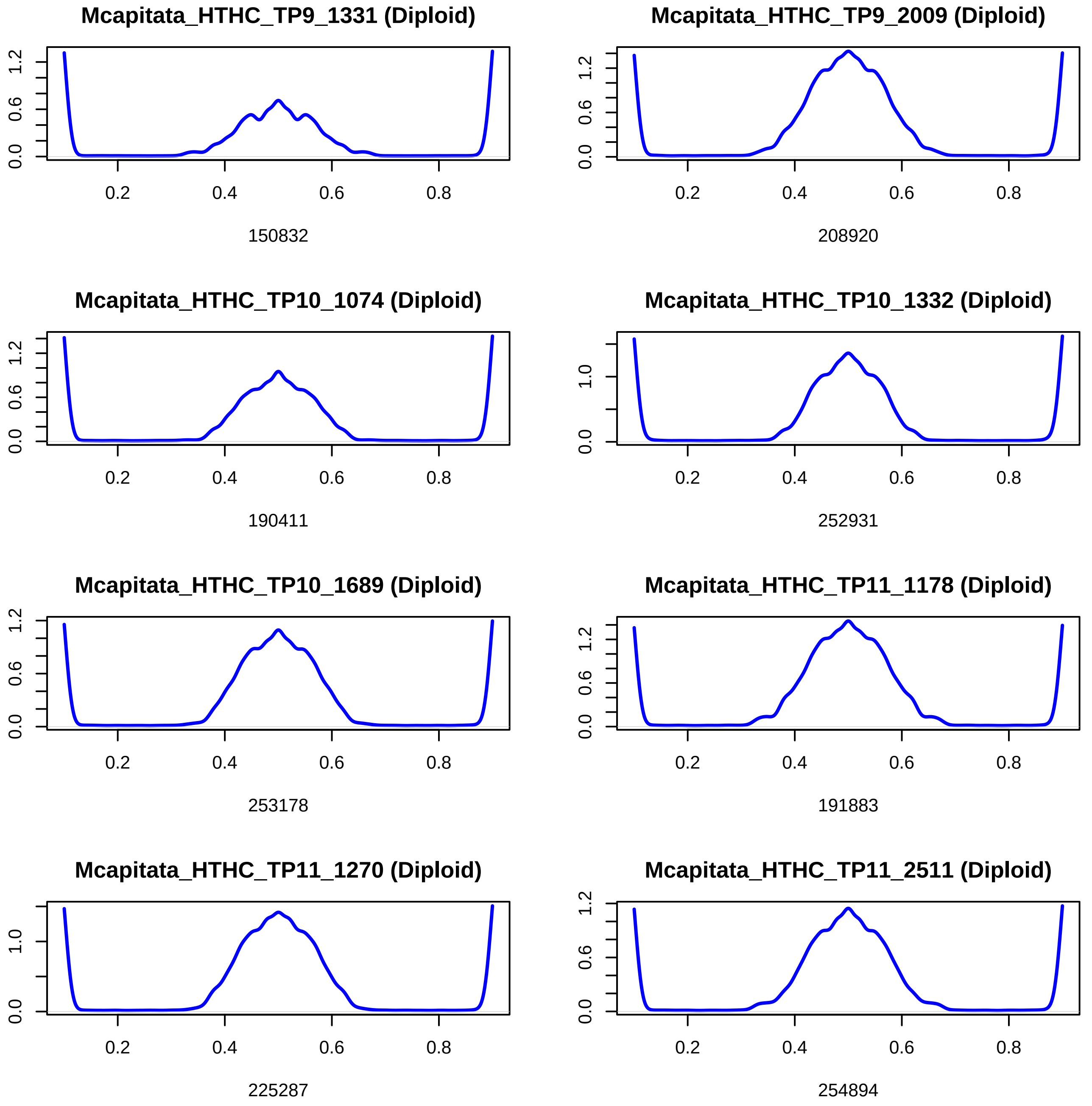
Mcapitata_HTHC_TP9_1331 (Diploid)
150832
Mcapitata_HTHC_TP9_2009 (Diploid)
208920
Mcapitata_HTHC_TP10_1074 (Diploid)
190411
Mcapitata_HTHC_TP10_1332 (Diploid)
252931
Mcapitata_HTHC_TP10_1689 (Diploid)
253178
Mcapitata_HTHC_TP11_1178 (Diploid)
191883
Mcapitata_HTHC_TP11_1270 (Diploid)
225287
Mcapitata_HTHC_TP11_2511 (Diploid)
254894
0.2
0.4
0.6
0.8
0.0
0.6
1.2
1.0

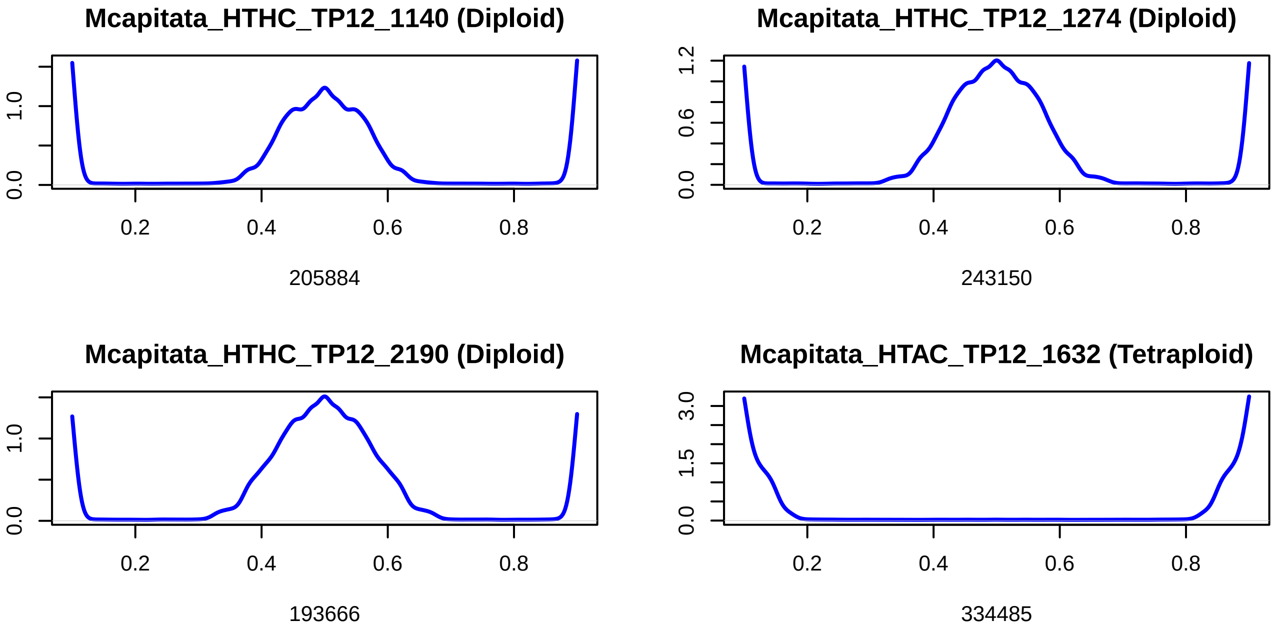
Mcapitata_HTHC_TP12_1140 (Diploid)
0.0
1.0
0.2
0.4
0.6
0.8
205884
Mcapitata_HTHC_TP12_1274 (Diploid)
0.0
0.6
1.2
0.2
0.4
0.6
0.8
243150
Mcapitata_HTHC_TP12_2190 (Diploid)
0.0
1.0
0.2
0.4
0.6
0.8
193666
Mcapitata_HTAC_TP12_1632 (Tetraploid)
0.0
1.5
3.0
0.2
0.4
0.6
0.8
334485
