# Supplementary material for: Ploidy Variation and Its Implications for Reproduction and Population Dynamics in Two Sympatric Hawaiian Coral Species
Source: Genome Biol Evol. 2023 Aug 11;15(8):evad149. doi: 10.1093/gbe/evad149 (PMC10445776; doi:10.1093/gbe/evad149)
Supplement: evad149_Supplementary_Data [file evad149_supplementary_data.zip › Data_S4.pdf]

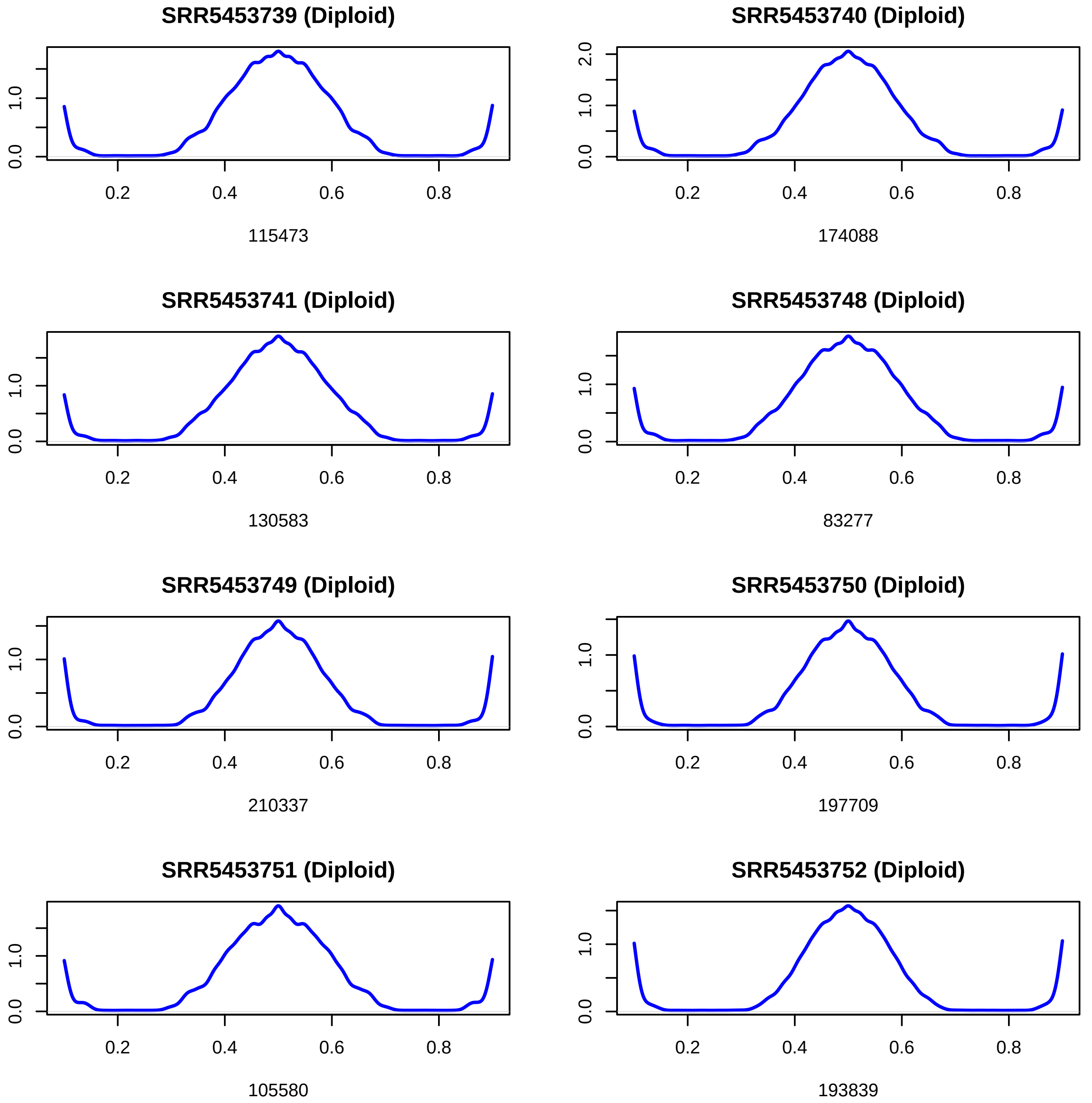

SRR5453739 (Diploid)
0.0
1.0
0.2
0.4
0.6
0.8
115473
SRR5453740 (Diploid)
0.0
1.0
2.0
0.2
0.4
0.6
0.8
174088
SRR5453741 (Diploid)
0.0
1.0
0.2
0.4
0.6
0.8
130583
SRR5453748 (Diploid)
0.0
1.0
0.2
0.4
0.6
0.8
83277
SRR5453749 (Diploid)
0.0
1.0
0.2
0.4
0.6
0.8
210337
SRR5453750 (Diploid)
0.0
1.0
0.2
0.4
0.6
0.8
197709
SRR5453751 (Diploid)
0.0
1.0
0.2
0.4
0.6
0.8
105580
SRR5453752 (Diploid)
0.0
1.0
0.2
0.4
0.6
0.8
193839

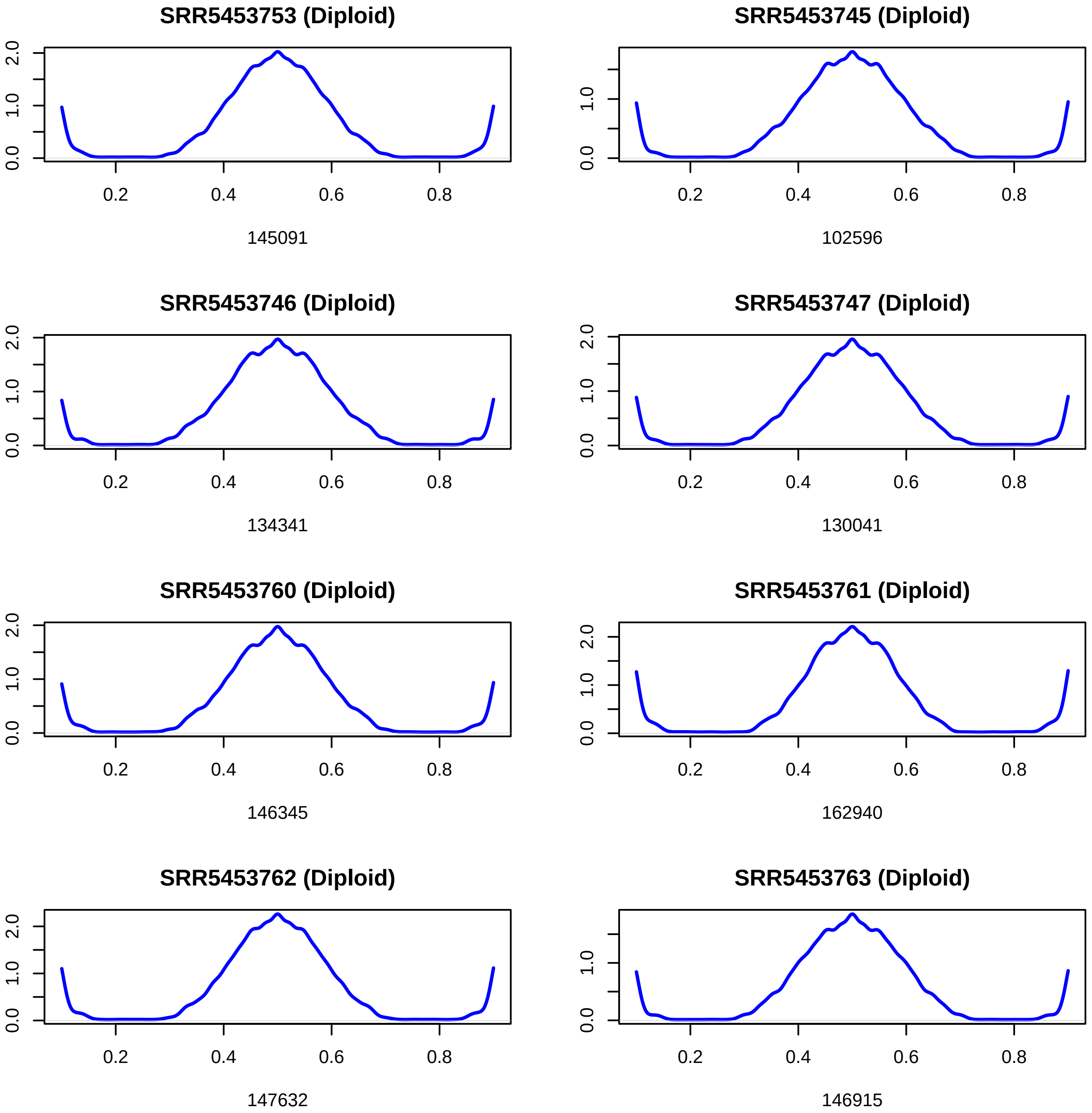
SRR5453753 (Diploid)
145091
SRR5453745 (Diploid)
102596
SRR5453746 (Diploid)
134341
SRR5453747 (Diploid)
130041
SRR5453760 (Diploid)
146345
SRR5453761 (Diploid)
162940
SRR5453762 (Diploid)
147632
SRR5453763 (Diploid)
146915
0.0
1.0
2.0
0.2
0.4
0.6
0.8

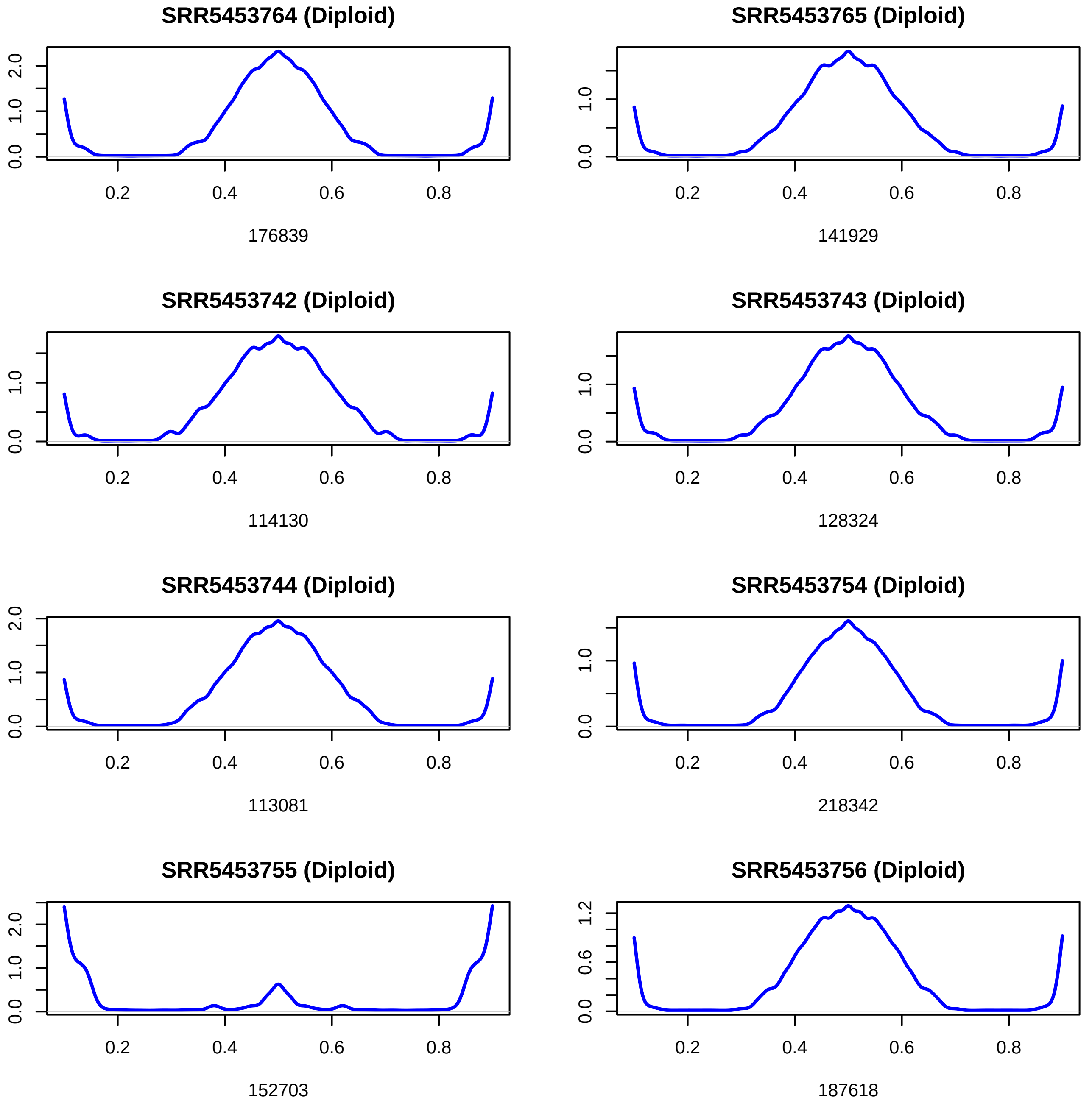
SRR5453764 (Diploid)
176839
SRR5453765 (Diploid)
141929
SRR5453742 (Diploid)
114130
SRR5453743 (Diploid)
128324
SRR5453744 (Diploid)
113081
SRR5453754 (Diploid)
218342
SRR5453755 (Diploid)
152703
SRR5453756 (Diploid)
187618
0.2
0.4
0.6
0.8
0.0
0.6
1.0
1.2
2.0

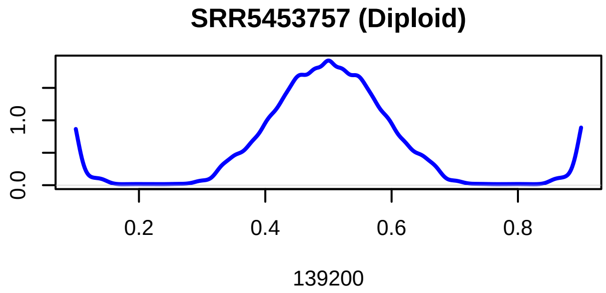
SRR5453757 (Diploid)
0.0
1.0
0.2
0.4
0.6
0.8
139200

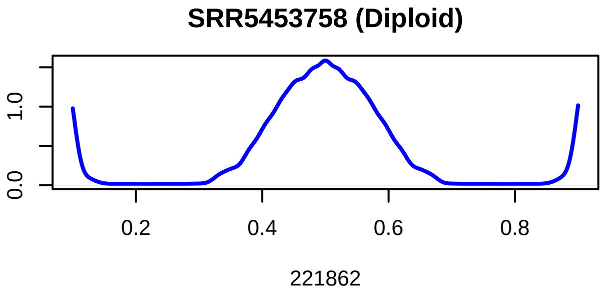
SRR5453758 (Diploid)
0.0
1.0
0.2
0.4
0.6
0.8
221862

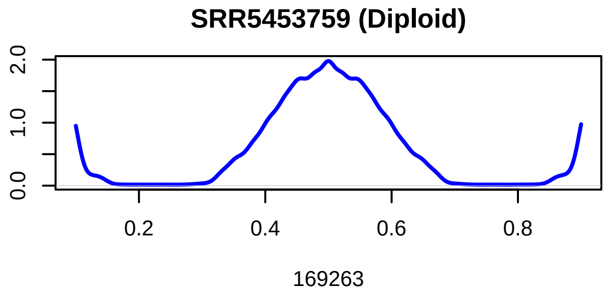
SRR5453759 (Diploid)
0.0
1.0
2.0
0.2
0.4
0.6
0.8
169263
