# Supplementary figures and images for: Ploidy Variation and Its Implications for Reproduction and Population Dynamics in Two Sympatric Hawaiian Coral Species
Source: Genome Biol Evol. 2023 Aug 11;15(8):evad149. doi: 10.1093/gbe/evad149 (PMC10445776; doi:10.1093/gbe/evad149)

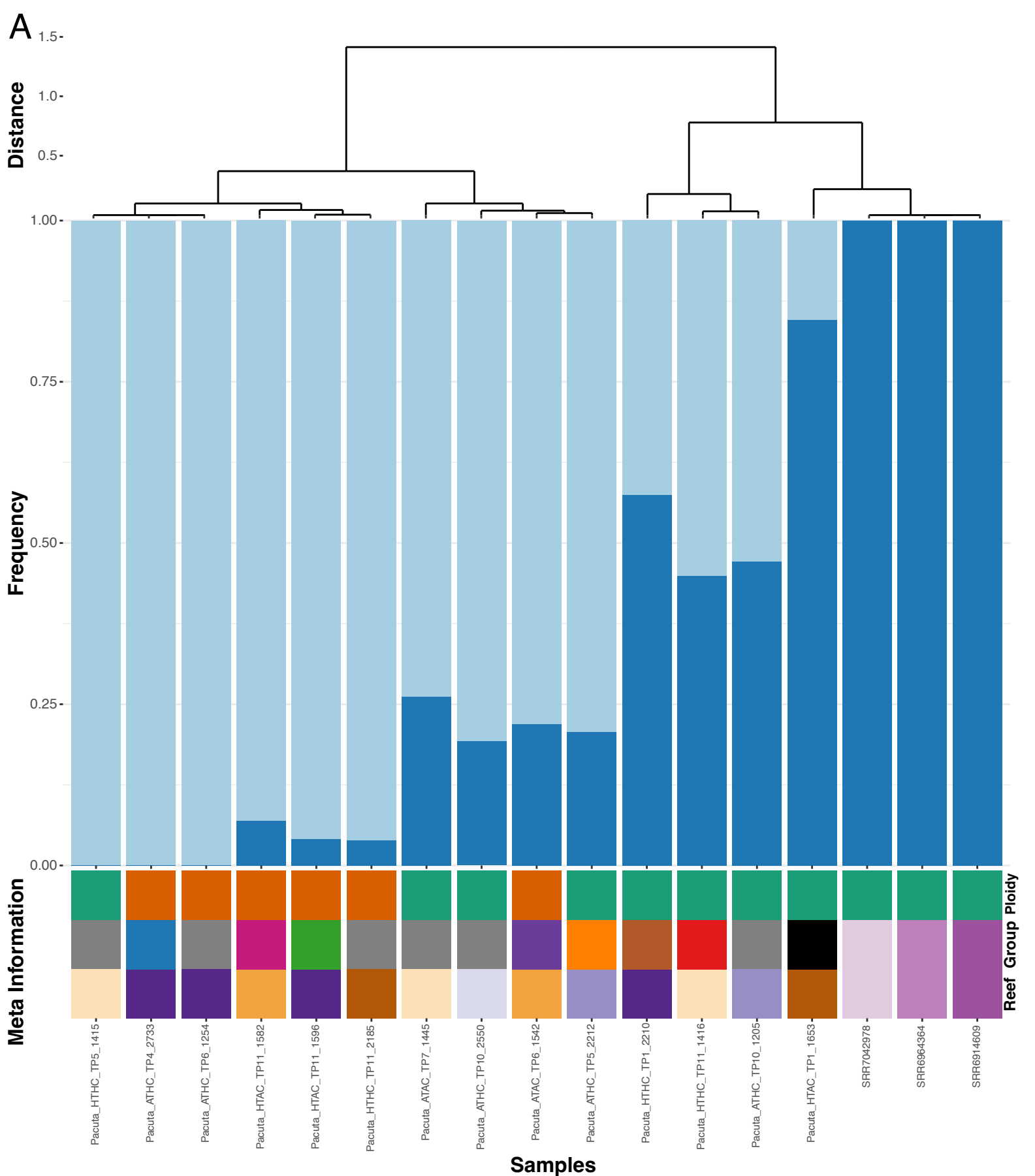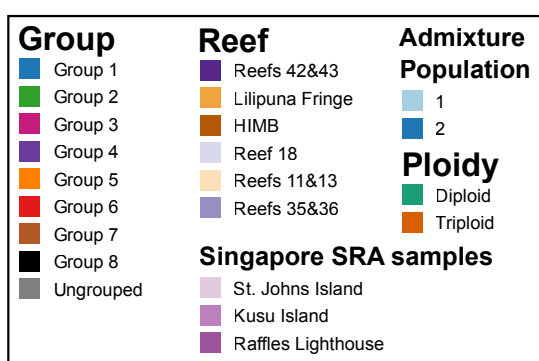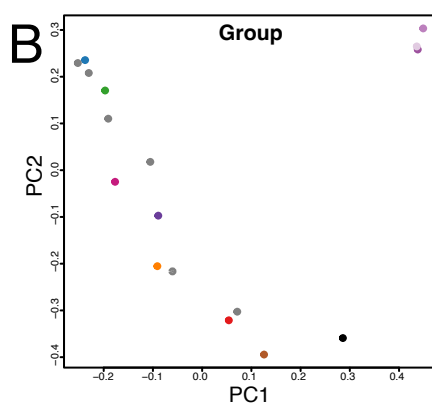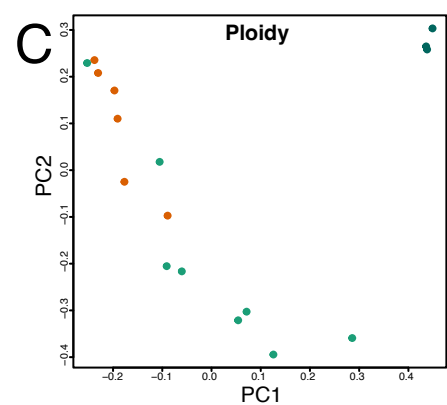

Supplement: evad149_Supplementary_Data [file evad149_supplementary_data.zip › Figure_S4.pdf]

**A**

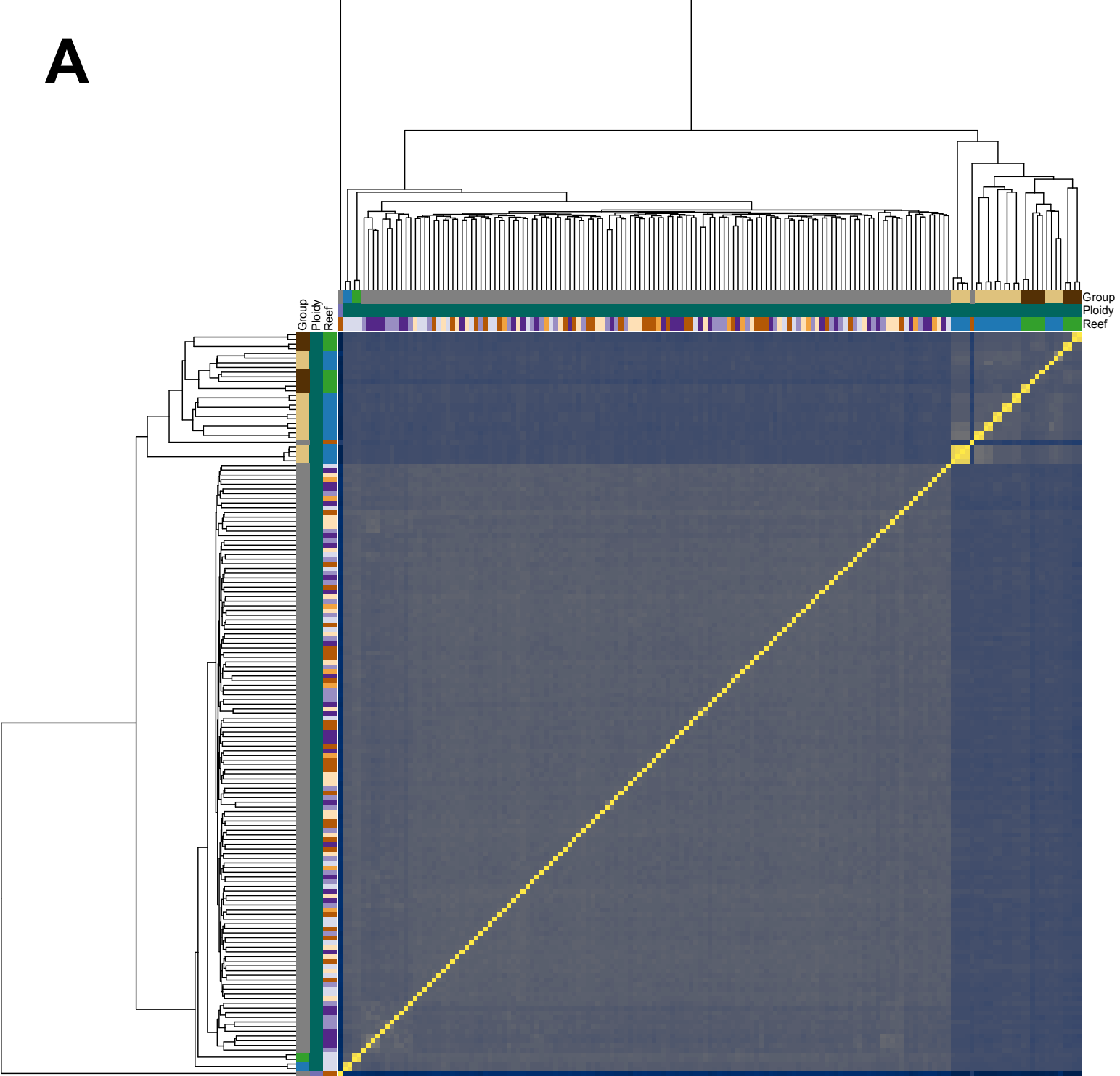

**B**

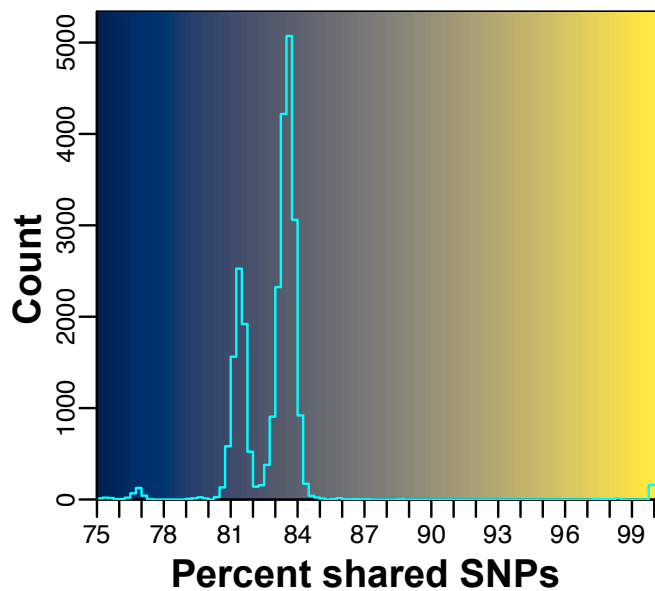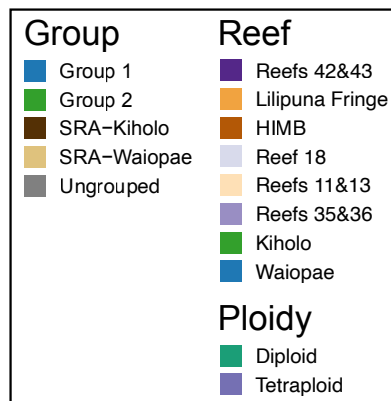

Supplement: evad149_Supplementary_Data [file evad149_supplementary_data.zip › Figure_S5.pdf]

# A

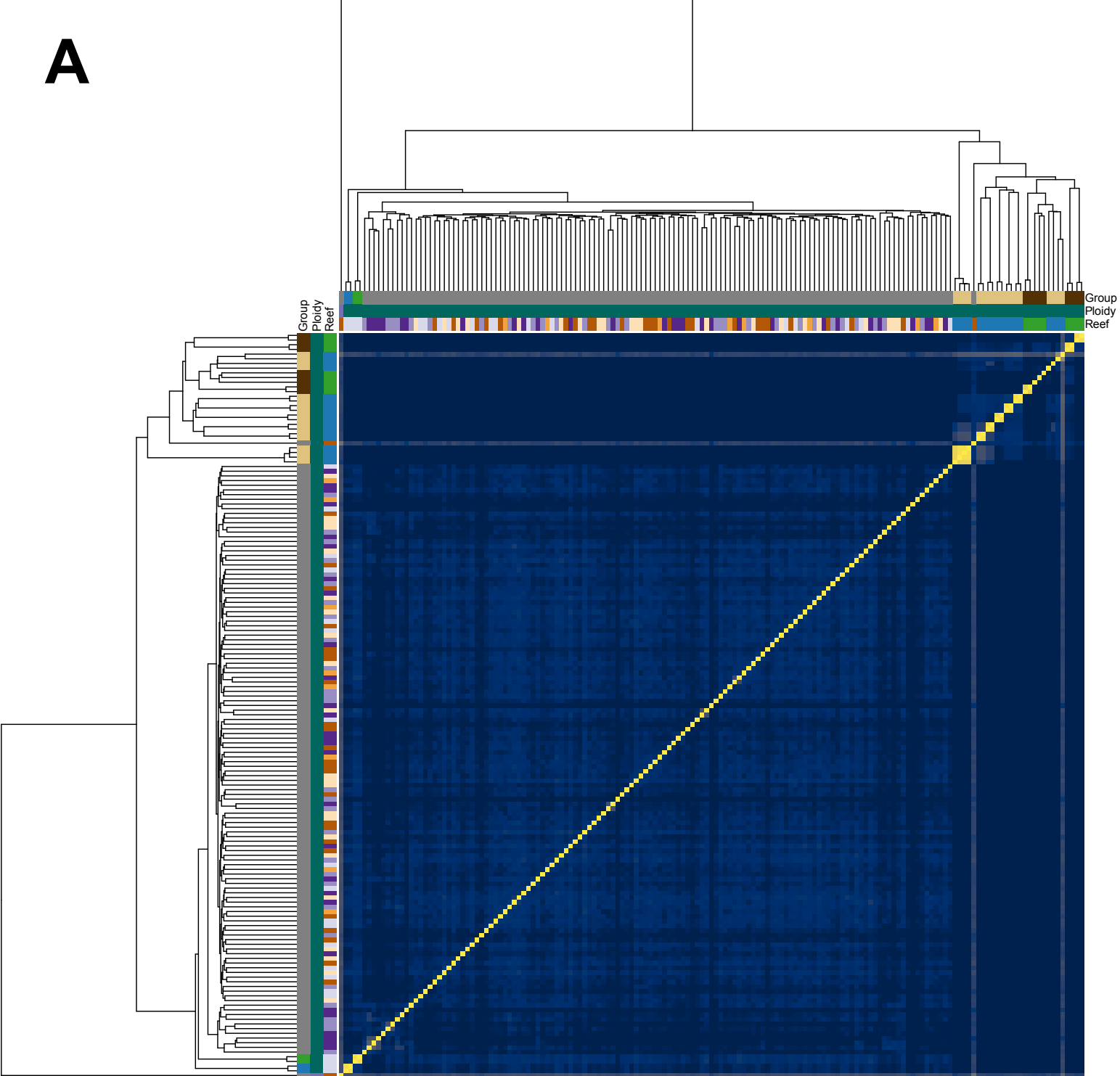

# B

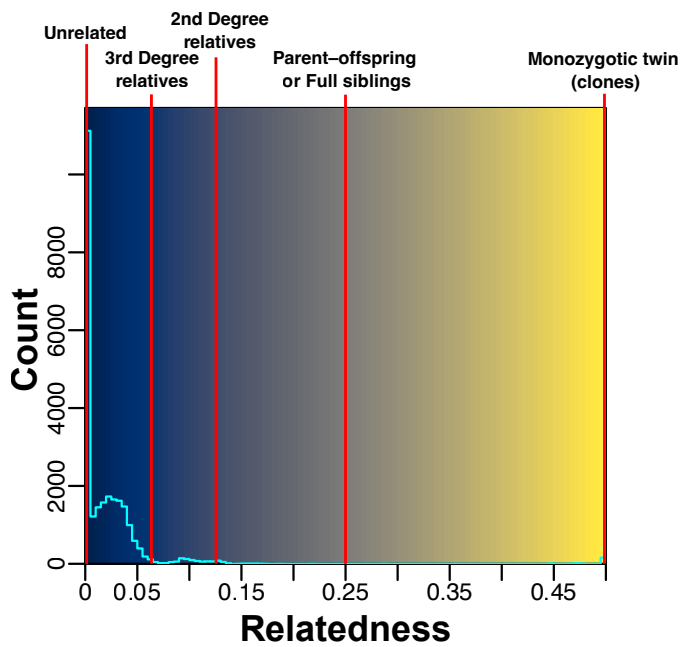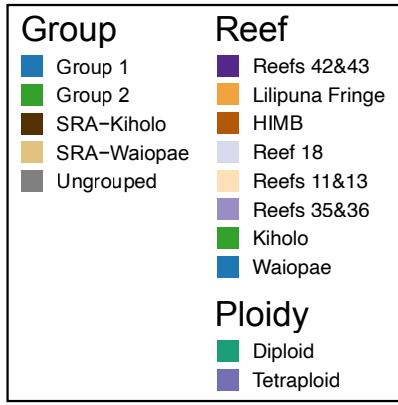

Supplement: evad149_Supplementary_Data [file evad149_supplementary_data.zip › Figure_S6.pdf]

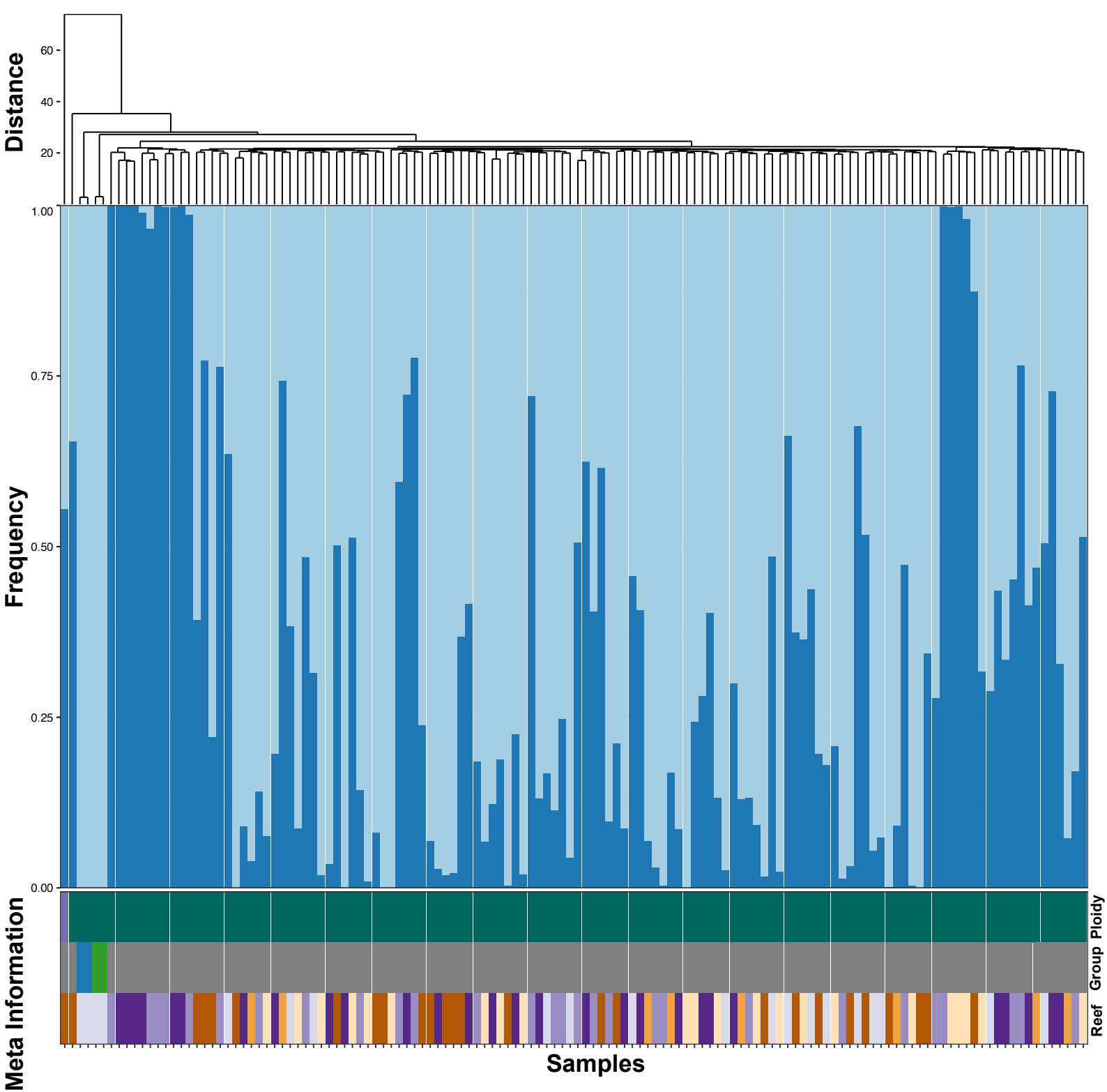

Supplement: evad149_Supplementary_Data [file evad149_supplementary_data.zip › Figure_S7.pdf]

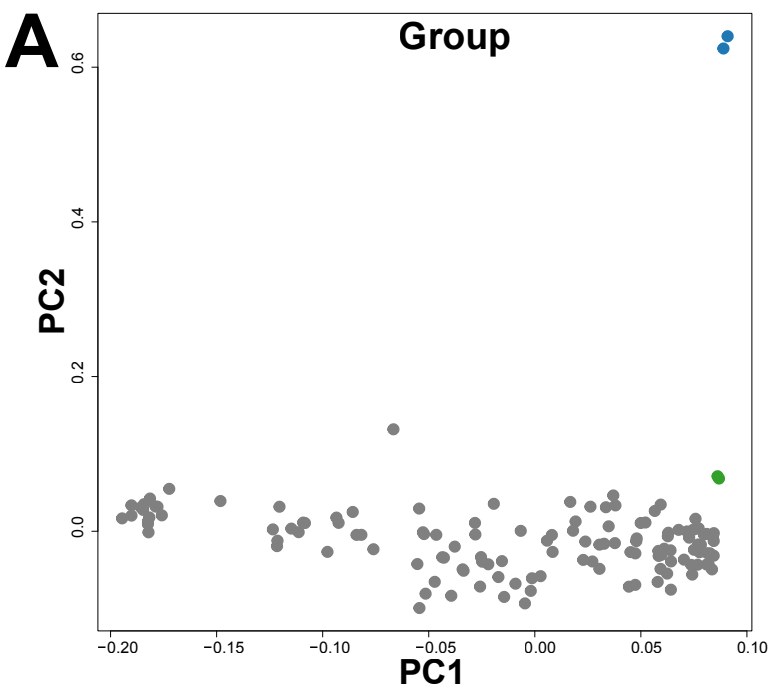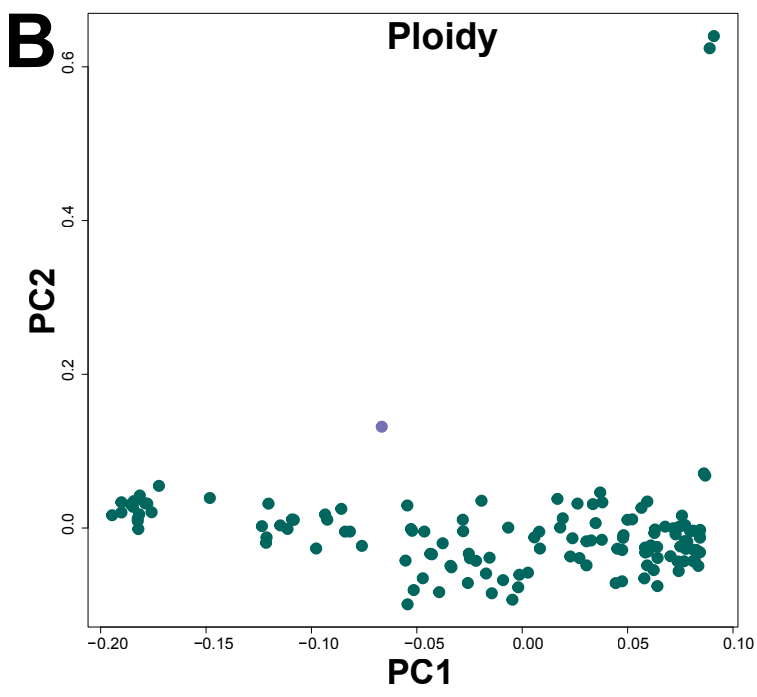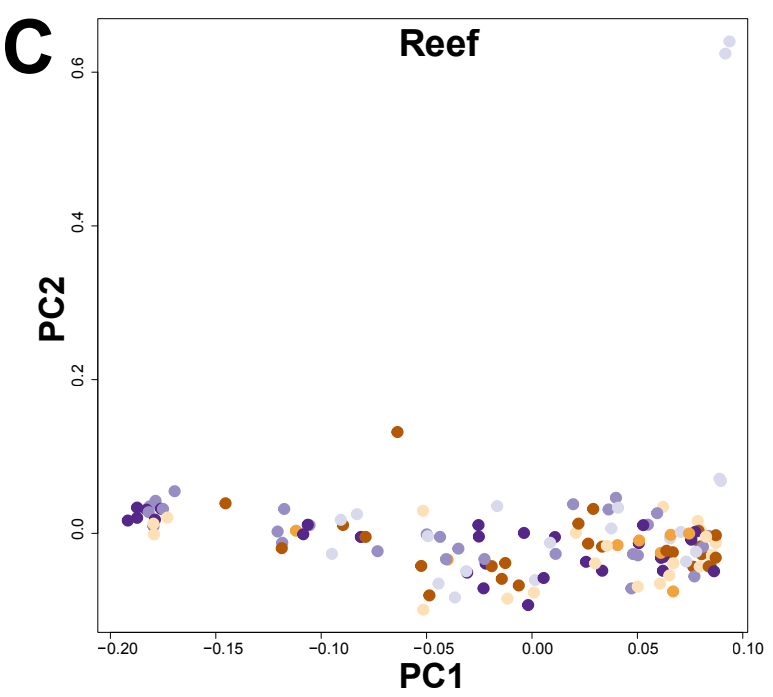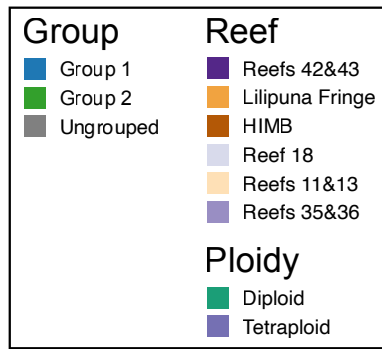

Supplement: evad149_Supplementary_Data [file evad149_supplementary_data.zip › Figure_S8.pdf]

**A**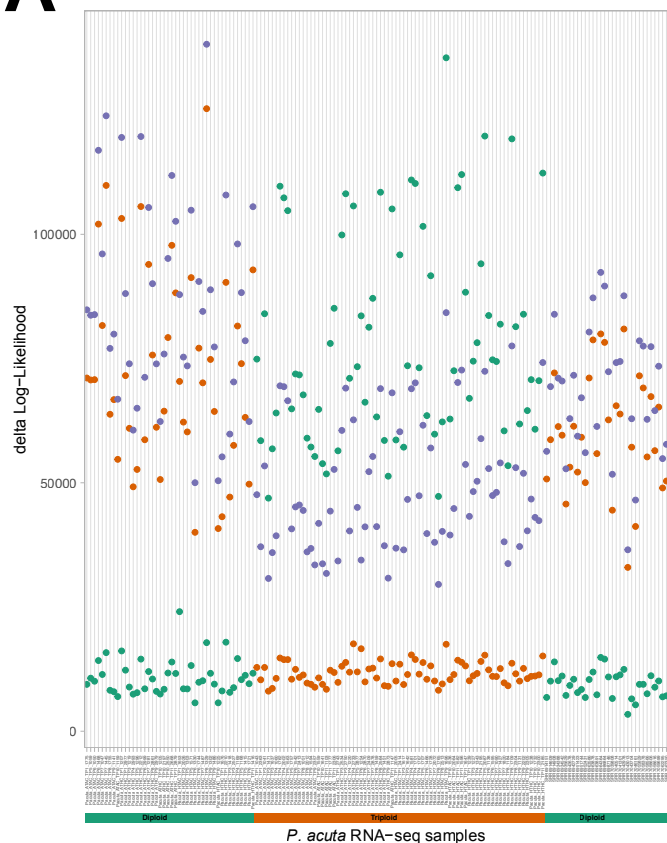**B**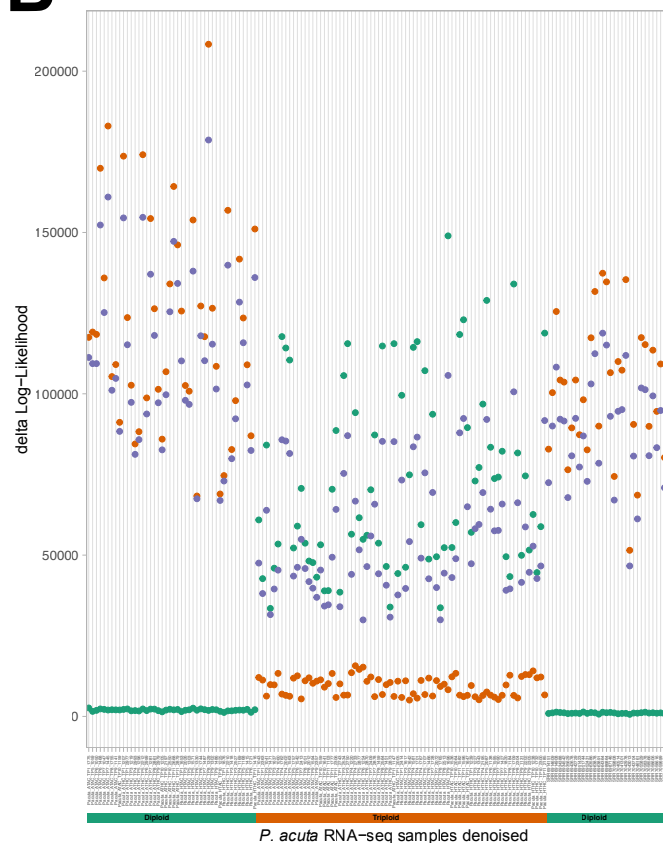**C**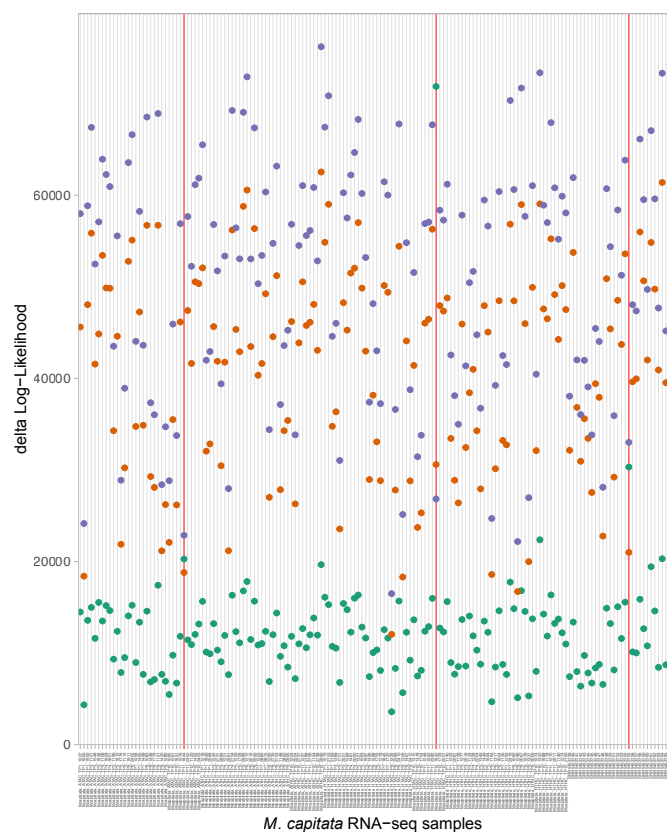**D**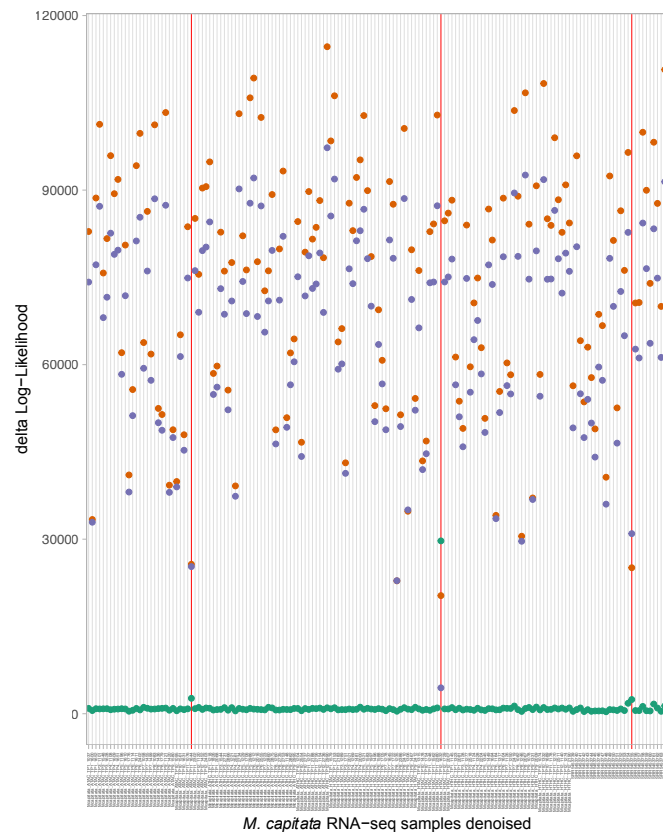**E**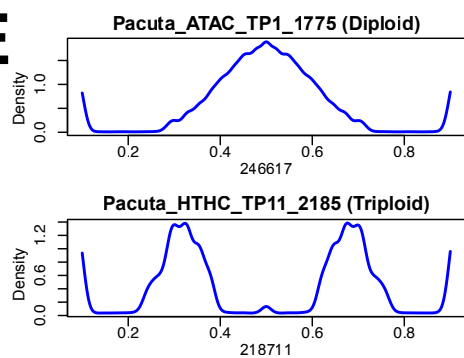**F**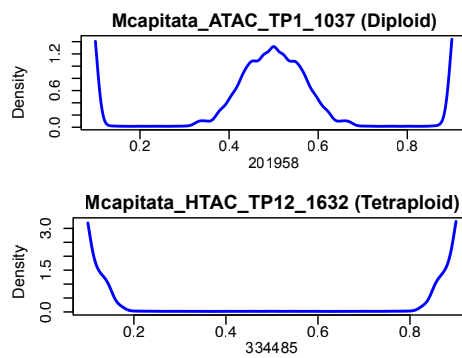

**Ploidy**

- Diploid
- Triploid
- Tetraploid

Supplement: evad149_Supplementary_Data [file evad149_supplementary_data.zip › Figure_S1.pdf]

# A

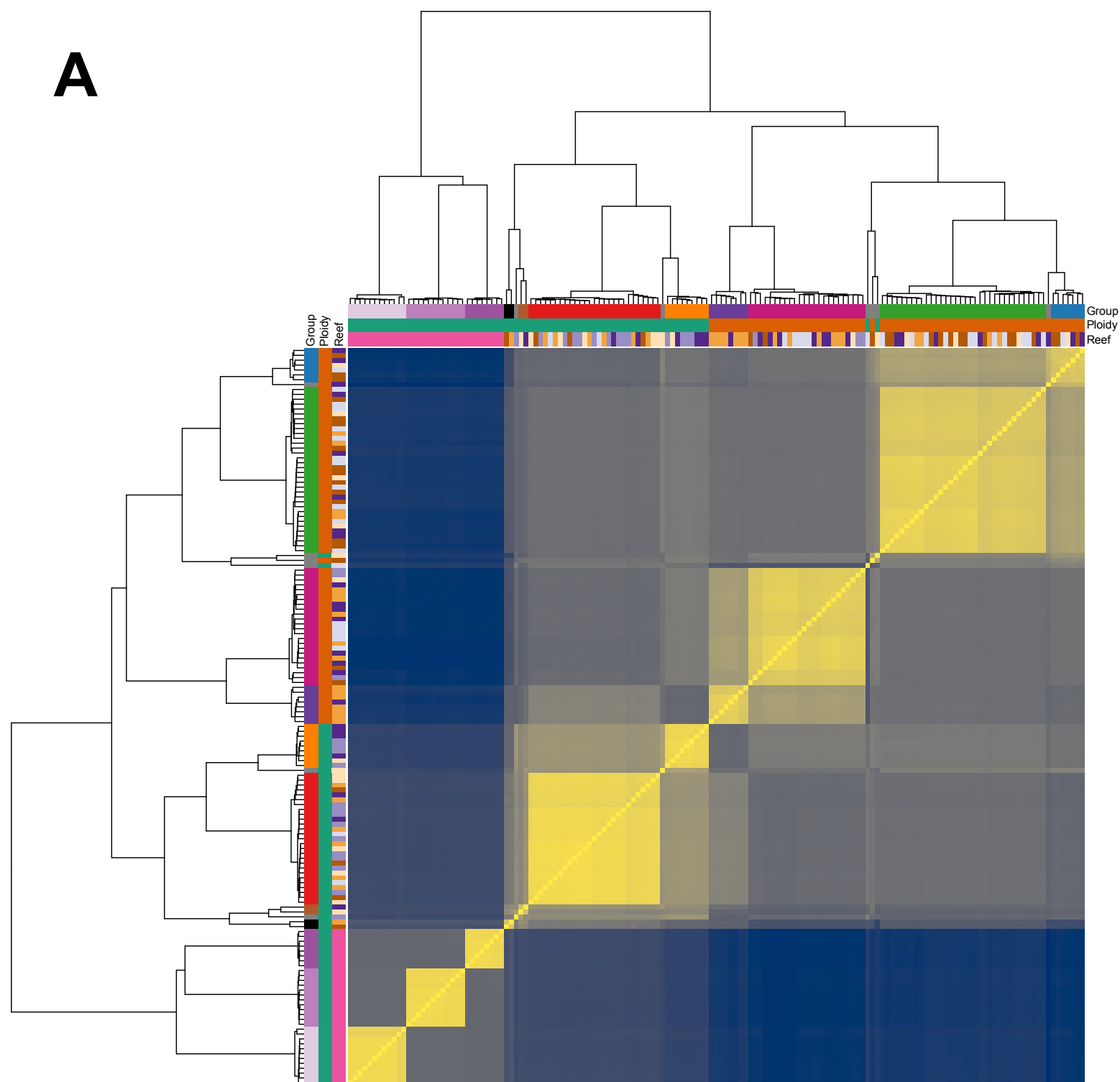

# B

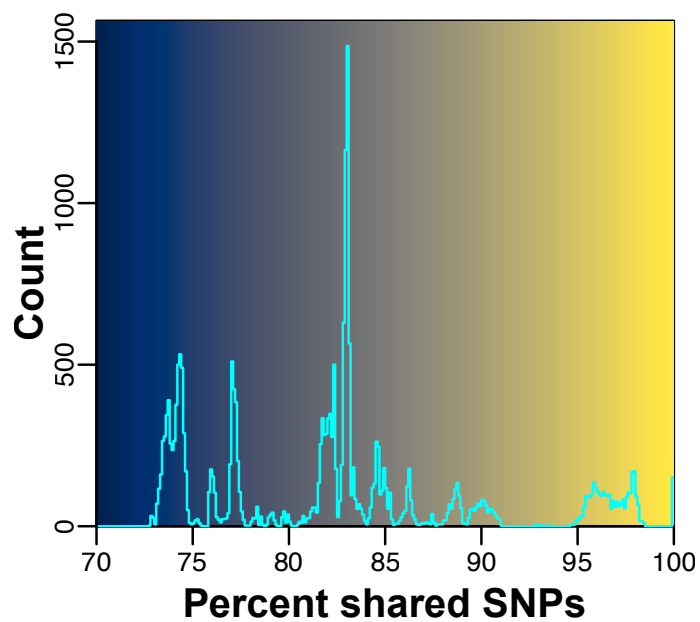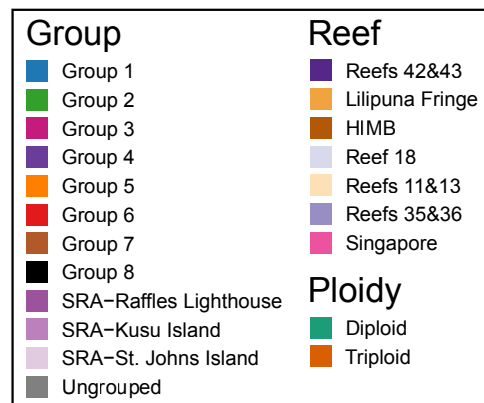

Supplement: evad149_Supplementary_Data [file evad149_supplementary_data.zip › Figure_S2.pdf]

# A

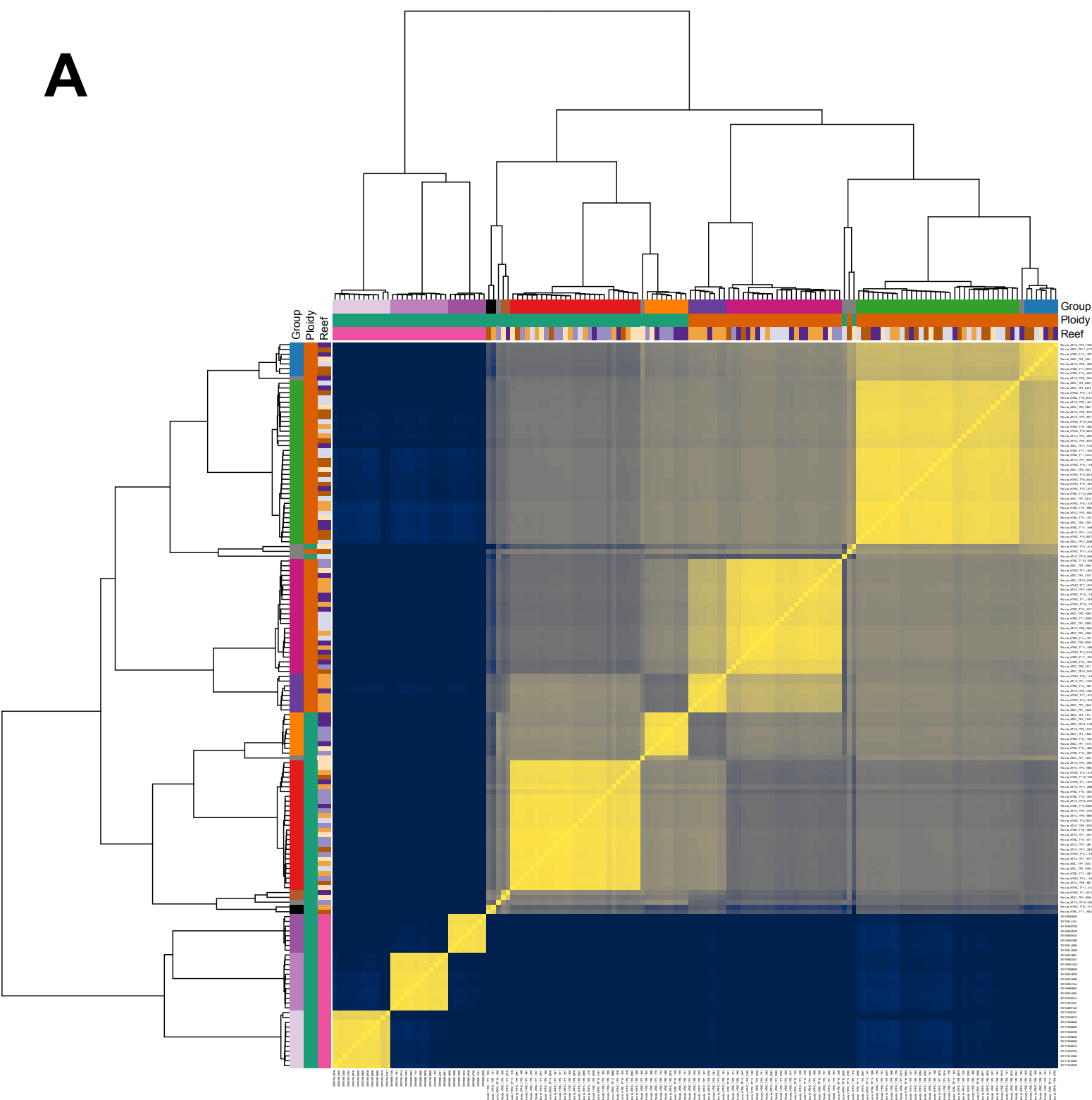

# B

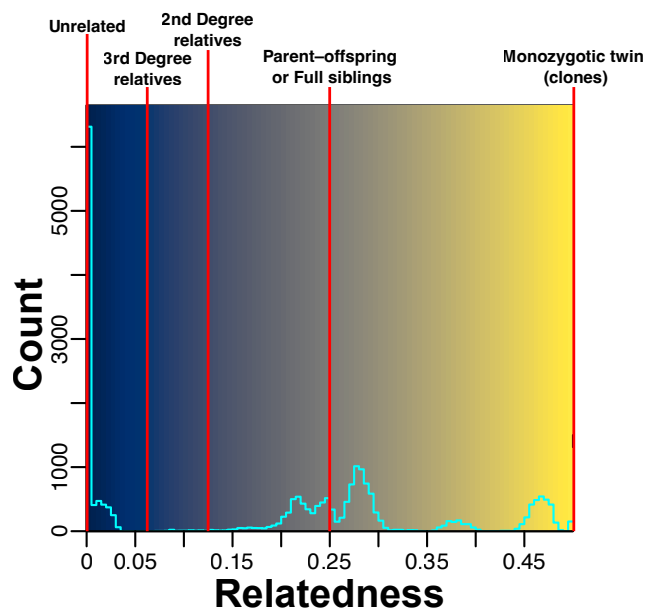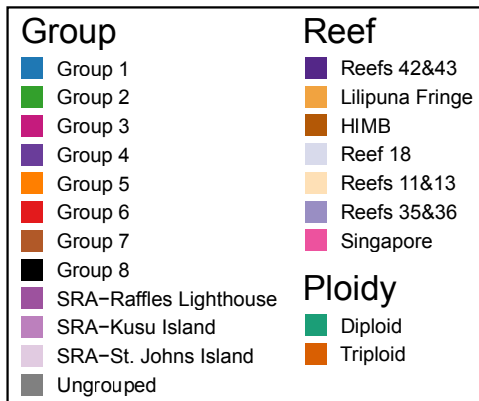

Supplement: evad149_Supplementary_Data [file evad149_supplementary_data.zip › Figure_S3.pdf]
